# GWAS and colocalization analyses implicate carotid intima-media thickness and carotid plaque loci in cardiovascular outcomes

**DOI:** 10.1038/s41467-018-07340-5

**Published:** 2018-12-03

**Authors:** Nora Franceschini, Claudia Giambartolomei, Paul S. de Vries, Chris Finan, Joshua C. Bis, Rachael P. Huntley, Ruth C. Lovering, Salman M. Tajuddin, Thomas W. Winkler, Misa Graff, Maryam Kavousi, Caroline Dale, Albert V. Smith, Edith Hofer, Elisabeth M. van Leeuwen, Ilja M. Nolte, Lingyi Lu, Markus Scholz, Muralidharan Sargurupremraj, Niina Pitkänen, Oscar Franzén, Peter K. Joshi, Raymond Noordam, Riccardo E. Marioni, Shih-Jen Hwang, Solomon K. Musani, Ulf Schminke, Walter Palmas, Aaron Isaacs, Adolfo Correa, Alan B. Zonderman, Albert Hofman, Alexander Teumer, Amanda J. Cox, André G. Uitterlinden, Andrew Wong, Andries J. Smit, Anne B. Newman, Annie Britton, Arno Ruusalepp, Bengt Sennblad, Bo Hedblad, Bogdan Pasaniuc, Brenda W. Penninx, Carl D. Langefeld, Christina L. Wassel, Christophe Tzourio, Cristiano Fava, Damiano Baldassarre, Daniel H. O’Leary, Daniel Teupser, Diana Kuh, Elena Tremoli, Elmo Mannarino, Enzo Grossi, Eric Boerwinkle, Eric E. Schadt, Erik Ingelsson, Fabrizio Veglia, Fernando Rivadeneira, Frank Beutner, Ganesh Chauhan, Gerardo Heiss, Harold Snieder, Harry Campbell, Henry Völzke, Hugh S. Markus, Ian J. Deary, J. Wouter Jukema, Jacqueline de Graaf, Jacqueline Price, Janne Pott, Jemma C. Hopewell, Jingjing Liang, Joachim Thiery, Jorgen Engmann, Karl Gertow, Kenneth Rice, Kent D. Taylor, Klodian Dhana, Lambertus A. L. M. Kiemeney, Lars Lind, Laura M. Raffield, Lenore J. Launer, Lesca M. Holdt, Marcus Dörr, Martin Dichgans, Matthew Traylor, Matthias Sitzer, Meena Kumari, Mika Kivimaki, Mike A. Nalls, Olle Melander, Olli Raitakari, Oscar H. Franco, Oscar L. Rueda-Ochoa, Panos Roussos, Peter H. Whincup, Philippe Amouyel, Philippe Giral, Pramod Anugu, Quenna Wong, Rainer Malik, Rainer Rauramaa, Ralph Burkhardt, Rebecca Hardy, Reinhold Schmidt, Renée de Mutsert, Richard W. Morris, Rona J. Strawbridge, S. Goya Wannamethee, Sara Hägg, Sonia Shah, Stela McLachlan, Stella Trompet, Sudha Seshadri, Sudhir Kurl, Susan R. Heckbert, Susan Ring, Tamara B. Harris, Terho Lehtimäki, Tessel E. Galesloot, Tina Shah, Ulf de Faire, Vincent Plagnol, Wayne D. Rosamond, Wendy Post, Xiaofeng Zhu, Xiaoling Zhang, Xiuqing Guo, Yasaman Saba, MEGASTROKE Consortium, Yukinori Okada, Aniket Mishra, Loes Rutten-Jacobs, Anne-Katrin Giese, Sander W. van der Laan, Solveig Gretarsdottir, Christopher D. Anderson, Michael Chong, Hieab H. H. Adams, Tetsuro Ago, Peter Almgren, Philippe Amouyel, Hakan Ay, Traci M. Bartz, Oscar R. Benavente, Steve Bevan, Giorgio B. Boncoraglio, Robert D. Brown, Adam S. Butterworth, Caty Carrera, Cara L. Carty, Daniel I. Chasman, Wei-Min Chen, John W. Cole, Ioana Cotlarciuc, Carlos Cruchaga, John Danesh, Paul I. W. de Bakker, Anita L. DeStefano, Marcel den Hoed, Qing Duan, Stefan T. Engelter, Guido J. Falcone, Rebecca F. Gottesman, Raji P. Grewal, Stefan Gustafsson, Jeffrey Haessler, Tamara B. Harris, Ahamad Hassan, Aki S. Havulinna, Elizabeth G. Holliday, George Howard, Fang-Chi Hsu, Hyacinth I. Hyacinth, M. Arfan Ikram, Marguerite R. Irvin, Xueqiu Jian, Jordi Jiménez-Conde, Julie A. Johnson, J. Wouter Jukema, Masahiro Kanai, Keith L. Keene, Brett M. Kissela, Dawn O. Kleindorfer, Charles Kooperberg, Michiaki Kubo, Leslie Lange, Carl D. Langefeld, Claudia Langenberg, Jin-Moo Lee, Robin Lemmens, Didier Leys, Cathryn M. Lewis, Wei-Yu Lin, Arne G. Lindgren, Erik Lorentzen, Patrik K. Magnusson, Jane Maguire, Ani Manichaikul, Patrick F. McArdle, James F. Meschia, Thomas H. Mosley, Toshiharu Ninomiya, Martin J. O’Donnell, Sara L. Pulit, Kristiina Rannikmäe, Alexander P. Reiner, Kathryn M. Rexrode, Stephen S. Rich, Paul M. Ridker, Natalia S. Rost, Peter M. Rothwell, Tatjana Rundek, Ralph L. Sacco, Saori Sakaue, Michele M. Sale, Veikko Salomaa, Bishwa R. Sapkota, Reinhold Schmidt, Carsten O. Schmidt, Ulf Schminke, Pankaj Sharma, Agnieszka Slowik, Cathie L. M. Sudlow, Christian Tanislav, Turgut Tatlisumak, Vincent N. S. Thijs, Gudmar Thorleifsson, Unnur Thorsteinsdottir, Steffen Tiedt, Stella Trompet, Matthew Walters, Nicholas J. Wareham, Sylvia Wassertheil-Smoller, Kerri L. Wiggins, Qiong Yang, Salim Yusuf, Tomi Pastinen, Arno Ruusalepp, Eric E. Schadt, Simon Koplev, Veronica Codoni, Mete Civelek, Nick Smith, David A. Trégouët, Ingrid E. Christophersen, Carolina Roselli, Steven A. Lubitz, Patrick T. Ellinor, E. Shyong Tai, Jaspal S. Kooner, Norihiro Kato, Jiang He, Pim van der Harst, Paul Elliott, John C. Chambers, Fumihiko Takeuchi, Andrew D. Johnson, Dharambir K. Sanghera, Olle Melander, Christina Jern, Daniel Strbian, Israel Fernandez-Cadenas, W. T. Longstreth, Arndt Rolfs, Jun Hata, Daniel Woo, Jonathan Rosand, Guillaume Pare, Danish Saleheen, Kari Stefansson, Bradford B. Worrall, Steven J. Kittner, Joanna M. M. Howson, Yoichiro Kamatani, Abbas Dehghan, Adrie Seldenrijk, Alanna C. Morrison, Anders Hamsten, Bruce M. Psaty, Cornelia M. van Duijn, Deborah A. Lawlor, Dennis O. Mook-Kanamori, Donald W. Bowden, Helena Schmidt, James F. Wilson, James G. Wilson, Jerome I. Rotter, Joanna M. Wardlaw, John Deanfield, Julian Halcox, Leo-Pekka Lyytikäinen, Markus Loeffler, Michele K. Evans, Stéphanie Debette, Steve E. Humphries, Uwe Völker, Vilmundur Gudnason, Aroon D. Hingorani, Johan L. M. Björkegren, Juan P. Casas, Christopher J. O’Donnell

**Affiliations:** 1 Department of Epidemiology, University of North Carolina, Chapel Hill, NC 27516 USA; 2 Department of Pathology and Laboratory Medicine, University of California (UCLA), Los Angeles, Los Angeles, CA 90095 USA; 3 Human Genetics Center, Department of Epidemiology, Human Genetics, and Environmental Sciences, School of Public Health, The University of Texas Health Science Center at Houston, Houston, TX 77030 USA; 4 Institute of Cardiovascular Science, University College London, London, WC1 6BT UK; 5 Cardiovascular Health Research Unit, Department of Medicine, University of Washington, Seattle, WA 98101 USA; 6 Laboratory of Epidemiology and Population Sciences, National Institute on Aging, National Institutes of Health, Baltimore, MD 20892 USA; 7 Department of Genetic Epidemiology, University of Regensburg, Regensburg, 93053 Germany; 8 Department of Epidemiology, Erasmus Medical Center, Rotterdam, 3015 The Netherlands; 9 Institute of Health Informatics, University College London, London, WC1E 6BT UK; 10 Icelandic Heart Association, Kopavogur, IS-201 Iceland; 11 University of Iceland, Reykjavik, 101 Iceland; 12 Clinical Division of Neurogeriatrics, Department of Neurology, Medical University of Graz, Graz, 8036 Austria; 13 Institute for Medical Informatics, Statistics and Documentation, Medical University of Graz, Graz, 8036 Austria; 14 Department of Epidemiology, University of Groningen, University Medical Center Groningen, Groningen, 3015 The Netherlands; 15 Department of Biostatistical Sciences, Wake Forest University School of Medicine, Winston-Salem, NC 27157 USA; 16 Institute for Medical Informatics, Statistics and Epidemiology, , University of Leipzig, Leipzig, 04107 Germany; 17 LIFE Research Center for Civilization Diseases, University of Leipzig, Leipzig, 04107 Germany; 18 Univ. Bordeaux, Inserm, Bordeaux Population Health Research Center, UMR 1219, CHU Bordeaux, F-33000 Bordeaux, France; 19 Research Centre of Applied and Preventive Cardiovascular Medicine, University of Turku, Turku, 20520 Finland; 20 Department of Genetics and Genomic Sciences, The Icahn Institute for Genomics and Multiscale Biology Icahn School of Medicine at Mount Sinai, New York, NY 10029 USA; 21 Clinical Gene Networks AB, Stockholm, 104 62 Sweden; 22 Usher Institute of Population Health Sciences and Informatics, University of Edinburgh, Edinburgh, EH8 9AG UK; 23 Department of Internal Medicine, Section of Gerontology and Geriatrics, Leiden University Medical Center, Leiden, 2300 RC The Netherlands; 24 Centre for Cognitive Ageing and Cognitive Epidemiology, University of Edinburgh, Edinburgh, EH8 9JZ UK; 25 Medical Genetics Section, Centre for Genomic and Experimental Medicine, Institute of Genetics and Molecular Medicine, University of Edinburgh, Edinburgh, EH4 2XU UK; 26 Population Sciences Branch, Division of Intramural Research, NHLBI, NIH, Framingham, MA 01702-5827 USA; 27 National Heart, Lung and Blood Institute’s Intramural Research Program, Framingham Heart Study, Framingham, MA 01702-5827 USA; 28 Department of Medicine, University of Mississippi Medical Center, Jackson, MS 39216 USA; 29 Department of Neurology, University Medicine Greifswald, Greifswald, 17475 Germany; 30 Department of Medicine, Columbia University, New York, NY 10032 USA; 31 Department of Biochemistry, Maastricht Centre for Systems Biology (MaCSBio), CARIM School for Cardiovascular Diseases, Maastricht University, Maastricht, 6229 The Netherlands; 32 Department of Epidemiology, Harvard T.H. Chan School of Public Health, Boston, MA 02115 USA; 33 Institute for Community Medicine, University Medicine Greifswald, Greifswald, 17475 Germany; 34 DZHK (German Center for Cardiovascular Research), partner site Greifswald, Greifswald, 17475 Germany; 35 Center for Diabetes Research, Wake Forest School of Medicine, Winston-Salem, NC 25157 USA; 36 Menzies Health Institute Queensland, Griffith University, Southport, QLD 4222 Australia; 37 Department of Internal Medicine, Erasmus Medical Center, University Medical Center Rotterdam, Rotterdam, 3015 The Netherlands; 38 MRC Unit for Lifelong Health and Ageing at UCL, London, WC1E 6BT UK; 39 Department of Medicine, University of Groningen, University Medical Center Groningen, Groningen, 2300 The Netherlands; 40 Department of Epidemiology, and School of Medicine, Division of Geriatric Medicine, University of Pittsburgh, Pittsburgh, PA 15213 USA; 41 Department of Epidemiology and Public Health, University College London, London, WC1E 6BT UK; 42 Department of Pathophysiology, Institute of Biomedicine and Translation Medicine, University of Tartu, Biomeedikum, Tartu, 51010 Estonia; 43 Department of Cardiac Surgery, Tartu University Hospital, Tartu, 51010 Estonia; 44 Cardiovascular Medicine Unit, Department of Medicine Solna, Karolinska Institutet, Stockholm, 17177 Sweden; 45 Department of Cell and Molecular Biology, National Bioinformatics Infrastructure Sweden, Science for Life Laboratory, Uppsala University, Uppsala, 75108 Sweden; 46 Department of Clinical Sciences in Malmö, Lund University, Malmö, SE-205 02 Sweden; 47 Department of Human Genetics, University of California (UCLA), Los Angeles, CA 90095 USA; 48 Department of Psychiatry, EMGO Institute for Health and Care Research and Neuroscience Campus Amsterdam, VU University Medical Center, Amsterdam, 1081 HL The Netherlands; 49 Applied Sciences, Premier, Inc., Charlotte, NC 28277 USA; 50 Department of Medicine, University of Verona, Verona, 37134 Italy; 51 Department of Medical Biotechnology and Translational Medicine, Università di Milano, Milan, 20133 Italy; 52 Centro Cardiologico Monzino, IRCCS, Milan, 20138 Italy; 53 St. Elizabeth’s Medical Center, Tufts University School of Medicine, Boston, MA 02135 USA; 54 Institute of Laboratory Medicine, University Hospital Munich, LMU Munich, 80539 Germany; 55 Dipartimento di Scienze Farmacologiche e Biomolecolari, Università di Milano, Milan, 20133 Italy; 56 Department of Clinical and Experimental Medicine, Internal Medicine, Angiology and Arteriosclerosis Diseases, University of Perugia, Perugia, 06123 Italy; 57 Centro Diagnostico Italiano, Milan, 20147 Italy; 58 Human Genome Sequencing Center, Baylor College of Medicine, Houston, TX 77030-3411 USA; 59 Department of Medicine, Division of Cardiovascular Medicine, Stanford University School of Medicine, Stanford, CA 94309 USA; 60 Department of Medical Sciences, Molecular Epidemiology, Uppsala University, Uppsala, 75185 Sweden; 61 Stanford Cardiovascular Institute, Stanford University, Stanford, CA G1120 USA; 62 Heart Center Leipzig, Leipzig, 04103 Germany; 63 Centre for Brain Research, Indian Institute of Science, Bangalore, 560012 India; 64 Stroke Research Group, Department of Clinical Neurosciences, University of Cambridge, Cambridge, CB2 0QQ UK; 65 Department of Psychology, University of Edinburgh, Edinburgh, EH8 9JZ UK; 66 Department of Cardiology, Leiden University Medical Center, Leiden, 2300 RC The Netherlands; 67 Department of Internal Medicine, Radboud University Medical Center, Nijmegen, 6525 GA The Netherlands; 68 Clinical Trial Service Unit and Epidemiological Studies Unit, Nuffield Department of Population Health, University of Oxford, Oxford, OX3 7LF UK; 69 Department of Population and Quantitative Health Sciences, School of Medicine, Case Western Reserve University, Cleveland, OH 44106 USA; 70 Institute for Laboratory Medicine, University of Leipzig, Leipzig, 04109 Germany; 71 Department of Biostatistics, University of Washington, Seattle, WA 98105 USA; 72 Institute for Translational Genomics and Population Sciences, Los Angeles Biomedical Research Institute at Harbor-UCLA Medical Center, Torrance, CA 90502 USA; 73 Department of Internal Medicine, Rush University Medical Center, Chicago, IL 60612 USA; 74 Radboud Institute for Health Sciences, Radboud University Medical Center, Nijmegen, GA 6525 The Netherlands; 75 Department of Medical Sciences, Cardiovascular Epidemiology, Uppsala University, Uppsala, 751 05 Sweden; 76 Department of Genetics, University of North Carolina, Chapel Hill, NC 27516 USA; 77 Department of Internal Medicine B, University Medicine Greifswald, Greifswald, 17475 Germany; 78 Institute for Stroke and Dementia Research (ISD), University Hospital, Ludwig-Maximilians-University (LMU), Munich, 80539 Germany; 79 Munich Cluster for Systems Neurology (SyNergy), Munich, 81377 Germany; 80 Department of Neurology, Center for Neurology and Neurosurgery, Johann Wolfgang Goethe-University, Frankfurt am Main, 60323 Germany; 81 Institute for Social and Economic Research, Essex University, Colchester, CO4 3SQ UK; 82 Laboratory of Neurogenetics, National Institute on Aging, National Institutes of Health, Bethesda, MD 20892 USA; 83 Data Tecnica International, Glen Echo, MD 20812 USA; 84 Department of Clinical Physiology and Nuclear Medicine, Turku University Hospital, Turku, 20521 Finland; 85 Institute of Social and Preventive Medicine (ISPM), University of Bern, Bern, 3012 Switzerland; 86 Electrocardiography Research Group, School of Medicine, Universidad Industrial de Santander, Bucaramanga, Santander 680003 Colombia; 87 Department of Psychiatry and Friedman Brain Institute, Icahn School of Medicine at Mount Sinai, New York, NY 10029 USA; 88 Mental Illness Research Education and Clinical Center (MIRECC), James J. Peters VA Medical Center, Bronx, New York, NY 10468 USA; 89 Population Health Research Institute, St George’s, University of London, London, SW17 0RE UK; 90 Inserm U1167, F-59000 Lille, France; 91 Institut Pasteur de Lille, U1167, F-59000 Lille, France; 92 Université de Lille, U1167 - RID-AGE & Centre Hospitalier Universitaire de Lille, U1167, F-59000 Lille, France; 93 Sorbonne Université, Cardiovascular Prevention Unit, Pitié Salpétrière Hospital, Paris, 75013 France; 94 Collaborative Health Studies Coordinating Center, Department of Biostatistics, University of Washington, Seattle, WA 98195 USA; 95 Foundation for Research in Health Exercise and Nutrition, Kuopio Research Institute of Exercise Medicine, Kuopio, 70100 Finland; 96 Department of Clinical Physiology and Nuclear Medicine, Kuopio University Hospital, Kuopio, 70210 Finland; 97 Institute of Laboratory Medicine, University of Leipzig, Leipzig, 04109 Germany; 98 Institute of Clinical Chemistry and Laboratory Medicine, University Hospital Regensburg, Regensburg, 93053 Germany; 99 Department of Clinical Epidemiology, Leiden University Medical Center, Leiden, 2333 The Netherlands; 100 Department of Population Health Sciences, Bristol Medical School, University of Bristol, Bristol, BS8 1QU UK; 101 Mental Health and Wellbeing, Institute of Health and Wellbeing, University of Glasgow, Glasgow, G12 0XH UK; 102 Department of Primary Care & Population Health, University College London, London, WC1E 6BT UK; 103 Department of Medical Epidemiology and Biostatistics, Karolinska Institutet, Stockholm, SE-171 77 Sweden; 104 Department of Neurology, Boston University School of Medicine, Boston, MA 02118 USA; 105 Institute of Public Health and Clinical Nutrition, University of Eastern Finland, Kuopio Campus, Kuopio, FI-70210 Finland; 106 Kaiser Permanente Washington Health Research Institute, Seattle, WA 98101 USA; 107 Population Health Science, Bristol Medical School, University of Bristol, Bristol, BS8 1QU UK; 108 MRC Integrative Epidemiology Unit at the University of Bristol, Bristol, BS8 1TH UK; 109 Department of Clinical Chemistry, Fimlab Laboratories, Tampere, 33014 Finland; 110 Department of Clinical Chemistry, University of Tampere School of Medicine, Tampere, 33014 Finland; 111 Division of Cardiovascular Epidemiology, Institute of Environmental Medicine, Karolinska Institutet, Stockholm, S-171 77 Sweden; 112 Department of Cardiology, Karolinska University Hospital, Stockholm, S-171 77 Sweden; 113 Genetics Institute, University College London, London, WC1E 6BT UK; 114 Departments of Medicine and Epidemiology, Johns Hopkins University, Baltimore, MD 21205 USA; 115 Section of Biomedical Genetics, School of Medicine, Boston University, Boston, MA 02215 USA; 116 Department of Pediatrics, Los Angeles Biomedical Research Institute at Harbor-UCLA Medical Center, Torrance, CA 90502 USA; 117 Institute of Molecular Biology and Biochemistry, Centre for Molecular Medicine, Medical University of Graz, Graz, 8010 Austria; 118 Department of Epidemiology & Biostatistics, Imperial College London, London, SW7 2AZ UK; 119 GGZ inGeest and Amsterdam Public Health Research Institute, Department of Psychiatry, Amsterdam University Medical Center, Amsterdam, 1081 HV The Netherlands; 120 Cardiovascular Health Research Unit and Departments of Medicine, Epidemiology, and Health Services, University of Washington, Seattle, WA 98195 USA; 121 Department of Public Health and Primary Care, Leiden University Medical Center, Leiden, 2333 ZA The Netherlands; 122 Center for Human Genomics, Wake Forest University School of Medicine, Winston-Salem, NC 27157 USA; 123 MRC Human Genetics Unit, Institute of Genetics and Molecular Medicine, University of Edinburgh, Western General Hospital, Edinburgh, EH4 2XU UK; 124 Department of Physiology and Biophysics, University of Mississippi Medical Center, Jackson, MS 39216 USA; 125 Centre for Clinical Brain Sciences, and UK Dementia Research Institute at the University of Edinburgh, Edinburgh, EH16 4SB UK; 126 Swansea University Medical School, Swansea, SA2 8PP UK; 127 Centre for Cardiovascular Genetics, Institute Cardiovascular Science, University College London, London, WC1E 6BT UK; 128 Interfaculty Institute for Genetics and Functional Genomics, University Medicine Greifswald, Greifswald, 17475 Germany; 129 Integrated Cardio Metabolic Centre, Department of Medicine, Karolinska Institutet, Karolinska Universitetssjukhuset, Huddinge, SE-141 57 Sweden; 130 Intramural Administration Management Branch, National Heart, Lung, and Blood Institute, NIH, Bethesda, MD 20892 USA; 131 Cardiology Section, Boston Veteran’s Administration Healthcare, Boston, MA 02130 USA; 132 Harvard Medical School, Boston, MA 02115 USA; 133 Laboratory for Statistical Analysis, RIKEN Center for Integrative Medical Sciences, Yokohama, 230-0045 Japan; 134 Department of Statistical Genetics, Osaka University Graduate School of Medicine, Osaka, 565-0871 Japan; 135 Laboratory of Statistical Immunology, Immunology Frontier Research Center (WPI-IFReC), Osaka University, Suita, 565-0871 Japan; 136 INSERM U1219 Bordeaux Population Health Research Center, Bordeaux, F-33000 France; 137 University of Bordeaux, Bordeaux, F-33000 France; 138 Stroke Research Group, Division of Clinical Neurosciences, University of Cambridge, Cambridge, CB2 1TN UK; 139 Department of Neurology, Massachusetts General Hospital, Harvard Medical School, Boston, MA 02114 USA; 140 J. Philip Kistler Stroke Research Center, Department of Neurology, MGH, Boston, MA 02215 USA; 141 Laboratory of Experimental Cardiology, Division of Heart and Lungs, University Medical Center Utrecht, Utrecht, 3584 CX Netherlands; 142 deCODE genetics/AMGEN inc, Reykjavik, 101 Iceland; 143 Center for Genomic Medicine, Massachusetts General Hospital (MGH), Boston, MA 02114 USA; 144 Program in Medical and Population Genetics, Broad Institute, Cambridge, MA 02142 USA; 145 Population Health Research Institute, McMaster University, Hamilton, L8L 2X2 Canada; 146 Department of Medicine and Clinical Science, Graduate School of Medical Sciences, Kyushu University, Fukuoka, 819-0935 Japan; 147 Albrecht Kossel Institute, University Clinic of Rostock, Rostock, 18147 Germany; 148 INSERM U1167, Institut Pasteur de Lille, Lille, F-59000 France; 149 Department of Public Health, Lille University Hospital, Lille, F-59000 France; 150 Department of Radiology, Massachusetts General Hospital, Harvard Medical School, AA Martinos Center for Biomedical Imaging, Boston, MA 02129 USA; 151 Division of Neurology, Faculty of Medicine, Brain Research Center, University of British Columbia, Vancouver, 170-637 Canada; 152 School of Life Science, University of Lincoln, Lincoln, LN6 7TS UK; 153 Department of Cerebrovascular Diseases, Fondazione IRCCS Istituto Neurologico Carlo Besta, Milano, 20133 Italy; 154 Department of Neurology, Mayo Clinic Rochester, Rochester, MN 55905 USA; 155 MRC/BHF Cardiovascular Epidemiology Unit, Department of Public Health and Primary Care, University of Cambridge, Cambridge, CB2 1TN UK; 156 The National Institute for Health Research Blood and Transplant Research Unit in Donor Health and Genomics, University of Cambridge, Cambridge, CB2 1TN UK; 157 Neurovascular Research Laboratory, Vall d’Hebron Institut of Research, Neurology and Medicine Departments-Universitat Autònoma de Barcelona, Vall d’Hebrón Hospital, Barcelona, 08193 Spain; 158 Stroke Pharmacogenomics and Genetics, Fundacio Docència i Recerca MutuaTerrassa, Terrassa, 08222 Spain; 159 Children’s Research Institute, Children’s National Medical Center, Washington, DC 20052 USA; 160 Center for Translational Science, George Washington University, Washington, DC 20052 USA; 161 Division of Preventive Medicine, Brigham and Women’s Hospital, Boston, MA 02115 USA; 162 Department of Public Health Sciences, Center for Public Health Genomics, University of Virginia School of Medicine, Charlottesville, VA 22904-4259 USA; 163 Department of Neurology, University of Maryland School of Medicine and Baltimore VAMC, Baltimore, MD 21201 USA; 164 Institute of Cardiovascular Research, Royal Holloway University of London, Egham, TW20 OEX UK; 165 Department of Psychiatry,The Hope Center Program on Protein Aggregation and Neurodegeneration (HPAN), Washington University, School of Medicine, St. Louis, MO 98195 USA; 166 Department of Developmental Biology, Washington University School of Medicine, St. Louis, MO 98195 USA; 167 Wellcome Trust Sanger Institute, Hinxton, CB10 1SA UK; 168 Department of Medical Genetics, University Medical Center Utrecht, Utrecht, 3584 CX The Netherlands; 169 Department of Epidemiology, Julius Center for Health Sciences and Primary Care, University Medical Center Utrecht, Utrecht, 3584 CX The Netherlands; 170 Boston University School of Public Health, Boston, MA 02118 USA; 171 Department of Immunology, Genetics and Pathology and Science for Life Laboratory, Uppsala University, Uppsala, 751 05 Sweden; 172 MRC Epidemiology Unit, University of Cambridge School of Clinical Medicine, Institute of Metabolic Science, Cambridge Biomedical Campus, Cambridge, CB2 0SL UK; 173 Department of Neurology and Stroke Center, Basel University Hospital, Basel, 4031 Switzerland; 174 Neurorehabilitation Unit, University and University Center for Medicine of Aging and Rehabilitation Basel, Felix Platter Hospital, Basel, 4055 Switzerland; 175 Department of Neurology, Yale University School of Medicine, New Haven, CT 06510 USA; 176 Department of Neurology, Johns Hopkins University School of Medicine, Baltimore, MD 21205 USA; 177 Neuroscience Institute, SF Medical Center, Trenton, NJ 08629 USA; 178 Division of Public Health Sciences, Fred Hutchinson Cancer Research Center, Seattle, WA 98109-1024 USA; 179 Department of Neurology, Leeds General Infirmary, Leeds Teaching Hospitals NHS Trust, Leeds, LS1 3EX UK; 180 National Institute for Health and Welfare, Helsinki, FI-00271 Finland; 181 FIMM - Institute for Molecular Medicine Finland, Helsinki, FI-00271 Finland; 182 Public Health Stream, Hunter Medical Research Institute, New Lambton, NSW 2305 Australia; 183 Faculty of Health and Medicine, University of Newcastle, Newcastle, 2308 Australia; 184 School of Public Health, University of Alabama at Birmingham, Birmingham, AL 35487 USA; 185 Aflac Cancer and Blood Disorder Center, Department of Pediatrics, Emory University School of Medicine, Atlanta, GA 30322 USA; 186 Epidemiology, School of Public Health, University of Alabama at Birmingham, Birmingham, 35487 USA; 187 Brown Foundation Institute of Molecular Medicine, University of Texas Health Science Center at Houston, Houston, TX 77030 USA; 188 Neurovascular Research Group (NEUVAS), Neurology Department, Institut Hospital del Mar d’Investigació Mèdica, Universitat Autònoma de Barcelona, Barcelona, 08193 Spain; 189 Department of Pharmacotherapy and Translational Research and Center for Pharmacogenomics, University of Florida, College of Pharmacy, Gainesville, FL 32611 USA; 190 Division of Cardiovascular Medicine, College of Medicine, University of Florida, Gainesville, FL 32611 USA; 191 Department of Biology, East Carolina University, Greenville, NC 27858 USA; 192 Center for Health Disparities, East Carolina University, Greenville, NC 27858 USA; 193 University of Cincinnati College of Medicine, Cincinnati, OH 45220 USA; 194 RIKEN Center for Integrative Medical Sciences, Yokohama, 230-0045 Japan; 195 University of Colorado, Denver, CO 80203 USA; 196 Center for Public Health Genomics and Department of Biostatistical Sciences, Wake Forest School of Medicine, Winston-Salem, NC 27157 USA; 197 Department of Neurology, Radiology, and Biomedical Engineering, Washington University School of Medicine, St. Louis, MO 98195 USA; 198 Department of Neurosciences, Experimental Neurology, KU Leuven – University of Leuven, Leuven, 3000 Belgium; 199 VIB Center for Brain & Disease Research, University Hospitals Leuven, Department of Neurology, Leuven, 3000 Belgium; 200 University of Lille, INSERM U1171, CHU Lille, Lille, F-59000 France; 201 Department of Medical and Molecular Genetics, King’s College London, London, WC2R 2LS UK; 202 SGDP Centre, Institute of Psychiatry, Psychology & Neuroscience, King’s College London, London, WC2R 2LS UK; 203 Cardiovascular Epidemiology Unit, Department Public Health & Primary Care, University of Cambridge, Cambridge, CB1 8RN UK; 204 Northern Institute for Cancer Research, Paul O’Gorman Building, Newcastle University, Newcastle, NE2 4AD UK; 205 Department of Clinical Sciences Lund, Neurology, Lund University, Lund, 221 00 Sweden; 206 Department of Neurology and Rehabilitation Medicine, Skåne University Hospital, Lund, 222 29 Sweden; 207 Bioinformatics Core Facility, University of Gothenburg, Gothenburg, 405 30 Sweden; 208 University of Technology Sydney, Faculty of Health, Ultimo, NSW 2007 Australia; 209 Department of Medicine, University of Maryland School of Medicine, Baltimore, MD 21201 USA; 210 Department of Neurology, Mayo Clinic, Jacksonville, FL 32224 USA; 211 Division of Geriatrics, School of Medicine, University of Mississippi Medical Center, Jackson, MS 39216 USA; 212 Memory Impairment and Neurodegenerative Dementia Center, University of Mississippi Medical Center, Jackson, FL 39216 USA; 213 Department of Epidemiology and Public Health, Graduate School of Medical Sciences, Kyushu University, Fukuoka, 819-0395 Japan; 214 Clinical Research Facility, Department of Medicine, NUI Galway, Galway, H91 TK33 Ireland; 215 Department of Neurology, Brain Center Rudolf Magnus, University Medical Center Utrecht, Utrecht, 3584 The Netherlands; 216 Centre for Clinical Brain Sciences, University of Edinburgh, Edinburgh, EH4 2XU UK; 217 Department of Neurology, University Medicine Greifswald, Greifswald, 17489 Germany; 218 Department of Medicine, Brigham and Women’s Hospital, Boston, MA 02115 USA; 219 Nuffield Department of Clinical Neurosciences, University of Oxford, Oxford, OX3 9DU UK; 220 Department of Neurology, Miller School of Medicine, University of Miami, Miami, FL 33136 USA; 221 Department of Allergy and Rheumatology, Graduate School of Medicine, the University of Tokyo, Tokyo, 13-8654 Japan; 222 Department of Pediatrics, College of Medicine, University of Oklahoma Health Sciences Center, Oklahoma City, OK 73104 USA; 223 Department of Neurology, Medical University of Graz, Graz, 8036 Austria; 224 University Medicine Greifswald, Institute for Community Medicine, SHIP-KEF, Greifswald, 17489 Germany; 225 Department of Neurology, Jagiellonian University, Krakow, 31-007 Poland; 226 University of Edinburgh, Edinburgh, EH8 9JZ UK; 227 Department of Neurology, Justus Liebig University, Giessen, 35390 Germany; 228 Department of Clinical Neurosciences/Neurology, Institute of Neuroscience and Physiology, Sahlgrenska Academy at University of Gothenburg, Gothenburg, SE-405 Sweden; 229 Sahlgrenska University Hospital, Gothenburg, SE-405 Sweden; 230 Stroke Division, Florey Institute of Neuroscience and Mental Health, Heidelberg, VIC 3084 Australia; 231 Austin Health, Department of Neurology, Heidelberg, Victoria 3084 Australia; 232 School of Medicine, Dentistry and Nursing at the University of Glasgow, Glasgow, G12 8QQ UK; 233 Department of Epidemiology and Population Health, Albert Einstein College of Medicine, Bronx, NY 10461 USA; 234 Department of Human Genetics, McGill University, Montreal, H3A 0G4 Canada; 235 Sorbonne Universités, UPMC Univ. Paris 06, INSERM, UMR_S 1166, Team Genomics & Pathophysiology of Cardiovascular Diseases, Paris, 75006 France; 236 ICAN, Institute for Cardiometabolism and Nutrition, Paris, 75013 France; 237 Department of Biomedical Engineering, University of Virginia, Charlottesville, VA 22904-4259 USA; 238 Seattle Epidemiologic Research and Information Center, VA Office of Research and Development, Seattle, WA 98108 USA; 239 Cardiovascular Research Center, Massachusetts General Hospital, Boston, MA 02114 USA; 240 Department of Medical Research, Bærum Hospital, Vestre Viken Hospital Trust, Rud, 3004 Norway; 241 Saw Swee Hock School of Public Health, National University of Singapore and National University Health System, Singapore, 119077 Singapore; 242 National Heart and Lung Institute, Imperial College London, London, SW7 2AZ UK; 243 Department of Gene Diagnostics and Therapeutics, Research Institute, National Center for Global Health and Medicine, Tokyo, 162-8655 Japan; 244 Department of Epidemiology, Tulane University School of Public Health and Tropical Medicine, New Orleans, LA 70112 USA; 245 Department of Cardiology, University Medical Center Groningen, University of Groningen, Groningen, 9700 RB Netherlands; 246 Department of Epidemiology and Biostatistics, Imperial College London, MRC-PHE Centre for Environment and Health, School of Public Health, London, W2 1PG UK; 247 Department of Cardiology, Ealing Hospital NHS Trust, Southall, HA1 3UJ UK; 248 Department of Pharmaceutical Sciences, College of Pharmacy, University of Oklahoma Health Sciences Center, Oklahoma City, OK 73104 USA; 249 Oklahoma Center for Neuroscience, Oklahoma City, OK 73104 USA; 250 Department of Pathology and Genetics, Institute of Biomedicine, The Sahlgrenska Academy at University of Gothenburg, Gothenburg, SE-405 Sweden; 251 Department of Neurology, Helsinki University Hospital, Helsinki, FI-00029 Finland; 252 Clinical Neurosciences, Neurology, University of Helsinki, Helsinki, FI-00029 Finland; 253 Department of Neurology, University of Washington, Seattle, WA 98195 USA; 254 Department of Genetics, Perelman School of Medicine, University of Pennsylvania, Philadelphia, PA 19104 USA; 255 Faculty of Medicine, University of Iceland, Reykjavik, 201 Iceland; 256 Departments of Neurology and Public Health Sciences, University of Virginia School of Medicine, Charlottesville, VA 22908 USA; 257 Center for Genomic Medicine, Kyoto University Graduate School of Medicine, Kyoto, 606-8501 Japan

## Abstract

Carotid artery intima media thickness (cIMT) and carotid plaque are measures of subclinical atherosclerosis associated with ischemic stroke and coronary heart disease (CHD). Here, we undertake meta-analyses of genome-wide association studies (GWAS) in 71,128 individuals for cIMT, and 48,434 individuals for carotid plaque traits. We identify eight novel susceptibility loci for cIMT, one independent association at the previously-identified *PINX1* locus, and one novel locus for carotid plaque. Colocalization analysis with nearby vascular expression quantitative loci (cis-eQTLs) derived from arterial wall and metabolic tissues obtained from patients with CHD identifies candidate genes at two potentially additional loci, *ADAMTS9* and *LOXL4*. LD score regression reveals significant genetic correlations between cIMT and plaque traits, and both cIMT and plaque with CHD, any stroke subtype and ischemic stroke. Our study provides insights into genes and tissue-specific regulatory mechanisms linking atherosclerosis both to its functional genomic origins and its clinical consequences in humans.

## Abstract (web summary)

Carotid intima-media thickness (cIMT) and plaque are associated with subclinical atherosclerosis and coronary heart disease (CHD). Here, the authors identify and prioritize genetic loci for cIMT and plaque by GWAS and colocalization approaches and further demonstrate genetic correlation with CHD and stroke.

## Introduction

Atherosclerosis is characterized by an accumulation of lipid-rich and inflammatory deposits (plaques) in the sub-intimal space of medium and large arteries. Plaque enlargement leads to blood flow limitation, organ ischemia, and/or tissue necrosis. Plaque rupture can lead to abrupt vascular occlusion, which underlies clinical cardiovascular events, including myocardial infarction and ischemic stroke. Coronary heart disease (CHD) accounts for one in seven deaths, and stroke accounts for one in 20 deaths in the US^1^. Because atherosclerosis has a long pre-clinical phase, early detection of atherosclerosis using non-invasive methods may help identify individuals at risk for atherosclerotic clinical events^2^, and provides an opportunity for prevention. Subclinical atherosclerosis can be detected by B-mode ultrasound measurement of common carotid artery intima-media thickness (cIMT) or carotid plaques^1^.

Subclinical and clinical atherosclerosis has known genetic components^3^. Genome-wide association studies (GWAS) of subclinical atherosclerosis have previously identified three loci significantly associated with cIMT at *ZHX2*, *APOC1*, and *PINX1*, and two loci associated with common carotid artery plaque at *PIK3CG* and *EDNRA*^4^. An exome-wide-association study identified significant associations of the *APOE* ε2 allele with cIMT and coronary artery calcification^5^. The *APOE* single nucleotide polymorphism (SNP) rs7412 is in linkage disequilibrium (LD) with the *APOC1* variant, thus representing the same signal. Additional GWAS-identified associations were reported for carotid plaque at the 9p21 and *SFXN2* loci^6^, and for cIMT at the *CFDP1*-*TMEM170A* locus^7^. However, these prior studies were of limited sample size and genomic coverage, and failed to investigate the etiological role that subclinical atherosclerosis may have on atherosclerotic clinical events.

Herein, we perform a large meta-analysis of GWAS of subclinical atherosclerosis by analyzing 1000 Genomes imputed genotype data obtained from collaborations between the Cohorts for Heart and Aging Research in Genomic Epidemiology (CHARGE) consortium^8^ and the University College London-Edinburgh-Bristol (UCLEB) consortium^9^. One of the greatest challenges in the translation of GWAS findings to biological understanding is related to the limited access to RNA expression data from disease-relevant tissues. Consequently, we sought to reliably identify the tissue-specific gene regulatory functions responsible for the GWAS signals by prioritizing candidate genes for established and novel loci of cIMT and carotid plaque using statistical methods for colocalization^10^. These methods integrate identified loci with expression quantitative loci (eQTLs) inferred from cardiovascular disease-relevant genetics of RNA expression, the Stockholm-Tartu Atherosclerosis Reverse Network Engineering Task (STARNET) study, where arterial wall and metabolic-related RNA samples were collected from up to 600 patients with CHD^11^. We also evaluate the relationships of cIMT and carotid plaque with clinically apparent CHD and stroke using summary data from two large consortia. In summary, our study sequentially assesses the genetic epidemiology and tissue-specific patterns of gene regulation involved in the formation of subclinical atherosclerosis traits across cardiovascular disease-related tissues.

## Results

### Study description

**Fig. 1:**
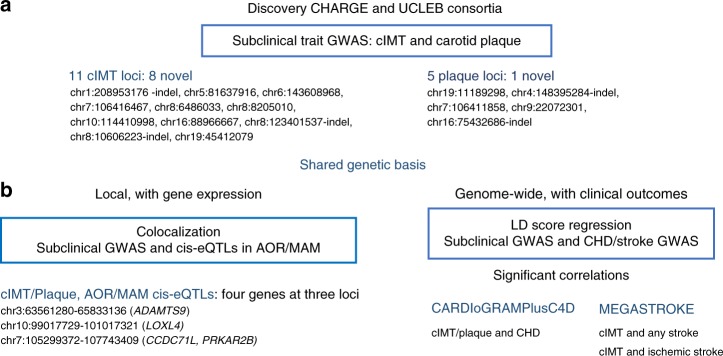
Overall study design. **a** GWAS meta-analyses of cIMT and carotid plaque for gene discovery. **b** Local and genome-wide shared genetic basis using gene expression and clinical outcomes GWAS data

The study design is shown in Fig. 1. We undertook meta-analysis of GWAS in individuals of European ancestry for cIMT (up to 71,128 participants from 31 studies) and carotid plaque (up to 48,434 participants from 17 studies; 21,540 with defined carotid plaque) (Supplementary Table 1). cIMT and plaque were evaluated using high-resolution B-mode ultrasonography and reading protocols as previously reported^4^. Carotid plaque was defined by atherosclerotic thickening of the common carotid artery wall or the proxy measure of luminal stenosis greater than 25% (Supplementary Table 2). Each cohort performed association analyses using standardized protocols (Methods) for variants imputed based on the 1000 Genomes Project (1000G) phase 1 v3 reference. Extensive quality control (QC) was applied to data, and there was little evidence for population stratification in any of the studies for either trait (Supplementary Table 3). The study-specific results were combined using fixed-effect meta-analyses, given the low heterogeneity across studies (0% heterogeneity)^12^.

### GWAS meta-analyses of cIMT and carotid plaque

For cIMT, 11 loci had at least one SNP association that reached the genome-wide association threshold (*p* < 5 × 10^−8^), of which eight were newly described and three have been previously reported (Table 1). The closest genes for the eight loci were: 1q32.2 intergenic (rs201648240), *ATP6AP1L* (rs224904), *AIG1* (rs6907215), *PIK3CG* (rs13225723), *MCPH1* (rs2912063), *SGK223* (rs11785239), *VTI1* (rs1196033), and *CBFA2T3* (rs844396). For three loci previously reported, the closest genes were *ZHX2* (rs148147734), *PINX1* (rs200482500), and *APOE* (rs7412).

**Table 1:** Loci significantly associated with cIMT and plaque GWAS

SNP	Chr:position	Nearest coding gene	Alleles (effect/other)	Effect allele freq.	Beta (SE)	*p*	*N*
Newly identified loci for cIMT							
rs201648240	1:208953176-indel	*LINC01717*	−/AA	0.83	−0.0062 (0.0011)	4 × 10^−9^	54,752
rs224904	5:81637916	*ATP6AP1L*	C/G	0.95	−0.0088 (0.0016)	5 × 10^−8^	68,962
rs6907215	6:143608968	*AIG1*	T/C	0.60	−0.0040 (0.0007)	5 × 10^−8^	64,586
rs13225723	7:106416467	*PIK3CG*	A/G	0.22	0.0052 (0.0009)	3 × 10^−9^	68,070
rs2912063	8:6486033	*MCPH1*	A/G	0.71	0.0045 (0.0008)	9 × 10^−9^	67,401
rs11785239	8:8205010	*SGK223*	T/C	0.65	−0.0043 (0.0008)	9 × 10^−9^	67,107
rs11196033	10:114410998	*VTI1A*	A/C	0.48	0.0042 (0.0008)	4 × 10^−8^	57,995
rs844396	16:88966667	*CBFA2T3*	T/C	0.30	−0.0051 (0.0009)	6 × 10^−9^	50,377
Newly identified loci for plaque							
rs200495339	19:11189298-indel	*LDLR*	−/G	0.11	−0.1023 (0.0179)	1 × 10^−8^	36,569
Known loci for cIMT							
rs148147734^a^	8:123401537-indel	*ZHX2*	−/G	0.54	0.0050 (0.0007)	3 × 10^−11^	58,141
rs200482500^a^	8:10606223-indel	*PINX1*	−/GTACC	0.52	0.0056 (0.0008)	7 × 10^−12^	58,141
rs7412^a^	19:45412079	*APOE*	T/C	0.08	−0.0119 (0.0015)	1 × 10^−14^	44,607
Known loci for plaque							
rs11413744^b^	4:148395284-indel	*EDNRA*	−/T	0.86	−0.1586 (0.0253)	4 × 10^−10^	39,577
rs17477177^b^	7:106411858	*PIK3CG*	T/C	0.79	−0.1305 (0.0197)	4 × 10^−11^	47,863
rs9632884^b^	9:22072301	9p21	C/G	0.48	0.1127 (0.0163)	5 × 10^−12^	45,943
rs113309773^b^	16:75432686-indel	*CFDP1- TMEM170A*	−/C	0.46	−0.1259 (0.0194)	9 × 10^−11^	37,104

*p* = *p*-values of association from linear regression analysis, *N* = total number in meta-analyses

^a^Published cIMT SNP in LD with our most significant SNP: rs11781551 (*r*^2^ = 0.95 with rs148147734), rs6601530 (*r*^2^ = 0 with rs200482500), and rs445925 (*r*^2^ = 0.60 with rs7412)

^b^Published plaque SNP in LD with our most significant SNP: rs1878406 (*r*^2^ = 0.98 with rs11413744), rs17398575 (*r*^2^ = 0.8 with rs17477177), rs9644862 (*r*^2^ = 0.79 with rs9632884), and rs4888378 (*r*^2^ = 0.94 with rs113309773)

The *PIK3CG* is a newly described locus for cIMT, but has been previously reported in a GWAS of carotid plaque^4^. The two signals on chromosome 8 near *MCPH1* (rs2912063) and *SGK223* (rs11785239) were confirmed to be independent through conditional analysis (Supplementary Table 4). At the *PINX1* locus, the lowest association *p*-value variant (rs200482500) was not in LD with the previously reported associated variant in the region (rs6601530, *r*^2^ = 0.0, Table 1), thus representing an independent signal at this locus. Two additional loci for cIMT had an SNP that reached suggestive evidence for association (*p* < 1.0 × 10^−7^) including an SNP nearby *APOB* (rs515135) and an intronic low frequency variant at *ATG4B* (rs139302128, minor allele frequency [MAF] = 0.03) (Supplementary Table 5).

The GWAS meta-analysis for carotid plaque identified five loci, of which one has not been previously described (nearby gene *LDLR*) (Table 1). At four known loci associated with carotid plaque (nearby genes *EDNRA*, *PIK3CG*, *CFDP1-TMEM170A*, and at the 9p21 region), the most significantly associated variants were in LD with the previously reported SNPs (Table 1)^4,6,7^, indicating that these SNPs mark the same association at each locus. Two suggestive loci (*p* < 10^−7^) were also identified nearby the genes *TMCO5B* and *STEAP2-AS1* (Supplementary Table 5). Conditional analyses confirmed the presence of a single independent signal at each locus. Manhattan and QQ plots from the meta-analysis of cIMT and carotid plaque are shown in Supplementary Figure 1 and regional plots in Supplementary Figure 2. Forest Plots for all loci are shown in Supplementary Figure 3.

### Regulatory annotations of GWAS SNPs for cIMT/carotid plaque

To better define potentially causal variants within the identified genetic risk loci, we jointly analyzed the GWAS data with functional genomic information such as annotations on active transcription sites or open chromatin regions (i.e., performed a fine-mapping functional genome-wide association analysis using fGWAS^13^). Only variants in the *PINX1* region were found to have a high probability that its association with cIMT is driven by SNPs that fall within transcription sites in adipose-derived mesenchymal stem cells at a DNaseI-hypersensitive site (Supplementary Figure 4), a finding that provides a down-stream mechanistic explanation for the cIMT signal in the *PINX1* locus.

**Fig. 2:**
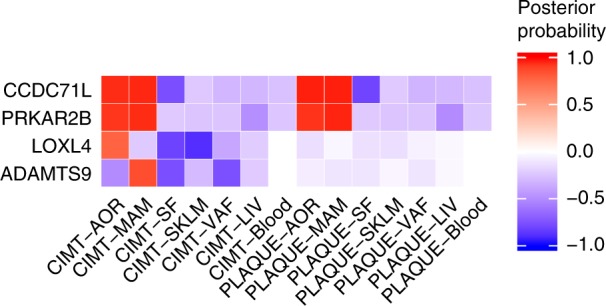
Pairwise colocalization results for genes identified for cIMT and carotid plaque GWAS meta-analysis with STARNET expression datasets. Red indicates a high posterior probability of colocalization and blue a high probability of no colocalization of the same SNP with tissue eQTLs

To further explore the regulatory functions of variants in the identified loci for cIMT and carotid plaque, we investigated whether the identified lead SNPs were also eQTLs using vascular RNAseq data from GTEx (aorta, coronary and tibial arteries, heart atrial appendage, and heart left ventricle) and from the coronary artery disease cohort of STARNET (i.e., from the atherosclerotic-lesion-free internal mammary artery [MAM] and atherosclerotic aortic root [AOR]). Lead SNP associated with cIMT and carotid plaque (rs13225723) in the *PIK3CG* locus was found to be vascular-specific eQTLs for *CCDC71L* and *PRKAR2B* in GTEx aorta as well as in STARNET AOR and MAM tissues (Table 2, Fig. 2), suggesting that the genetic regulation of these two genes are responsible for risk variation in cIMT and carotid plaque development in this locus.

**Table 2:** Gene expression results for significant SNPs in GTEx and STARNET tissues

SNP	eQTL^a^ (Gene, *p*) GTEx		eQTL^a^ (Gene, *p*) STARNET tissues	
	AOR^b^	HEART (ATR/VEN)^c^	AOR	MAM
rs201648240	*CAMK1G*, 0.0094 *AL031316.1*, 0.0040			*CD34*,0.00532 *TRAF3IP3*, 0.0097
rs6907215		*AL023584.1*, 0.005384704 (VEN)	*ENSG00000217648*, 0.00046	*ENSG00000217648*, 0.8 × 10^−5^
rs13225723	*AC005050.1*, 1 × 10^−10^ *ENSG00000177820.5*, 7.0 × 10^−5^ *CCDC71L*, 5 × 10^−6^ *PRKAR2B*, 4 × 10^−8^ *PIK3CG,* 10 × 10^−3^		*CCDC71L*, 6 × 10^−36^ *PRKAR2B*, 7 × 10^−7^ *SYPL1*, 0.0043	*CCDC71L*, 3 × 10^−33^ *PRKAR2B*, 6 × 10^−8^ *NAMPT*, 6 × 10^−6^
rs2912063	*MCPH1*, 0.0041	*ENSG00000271743.1*, 0.0093 (VEN)	*MCPH1-AS1*, 0.0020	
rs11785239		*AC022784.1*, 0.0078 (VEN)	*ERI1*, 0.0069	*PPP1R3B*, 0.0036
rs844396	*ENSG00000141012.8*, 0.003 A*C092384.2*, 0.001 *CBFA2T3*, 1 × 10^−7^	*ZNF469*, 0.004 (ATR) *AC092384.3*, 5 × 10^−6^ (ATR) *AC092384.1*, 0.002 (ATR) *CBFA2T3*, 0.0004 (ATR) *ZNF469*, 0.002 (VEN) *AC138028.4*, 0.001 (VEN) *ENSG00000224888.3*, 0.009(VEN) *PIEZO1*, 0.0004 (VEN) *GALNS*, 0.004 (VEN)	*RPL13*, 0.0024 *ZNF276*, 0.0070 *TRAPPC2L*, 0.0091	*TRAPPC2L*, 0.0040 *ZNF276*, 0.0059
rs200495339		*ENSG00000267105.1*, 0.0005 (VEN)		
rs148147734	*DERL1*, 0.0082			
rs200482500	*AF131215.6*, 0.005 *AF131215.5*, 0.001	*AF131215.5*, 0.002 (ATR) *AF131215.6*, 0.003 (VEN) *AF131215.5*, 0.004 (VEN)		
rs7412	ENSG00000267163.1, 0.007			
rs11413744	*PRMT9*, 0.004			
rs17477177	*ENSG00000267052.1*, 6 × 10^−11^ *ENSG00000177820.5*, 5 × 10^−6^ *CCDC71L*, 4 × 10^−7^ *PRKAR2B*, 2 × 10^−8^	*BCAP29*, 0.002 (ATR)	*CCDC71L*, 2 × 10^−37^ *PRKAR2B*, 6 × 10^−7^ *SYPL1*, 0.0091	*CCDC71L*,1 × 10^−33^ *PRKAR2B*, 2 × 10^−8^ *NAMPT*, 1 × 10^−5^
rs9632884		*DMRTA1*, 0.007 (ATR)	*CDKN2B*, 2 × 10^−3^	*CDKN2B*, 2 × 10^−3^
rs113309773	*BCAR1*, 6 × 10^−11^ *ENSG00000261783.1*, 2 × 10^−16^ *GABARAPL2*, 0.004	*ENSG00000261783.1*, 1 × 10^−5^ (ATR) *ENSG00000166822.8,* 0.005 *(ATR)* *ENSG00000261783.1*, 0.0003 (VEN)	*ZFP1*, 4 × 10^−4^ AC009078.2, 0.002 *BCAR1*, 3 × 10^−12^ *CFDP1*, 0.002 *TMEM170A*, 0.009	

*p* = *p*-values of association from linear regression analysis

^a^The lead SNP from GWAS is considered an eQTL if the *cis*-association has a nominal *p*-value of association <0.01. Multiple but not all lead SNPs reach genome-wide significance (*p* < 10^−4^).

^b^This includes aorta (AOR)

^c^This includes heart atrial (ATR) and heart left ventricle (VEN)

### Colocalization analysis of GWAS data and STARNET eQTLs

To identify further candidate genes in tissues affected by atherosclerosis that had strong evidence of sharing the same variant for cIMT and carotid plaque as found in our GWAS, we conducted pairwise colocalization analysis of these genetic variants with *cis*-eQTLs in the STARNET study^10^.

**Fig. 3:**
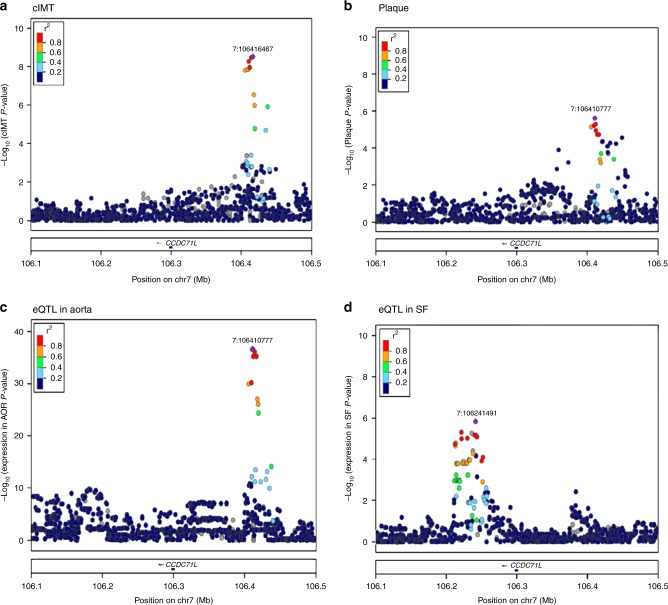
Association results at the *CCDC71L* locus (chromosome 7), showing a high posterior probability of a shared variant for cIMT and carotid plaque in AOR and MAM eQTLs. −log10(*p*) SNP association *p*-values for cIMT (plot A) and carotid plaque (plot B), and eQTL in AOR (plot C) and eQTL in SF (plot D). Association results in SF tissue have a low probability of a shared signal with cIMT and carotid plaque, possibly indicating a different mechanism in this tissue. eQTLs in MAM are identical to AOR and not shown. The *p*-values were calculated by fitting a linear regression model with cIMT or plaque as dependent variable and imputed SNPs as independent variables. Each dot is an SNP and the color indicates linkage disequilibrium (*r*^2^) with the best hit (in purple)

The pairwise colocalization analysis is based on coloc, a Bayesian statistical methodology that tests pairwise colocalization of SNPs in GWAS with eQTLs and, in this fashion, generates posterior probabilities for each locus weighting the evidence for competing hypothesis of either no colocalization or sharing of a distinct SNP at each locus^10^. We used summary statistics from all SNPs within a 200-kb window around each gene covered by the eQTL datasets (*N* = 18,705, see Methods), and analyzed each eQTL-GWAS dataset pair (Supplementary Table 6). A posterior probability of ≥75% was considered strong evidence of the tissue-specific eQTL-GWAS pair influencing both the expression and GWAS trait at a particular region. Results for this analysis are shown in Table 3 and Supplementary Figure 5. The strongest evidence for an effect on gene expression within the regions identified in our standard GWAS meta-analysis was for the *CCDC71L* and *PRKAR2B* genes at the previously described chromosome 7 cIMT locus (*PIK3CG* in Table 2, Fig. 2). These genes showed evidence of colocalization for both cIMT and carotid plaque in AOR and MAM tissues (Table 3, Fig. 3). *CCDC71L* had the highest probability (>95%) for colocalization for cIMT, and MAM and AOR tissue eQTLs, and for carotid plaque, and MAM and AOR tissue eQTLs. We found a low probability of colocalization of the SNP with the *PIK3CG* gene expression (<1%).

**Table 3:** Colocalization of cIMT and plaque with eQTLs in tissues from patients with CHD in STARNET tissues for genes/tissues combinations that have more than 75% probability to share the same associated variant

Region (chr:start-stop)	Trait	Gene	SNP with best joint probability	*p*, BETA (SE), Tissue posterior probability (PPA)^a^			Direction of effect GWAS/eQTL
				cIMT /plaque GWAS	AOR eQTL	MAM eQTL	
chr3:63561280-65833136	cIMT	*ADAMTS9*	rs17676309 (T/C)	2 × 10^−6^, -0.0035 (0.0007)	2 × 10^−25^, −0.65 (0.06) PPA=0.93	1 × 10^−23^, −0.61 (0.06) PPA=0.89	−/−
chr10:99017729-101017321	cIMT	*LOXL4*	rs55917128 (T/C)	5 × 10^−7^, 0.0037 (0.0007)	6 × 10^−8^, 0.33 (0.06) PPA=0.79		+/+
chr7:105299372-107743409	cIMT	*CCDC71L* *PRKAR2B*	rs12705390 (A/G)	5 × 10^−9^, 0.0049 (0.0008)	2 × 10^−37^, 0.81 (0.06) PPA=0.97 6 × 10^−7^, 0.34 (0.07) PPA=0.93	1 × 10^−33^, 0.755 (0.06) PPA=0.97 2 × 10^−8^, 0.368 (0.06) PPA=0.96	+/+ +/+
	Plaque	*CCDC71L* *PRKAR2B*	rs12705390 (A/G)	4 × 10^−8^, 0.12 (0.022)	2 × 10^−37^, 0.80 (0.06) PPA=0.97 6 × 10^−7^, 0.33 (0.07) PPA=0.93	1 × 10^−33^, 0.75 (0.06) PPA=0.97 2 × 10^−8^, 0.37 (0.06) PPA=0.96	+/+ +/+

*PPA* posterior probability of sharing same SNP higher than 75%, *cIMT* common carotid artery intima-media thickness, *AOR* aorta, *MAM* mammary artery

^a^This signal reaches genome-wide significance in cIMT/plaque, and reaches a high probability of being mediated by the genes in AOR and MAM

The eQTL associations at two additional loci (*ADAMTS9*, *LOXL4*) in MAM or AOR showed evidence of colocalization with cIMT or carotid plaque, although GWAS association *p*-values at these loci did not meet the genome-wide significance threshold (Table 3, Supplementary Figure 5). Albeit with weaker magnitudes, the expression of these two genes were also associated with the top colocalizing SNPs as detected in RNAseq data in GTEx aorta (rs17676309, chr3:64730121, *ADAMTS9*, *p* = 0.0003 and rs55917128, chr10:100023359, *LOXL4*, *p* = 0.0005).

### Colocalization of CHD and stroke GWAS and STARNET eQTLs

We next assessed if the four genes (*CCDC71L, PRKAR2B, ADAMTS9, LOXL4)* identified through colocalization of cIMT/carotid plaque with tissue-specific eQTLs also showed evidence for colocalization with CHD and stroke traits (Supplementary Data 1 and Supplementary Figure 6). We used GWAS summary data for CHD (CARDIoGRAMPlusC4D), and stroke subtypes (MEGASTROKE) and AOR and MAM STARNET tissue eQTLs for these analyses. *CCDC71L* and *PRKAR2B* had suggestive evidence of sharing the same variant with large vessel disease stroke in both AOR and MAM tissues (probability of colocalization ≥20%, Supplementary Data 1). In contrast, there was strong evidence (≥75%) to reject a shared variant for CHD and eQTLs at this locus, thus suggesting there is atherosclerotic outcome specificity at vascular level for this locus (Supplementary Figure 5). Three of these genes, *CCDC71L*, *PRKAR2B*, and *ADAMTS9*, showed evidence for shared genetic influences of cIMT or carotid plaque on CHD/stroke outcomes when testing the joint association using moloc, a multiple-trait extension of coloc^14^ (Supplementary Table 7). We also highlight the expression of *KIAA1462* gene in MAM, carotid plaque/cIMT, and CHD, which were positively correlated (Supplementary Figure 7). This gene has suggestive evidence of pairwise colocalization with carotid plaque (67% of probability of shared variant between carotid plaque and eQTL in MAM), as well as a high probability of shared variant between MAM eQTL expression of this gene, GWAS carotid plaque or cIMT, and CHD traits (Supplementary Table 7). We note, however, that the GWAS signal for outcomes across the datasets did not reach genome-wide significance and larger sample sizes may be needed to strengthen the evidence for involvement in disease outcomes.

### Genetic correlations of cIMT/carotid plaque and clinical outcomes

To provide etiological insights into the role of measures of subclinical atherosclerosis and major atherosclerotic disease outcomes such as CHD and ischemic stroke, we quantified the genetic correlation using cross-trait LD score regression, a method that estimates genetic correlation across different traits using summary level data^15^. We used summary statistics between cIMT/carotid plaque with CHD and stroke meta-analysis of GWAS. Both cIMT and carotid plaque had positive significant genetic correlations with CHD (all *p* < 0.05 after adjusting for multiple testing), though the magnitude of the correlation was twice as strong for carotid plaque (0.52) as for cIMT (0.20) (Table 4). There was also evidence for genetic correlations between cIMT with any stroke and ischemic stroke subtype.

**Table 4:** Genetic correlation between CHD and stroke traits with cIMT and plaque, and cIMT with plaque using LD score and meta-GWAS

Cardiovascular disease trait	Subclinical atherosclerosis trait	Genetic correlation	SE	*z*	*p*
CHD^a^	cIMT	0.20	0.05	4.1114	4 × 10^−5^
Any stroke	cIMT	0.30	0.07	4.2301	2.3 × 10^−5^
Ischemic stroke^b^	cIMT	0.31	0.07	4.646	3.4 × 10^−6^
Cardio-embolic stroke^b^	cIMT	0.10	0.09	1.0729	0.28
Small vessel disease stroke^b^	cIMT	0.33	0.18	1.8728	0.06
CHD^a^	Carotid plaque	0.52	0.08	6.4263	1.3 × 10^−10^
Any stroke^b^	Carotid plaque	0.28	0.10	2.7097	0.007
Ischemic stroke^b^	Carotid plaque	0.27	0.10	2.6578	0.008
Cardio-embolic stroke^b^	Carotid plaque	0.06	0.14	0.4684	0.64
Small vessel disease stroke^b^	Carotid plaque	−0.03	0.24	−0.1344	0.89
Plaque	cIMT	0.40	0.10	3.9667	7.3 × 10^−5^

^a^CARDIoGRAMPlusC4D

^b^MEGASTROKE consortium. Unable to estimate the genetic correlations with large vessel disease

### Pathway analysis and druggability

Gene Ontology (GO) analyses of genes identified in the loci for cIMT and carotid plaque according to our meta-analysis of GWAS (Table 1 and Supplementary Table 5) and in the colocalization analyses (Table 3, Supplementary Table 7) showed that cIMT genes are enriched in lipoprotein-related terms and cholesterol efflux, whereas carotid plaque genes are enriched in terms associated with fibroblast apoptosis (Supplementary Figure 8). Analysis of the cIMT genes using a GO Slim additionally identified several of the genes that were associated with terms describing cardiovascular development, cell adhesion, and immune processes, processes already considered relevant to atherosclerosis. Specifically, there is corroborating evidence from GO that *CCDC71L, PRKAR2B,* and *TWIST1* are associated with cIMT/carotid plaque as they are involved in lipid metabolism, with similar support that *ADAMTS9, CDH13,* and *KIAA1462* are associated with cIMT or carotid plaque risk as they are all involved in cell adhesion and, together with *TWIST1*, in cardiovascular system development (Supplementary Data 2).

From the loci associated with cIMT and carotid plaque, we identified seven genes (*ATG4B, ALPL, LDLR, APOB, EDNRA, APOE*, and *ADAMTS9*) whose encoded proteins are targets at various stages of the drug development process (Supplementary Tables 8 and 9). *ADAMTS9* gene encodes a protein likely to be druggable^16^. *ATG4B, ALPL,* and *LDLR* are proteins being targeted by compounds in pre-clinical phase (tier 2), while *APOB* and *EDNRA* are proteins targeted by drugs in clinical phase or licensed (tier 1). *APOB* is the target of an approved FDA drug for treatment of familial hypercholesterolemia. *EDNRA* gene encodes for endothelin A receptor, against which several antagonists have been developed for the treatment of pulmonary arterial hypertension or which are in advanced clinical phase development for non-small cell lung cancer and diabetic nephropathy.

## Discussion

We provide results of a large meta-analysis of GWAS of subclinical atherosclerosis and we integrate our results with tissue-specific gene expression data using eQTLs from both the early (MAM) and late advanced (AOR) atherosclerotic arterial wall from the STARNET study to enable reliable discovery of genes with biological evidence of an increased probability for conferring inherited risk of atherosclerosis development. Our discovery approach using GWAS meta-analyses identified 16 loci significantly associated with either cIMT or carotid plaque, of which nine are novel.

The integration of GWAS and tissue-specific *cis*-eQTLs for the joint analyses of tissue-specific eQTLs from CHD patients identified two potentially additional loci colocalizing with cIMT or carotid plaque: chr3:63561280-65833136 (*ADAMTS9*), chr10:99017729-101017321 (*LOXL4). ADAMTS9* is a metalloproteinase involved in thrombosis and angiogenesis and has been associated with cardiometabolic traits (waist-to-hip ratio, waist circumference, and type 2 diabetes) in GWAS, and with coronary artery calcification in a gene-by-smoking interaction GWAS^17,18^. *LOXL4* encodes a lysyl oxidase involved in crosslinks of collagen and elastin in the extracellular matrix. This family of proteins are involved in the development of elastic vessels and mechanical strength of the vessel wall, and their inhibition was associated with the development of abdominal aortic aneurysms and more severe atherosclerosis in experimental models^19^.

Some loci identified in our meta-analysis of GWAS include genes in known pathways for atherosclerosis, including *LDLR*, which is related to lipid pathways and CHD, and identified for associations with carotid plaque in our study. For most of the loci, however, the underlying gene implicated in signals are unknown. Our colocalization approach found both *CCDC71L* and *PRKAR2B* as the most likely genes at the chromosome 7 locus, where *PIK3CG* was previously the suggested gene. This finding is in agreement with a targeted sequencing study of subclinical atherosclerosis^15^. An additional SNP (rs342286) at this locus has been associated with platelets volume and reactivity, and cardiovascular traits. However, rs342286 is not in LD with our most significant SNP and it is not associated with cIMT or carotid plaque in our studies (*p* = 0.49 and 0.01, respectively). Of interest, the variant we identified in this study showed evidence for colocalization with cIMT/carotid plaque and large vessel disease stroke but not CHD, therefore showing tissue and outcome-specificity. *CCDC71L* has unknown function. *PRKAR2B* codes for one of the several regulatory subunits of cAMP-dependent protein kinase and its expression is ubiquitous. In vitro studies have shown that adenosine-induced apoptosis of arterial smooth muscle cells involves a cAMP-dependent pathway^20^.

Measures of cIMT and carotid plaque reflect vascular pathophysiologic and atherosclerosis processes, respectively, with carotid plaque more strongly reflecting atherosclerotic clinical events. An important contribution of this study is the supporting evidence for overall genetic correlations of CHD and stroke (any cause and ischemic stroke) with subclinical atherosclerosis traits, estimated using LD score methods. Further highlighting the potential biological relevance of our findings, the genetic correlations estimates for CHD were stronger for carotid plaque than for cIMT. However, cIMT and carotid plaque GWAS were correlated, and the genetic correlations estimates with stroke were similar for cIMT or carotid plaque, and not significant for carotid plaque. The colocalization analyses provided additional insights in the relationships between subclinical atherosclerosis, clinical outcomes, and tissue-specific regulation at specific genomic regions. For example, our suggestive top gene association in multi-trait colocalization for *KIAA1462* included MAM eQTLs, carotid plaque, and CHD, supporting the shared genetic effects at this locus of atherosclerosis in carotid and coronary arteries. *KIAA1462* has been previously reported in the same locus identified by GWAS for CHD^21^. This gene encodes a protein involved in cell–cell junctions in endothelial cells^22^, which was recently shown to be involved in pathologic angiogenic process in in vitro and in vivo experimental models^23^. These findings suggest that there may be important differences in vascular bed regulation at distinctive regions for atherosclerotic cardiovascular and stroke outcomes that may help to identify genes and specific targets for CHD or stroke prevention and treatment.

Additional studies in diverse and large samples across the multiple datasets are needed to explore these results further. As more summary statistics become available for other clinical end-points beyond stroke and CHD (both in terms of larger sample size and richer genome coverage), and as further refinements in clinical phenotypes emerge (e.g. from CHD to acute coronary syndrome sub-components), strategies to integrate this knowledge using methods such as *moloc*^10^ and *eCAVIAR*^24^ will continue to be essential for harnessing genome-wide findings in the drug-discovery process.

In summary, our study is a large GWAS meta-analysis of cIMT and carotid plaque. Through a sequential approach of discovery and colocalization studies, we provide deeper insights into disease causal genes of subclinical cIMT and carotid plaque formation. We confirmed three loci and identified nine novel loci in the meta-analyses of cIMT and carotid plaque. Additionally, we provide strong evidence for the role of three novel genes from our integrative analysis of GWAS and eQTL data. Moreover, the identified correlations with CHD and stroke highlight novel biological pathways that merit further assessments as novel targets for drug development.

## Methods

### Ethics statement

All human research was approved by the relevant institutional review boards for each study, and conducted according to the Declaration of Helsinki. All participants provided written informed consent.

### Populations and phenotypes

The discovery GWAS in this study consists of a collaboration between the CHARGE ^8^ and the UCLEB consortia^9^, for genetic studies of cIMT and carotid plaque among individuals of European ancestry (Supplementary Note 1). All studies followed standardized protocols for phenotype ascertainment and statistical analyses. The descriptive characteristics of participating studies are shown in Supplementary Table 1.

cIMT and carotid plaque measures were evaluated using high-resolution B-mode ultrasonography and reading protocols as previously reported^4^. We used data from the baseline examination or the first examination in which carotid ultrasonography was obtained. cIMT was defined by the mean of the maximum of several common carotid artery measurements, measured at the far wall or the near wall. For most studies, this was an average of multiple measurements from both the left and right arteries. We also examined a carotid plaque phenotype, defined by atherosclerotic thickening of the carotid artery wall or the proxy measure of luminal stenosis greater than 25% (Supplementary Table 2).

### Genotyping, imputation, and study-level quality control

Genotyping arrays and QC pre-imputation are shown in Supplementary Table 3. Each GWAS study conducted genome-wide imputation using a Phase 1 integrated (March 2012 release) reference panel from the 1000G Consortium using IMPUTE2^25^ or MaCH/minimac^26^, and used Human Reference Genome Build 37. Sample QC was performed with exclusions based on call rates, extreme heterozygosity, sex discordance, cryptic relatedness, and outlying ethnicity. SNP QC excluded variants based on call rates across samples and extreme deviation from Hardy–Weinberg equilibrium (Supplementary Table 3). Non-autosomal SNPs were excluded from imputation and association analysis.

Pre-meta-analysis GWAS study-level QC was performed using EasyQC software^27^. This QC excluded markers absent in the 1000G reference panel; non A/C/G/T/D/I markers; duplicate markers with low call rate; monomorphic SNPs and those with missing values in alleles, allele frequency, and beta estimates; SNPs with large effect estimates or standard error (SE) ≥10; and SNPs with allele frequency difference >0.3 compared to 1000G reference panel. There was a total of 9,574,088 SNPs for the cIMT meta-analysis and 8,578,107 SNPs for the carotid plaque meta-analysis.

### Statistical analyses

Within each study, we used linear and logistic regression to model cIMT and carotid plaque, respectively, and an additive genetic model (SNP dosage) adjusted for age, sex, and up to 10 principal components. We combined summary estimates from each study and each trait using an inverse variance weighted meta-analysis. Additional filters were applied during meta-analyses including imputation quality (MACH *r*^2^ < 0.3 and IMPUTE info <0.4), a minor allele frequency (MAF) <0.01, and SNPs that were not present in at least four studies. The genome-wide significance threshold was considered at *p* < 5.0 × 10^−8^.

To assess the evidence for independent associations at each locus attaining genome-wide significance, we performed conditional analysis in a 1-Mb genomic interval flanking the lead SNP using GCTA^28^. This approach uses summary meta-analysis statistics and a LD matrix from an ancestry-matched sample to perform approximate conditional SNP association analysis. The estimated LD matrix was based on 9713 unrelated individuals of European ancestry from the ARIC study, which was genotyped using an Affymetrix 6.0 array and imputed to the 1000G panel using IMPUTE2^25^.

### Gene expression analysis using GTEx

GTEx Analysis V6 (dbGaP Accession phs000424.v6.p1) eQTL results were downloaded from GTEx portal for 44 tissues, and then mapped to SNPs listed in Table 1. We used a false discovery rate (FDR) of ≤0.05.

### Colocalization analyses using eQTLs

We integrated our GWAS results with *cis-*eQTL data using a Bayesian method (coloc)^10^. This method evaluates whether the GWAS and eQTL associations best fit a model in which the associations are due to a single shared variant (summarized by the posterior probability). We used gene expression datasets from multiple tissues from patients with CHD of the STARNET study, including blood, MAM, AOR, subcutaneous fat (SF), visceral fat (VAF), skeletal muscle (SKLM), and liver (LIV) obtained from 600 patients during open heart surgery^11^. Pairwise colocalization was tested between these expression disease tissue datasets and GWAS results from our cIMT/carotid plaque GWAS meta-analysis. We used GWAS and eQTL summary statistics of SNPs within a 200-kb window around each gene covered by the eQTL datasets. A posterior probability of colocalization ≥0.75 was considered a strong evidence for a causal gene. Next, we reported the gene(s) in the STARNET datasets that had the strongest evidence of sharing the same variant with cIMT or carotid plaque genome-wide. In an alternative analysis, we also tested loci with an SNP that reached a threshold of significant or suggestive genome-wide significance for cIMT or carotid plaque (reported in Table 1, Supplementary Table 5). For each region 200kb around the SNP with the lowest association *p*-value, we report the gene with the highest probability of being responsible for the GWAS signal (Supplementary Table 6).

Pairwise colocalization for these genes was also tested for publicly available GWAS for CHD case-controls (CARDIoGRAMPlusC4D) and stroke case-controls (MEGASTROKE consortium). The MEGASTROKE dataset uses genotypes imputed to the 1000G phase I haplotype panel. The European ancestry sample used to generate these results consisted of 40,585 stroke cases and 406,111 controls from 15 cohorts and two consortia: the METASTROKE and CHARGE consortia^29^. The phenotypes used in this analysis were any stroke (*n* = 39,067 cases, total *n* = 442,142), ischemic stroke (IS, *n* = 32,686 cases, total *n* = 423,266), and etiologic stroke subtypes:cardioembolic stroke (CE, *n* = 6,820 cases, total *n* = 314,368), large vessel disease (*n* = 4,113, total *n* = 202,263), and small vessel disease (SVD, *n* = 4,975, total *n* = 242,250). To explore multi-trait colocalizations, we used moloc^14^ with prior probabilities of 10^−4^ for GWAS/GWAS/eQTL, 10^−6^ for GWAS+eQTL/GWAS or GWAS+GWAS/eQTL, and 10^−7^ for colocalization of all three association signals.

### Functional annotation and epigenetic enrichment analyses

From the Epigenome Roadmap Project^30,31^, we obtained regulatory information using broad classes of chromatin states (*n* = 127 tissues) capturing promoter-associated, transcription-associated, active intergenic, and large-scale repressed and repeat-associated states. From ENCODE^32^, we obtained chromatin states, uniformly processed transcription factor (TF) Chip assays and DNaseI Hypersensitivity sites (DHS) for nine cells lines. From FANTOM5^33^, we used information from expression of enhancers in each tissue (*n* = 112), and enhancers that are positively differentially expressed against any other tissue (*n* = 110).

We used fGWAS^13^ to identify genomic annotations that are enriched within the cIMT results and to select the variants with support for a functional role based on the most informative annotations. We only considered cIMT for these analyses because of the small number of identified loci for carotid plaque. We first estimated the enrichment parameters for each annotation individually and identified the set of annotations with significant marginal associations. We then applied 10-fold cross-validation likelihood and forward selection to identify the set of annotations that significantly improve the model fit, and reverse selection of each annotation included in the model, as suggested in the fGWAS workflow. We reported the model with the highest cross-validation likelihood and SNPs that have regional posterior probability of association (PPA) >0.9 and directly overlap the genomic annotations considered.

### Overall genetic correlation analysis

Genetic correlation between cIMT/carotid plaque, CHD, and stroke traits were calculated using LD score regression approach LD-score, which uses GWAS summary statistics and is not affected by sample overlap. This method relies on the fact that the *χ*^2^ association statistic for a given SNP includes the effects of all SNPs that are in LD with it and it calculates genetic correlation by partitioning the SNP heritabilities^15^. Genetic correlations between stroke traits (IS, CE, large vessel disease, and SVD) and cIMT and carotid plaque were calculated using software available at http://github.com/bulik/ldsc with GWAS summary statistics for our cIMT/carotid plaque GWAS, CARDIOGRAMPlusC4D data, and stroke GWAS. We used the LD-scores^15^, which are based on the 1000 Genomes European population and estimated within 1-cM windows. Based on ten tests performed (two subclinical traits and five outcomes), we set the significance threshold to *p* = 0.005.

*PATHWAY ANALYSES*. *Methods for GO Slim*: The Ensembl identifiers of all protein-coding genes identified as in LD with the 12 variants for cIMT and 15 variants for carotid plaque (including variants from main and suggestive signals, Table 1 and Supplementary Table 5), and five genes for which there is strong evidence of colocalization (Table 3), were mapped to UniProt accession numbers, using the UniProt ID mapping service (http://www.uniprot.org/uploadlists/). A GO Slim analysis was performed on this list using QuickGO (www.ebi.ac.uk/QuickGO) and the Generic GO Slim. The GO terms used in the final slim analysis were further refined by adding/removing GO terms to provide more detailed information about the processes covered.

*Methods for GO term enrichment analysis*: The VLAD gene list analysis and visualization tool (http://proto.informatics.jax.org/prototypes/vlad/) was used to perform a GO term enrichment analysis on the same UniProt accessions as listed for the GO Slim. The background annotation set was obtained from the goa_human.gaf file (dated 21 November 2017, downloaded from ftp://ftp.ebi.ac.uk/pub/databases/GO/goa/HUMAN/) and the ontology data was obtained from the go-basic.obo file provided in the VLAD tool (analysis run 28 November 2017).

The LD block around top SNPs associated with cIMT and carotid plaque was constructed using LD information from the 1000 Genomes panel, as previously outlined in Finan et al.^16^. Briefly, the boundaries of the LD region were defined as the positions of the variants furthest upstream and downstream of a GWAS SNP with an *r*^2^ value of ≥0.5 and within a 1-Mbp flank on either side of the GWAS variant. Associated variants that were not present in the 1000 Genomes panel that were not in LD with any other variants were given a nominal flank of 2.5 kbp on either side of the association. Gene annotations using Ensembl version 79 were then overlapped to the LD region.

### Druggable genes

We examined the druggability status for the nearest coding genes identified in our GWAS analysis on cIMT and carotid plaque, including significant (novel and replicated) and suggestive ones, as well as genes identified through colocalization analysis. The druggable gene set was calculated using the previously described criteria: novel targets of first-in-class drugs licensed since 2005; the targets of drugs currently in late phase clinical development; pre-clinical phase small molecules with protein binding measurements reported in the ChEMBL database; and genes encoding secreted or plasma membrane proteins that are targets of monoclonal antibodies and other bio-therapeutics^16^. We defined three tiers of druggable gene sets based on their drug development. In Tier 1, 1427 genes were targets of approved small molecules and biotherapeutic drugs and clinical-phase drug candidates. Tier 2 comprised 682 genes encoding targets with known bioactive drug-like small molecule binding partners and those with significant sequence similarity to approved drug targets. Tier 3 contained 2370 genes encoding secreted or extracellular proteins, proteins with more distant similarity to approved drug targets, and druggable genes not included in Tier 1 or 2 such as GPCRs, nuclear hormone receptors, ion channels, kinases, and phosphodiesterases.

### URLs

For GTEx, see http://gtexportal.org/. For Coloc, see https://cran.r-project.org/web/packages/coloc/coloc.pdf. For, Moloc, see https://github.com/clagiamba/moloc/blob/master/man/moloc-package.Rd. For CARDIoGRAMPlusC4D, see www.cardiogramplusc4d.org/. For LD scores, www.broadinstitute.org/~bulik/eur_ldscores/. For UniProt ID, www.uniprot.org/uploadlists/. For QuickGO, www.ebi.ac.uk/QuickGO. For VLAD tool, see http://proto.informatics.jax.org/prototypes/vlad/.

## Electronic supplementary material

Supplementary Information

Supplementary Data 1

Peer Review File

Supplementary Data 2

Description of Additional Supplementary Files

## Data Availability

All relevant summary statistics data that support the findings of this study have been deposit in the database of Genotypes and Phenotypes (dbGaP) under the CHARGE acquisition number (https://www.ncbi.nlm.nih.gov/projects/gap/cgi-bin/study.cgi?study_id=phs000930.v6.p1; accession phs000930.v6.p1). GWAS data for most US studies are already available in dbGAP.
