# Supplementary material for: GWAS and colocalization analyses implicate carotid intima-media thickness and carotid plaque loci in cardiovascular outcomes
Source: Nat Commun. 2018 Dec 3;9:5141. doi: 10.1038/s41467-018-07340-5 (PMC6277418; doi:10.1038/s41467-018-07340-5)
Supplement: Supplementary file 1 — Supplementary Information [file 41467_2018_7340_MOESM1_ESM.pdf]

SUPPLEMENTARY INFORMATION

Genome-wide association study and colocalization analyses implicate carotid intima-media thickness and carotid plaque loci in cardiovascular outcomes

Franceschini, Giambartolomei et al.

Supplementary Note 1

Study Descriptions

This study includes data from the CHARGE and UCLEB Consortia. For all studies, each participant provided written informed consent. The Institutional Review Board at the parent institution for each respective study approved the study protocols.

CHARGE Consortium

The Aging Gene-Environment Susceptibility-Reykjavik Study (AGES) cohort originally comprised a random sample of 30,795 men and women born in 1907–1935 and living in Reykjavik in 1967.[1] A total of 19,381 individuals attended, resulting in 71% recruitment rate. The study sample was divided into six groups by birth year and birth date within month. One group was designated for longitudinal follow-up and was examined in all stages. One group was designated a control group and was not included in examinations until 1991. Other groups were invited to participate in specific stages of the study. Between 2002 and 2006, the AGES-Reykjavik study re-examined 5764 survivors of the original cohort who had participated before in the Reykjavik Study. The AGES Reykjavik Study GWAS was approved by the National Bioethics Committee (00-063-V8+1) and the Data Protection Authority.

The Atherosclerosis Risk in Communities Study (ARIC) is a multi-center prospective investigation of atherosclerotic disease in a predominantly bi-racial population. [2] Men and women aged 45-64 years at baseline were recruited from 4 communities: Forsyth County, North Carolina; Jackson, Mississippi; suburban areas of Minneapolis, Minnesota; and Washington County, Maryland. A total of 15,792 individuals participated in the baseline examination in 1987-1989, with follow-up examinations in approximate 3-year intervals, during 1990-1992, 1993-1995, and 1996-1998. ARIC Study samples were genotyped using the Affymetrix Genome-Wide Human SNP Array 6.0 (Santa Clara, California).

The Austrian Stroke Prevention Study (ASPS) study is a single center prospective follow-up study on the effects of vascular risk factors on brain structure and function in the normal elderly population of the city of Graz, Austria. The procedure of recruitment and diagnostic work-up of study participants has been described previously.[3,4] A total of 2007 participants were randomly selected from the official community register stratified by gender and 5 year age groups. Individuals were excluded from the study if they had a history of neuropsychiatric disease, including previous stroke, transient ischemic attacks, and dementia, or an abnormal neurologic examination determined on the basis of a structured clinical interview and a physical and neurologic examination. During 2 study periods between September 1991 and March 1994 and between January 1999 and December 2003 an extended diagnostic work-up including MRI and neuropsychological testing was done in 1076 individuals aged 45 to 85 years randomly selected from the entire cohort: 509 from the first period and 567 from the second. In 1992, blood was drawn from all study participants for DNA extraction. They were all European Caucasians.

The Austrian Stroke Prevention Family Study (ASPS-Fam) is a prospective single-center, community-based study on the cerebral effects of vascular risk factors in the normal elderly population of the city of Graz, Austria.[5,6] The ASPS-Fam represents an extension of ASPS, which was established in 1991.[3,4] Between 2006 and 2013, study participants of the ASPS and their first grade relatives were invited to enter ASPS-Fam. Inclusion criteria were no history of previous stroke or dementia and a normal neurologic examination. A total of 381 individuals from 169 families were included into the study. The number of members per family ranged from two to six. The entire cohort underwent an extended diagnostic work- up including clinical history, blood tests, cognitive testing, and a thorough vascular risk factor assessment. The study protocol was approved by the ethics committee of the Medical University of Graz, Austria, and written informed consent was obtained from all subjects.

Carotid Atherosclerosis Progression Study (CAPS) is a community-based study from Germany. Details of the study have been published before. [7] In brief, members of a German primary health care service population (n=32708) were invited to participate. Within a predefined time limit 6962 (21.3%) agreed to participate. Of these, 5,056 were invited to follow-up examination after three years and 3383 (67%) participated. 1,000 individuals in whom carotid IMT measurements were performed, and in whom there was sufficient DNA for investigation, were genotyped and data on these individuals contributed to this study. Informed written consent was obtained from all participants, and the study protocol was approved by the local ethical committee.

The Cardiovascular Health Study (CHS) is a population-based cohort study of risk factors for CHD and stroke in adults ≥65 years conducted across four field centers in the United States.[8] The original predominantly Caucasian cohort of 5201 persons was recruited in 1989-1990 from a random sample of people on Medicare eligibility lists and an additional 687 African-Americans were enrolled subsequently for a total sample of 5888. DNA was extracted from blood samples drawn on all participants who consented to genetic testing at their baseline examination in 1989-90. In 2007-2008, genotyping was performed at the General Clinical Research Center's Phenotyping/Genotyping Laboratory at Cedars-Sinai using the Illumina 370CNV Duo® BeadChip system on the 3980 CHS participants who were free of CVD at baseline.

Diabetes Heart Study (DHS) is a family-based observational cohort study of cardiovascular disease from a single research center in the United States. [9] The original predominantly (85%) European-ancestry cohort of 1443 persons was recruited in 1997-2005 from families with at least two type 2 diabetes affected siblings and, if possible, a non-diabetic sibling. Blood samples were drawn from all participants at their baseline examination and DNA was subsequently extracted from available samples.

The Erasmus Rucphen Family Study (ERF) is comprised of a family-based cohort embedded in the Genetic Research in Isolated Populations (GRIP) program in the southwest of the Netherlands. [10] The aim of this program is to identify genetic risk factors for the development of complex disorders. In ERF, twenty-two families that had a minimum of five children baptized in the community church between 1850 and 1900 were identified with the help of detailed genealogical records. All living descendants of these couples, and their spouses, were invited to take part in the study. Comprehensive interviews, questionnaires, and examinations were completed at a research center in the area; approximately 3,200 individuals participated. The examination included the determination of carotid intima media thickness and plaque scores via ultrasonography. Data collection started in June 2002 and was completed in February 2005.

The Framingham Heart Study (FHS). The methods of recruitment and data collection have been described previously for the original Framingham Heart Study cohort (5,209 participants ascertained systematically from two-thirds of the households in the town of Framingham, MA, beginning in 1948),[11] the Framingham Heart Study Offspring cohort (5,124 children of the original cohort, and spouses of those children, beginning in 1972[12] and the Third Generation cohort (4,095 children of the Offspring cohort, beginning in 2002).[13] The current study was conducted in 3,022 participants of the Offspring cohort participating in examination 6 from 1995 to 1998, who underwent contemporaneous carotid ultrasonography examination. Genotyping was conducted for the SNP Health Association Resource (SHARe) project (http://www.ncbi.nlm.nih.gov/projects/gap/cgi-bin/study.cgi?study_id=phs000007.v10.p5) using the Affymetrix 500K mapping array (250K Nsp and 250K Sty arrays) and the Affymetrix 50K supplemental gene focused array on a total of 9,274 individuals from all three cohorts. The Framingham Heart Study was approved by the institutional review boards of Boston University and the National Institutes of Health. All participants provided written informed consent.

The Three-City Study (3C) is a prospective population-based cohort study conducted in three French cities, Bordeaux, Dijon, Montpellier, comprising 9,294 participants in total. [14] To be eligible participants had to live in the city, be registered on the electoral rolls in 1999, 65 years or older, and not institutionalized. The study protocol was approved by the Ethical Committee of the University Hospital of Kremlin-Bicêtre and each participant signed an informed consent. In the 3C-Dijon study 4,931 participants were recruited between March 1999 and March 2001. A carotid ultrasound examination was proposed to participants under the age of 85 (n=4,580), and performed with a high resolution B-mode system (Ultramark 9 High Definition Imaging) and a 5- to 10-MHz sounding. Owing to financial and logistic reasons, ultrasound examinations were not performed during the last 6 months of the baseline phase. In total 3,323 participants with ultrasound measures are available in 3C-Dijon. Using a standardized protocol both the left and right common carotid arteries, bifurcations and the internal carotid arteries (first 2cm) were scanned. [15] DNA samples of 3C-Dijon participants were genotyped at the Centre National de Génotypage, Evry, France (www.cng.fr), using Illumina Human610 Quad BeadChip systems on 4077 individuals. [16] After exclusion of individuals > 80 years, with a history of surgical procedure on the carotid artery, and without genome-wide genotypes, 2,518 participants had measurements of common carotid artery intima-media thickness and 2,473 participants had measurement of carotid plaque.

The Lothian Birth Cohort 1936 (LBC1936) is a longitudinal study of ageing, derived from the Scottish Mental Survey of 1947, where nearly all 11 year-old children in Scotland were given a test of general cognitive ability [17-19]. Survivors living in the Lothian area of Scotland were recruited in late-life at mean age 70 (n=1,091). Follow-up has taken place at ages 70, 73, 76, and 79 years. Collected data include genetic information, longitudinal epigenetic information,

longitudinal brain imaging, and numerous blood biomarkers, anthropomorphic and lifestyle measures. CCA intima–media thickness (IMT) was measured manually with calipers [20]. This measures minimum, maximum and mean IMT over a 1 cm long segment of the common carotid artery and carotid bulb using the average of three measurements. The means of the maximum values were used with right and left measurements combined. carotid flow velocities, maximum stenosis affecting the internal carotid artery/bulb/CCA Plaques were defined by carotid stenosis of 25% or greater. Full measurement details are presented in Wardlaw et al. 2014[21]. Ethics permission for the study was obtained from the Multi-Centre Research Ethics Committee for Scotland (MREC/01/0/56) and from Lothian Research Ethics Committee (LBC1936: LREC/2003/2/29). The research was carried out in compliance with the Helsinki Declaration. All subjects gave written, informed consent.

MESA (Multi-Ethnic Study of Atherosclerosis) is a study of the characteristics of subclinical cardiovascular disease and the risk factors that predict progression to clinically overt cardiovascular disease or progression of the subclinical disease. [22] MESA consisted of a diverse, population-based sample of an initial 6,814 asymptomatic men and women aged 45-84. 38 percent of the recruited participants were white, 28 percent African American, 22 percent Hispanic, and 12 percent Asian, predominantly of Chinese descent. Participants were recruited from six field centers across the United States: Wake Forest University, Columbia University, Johns Hopkins University, University of Minnesota, Northwestern University and University of California - Los Angeles. Participants are being followed for identification and characterization of cardiovascular disease events, including acute myocardial infarction and other forms of coronary heart disease (CHD), stroke, and congestive heart failure; for cardiovascular disease interventions; and for mortality. The first examination took place over two years, from July 2000 - July 2002. It was followed by four examination periods that were 17-20 months in length. Participants have been contacted every 9 to 12 months throughout the study to assess clinical morbidity and mortality.

The Netherlands Epidemiology of Obesity (NEO) study was designed for extensive phenotyping to investigate pathways that lead to obesity-related diseases. [23] The NEO study is a population-based, prospective cohort study that includes 6,671 individuals aged 45–65 years, with an oversampling of individuals with overweight or obesity. At baseline, information on demography, lifestyle, and medical history have been collected by questionnaires. In addition, samples of 24-h urine, fasting and postprandial blood plasma and serum, and DNA were collected. Genotyping was performed using the Illumina HumanCoreExome chip, which was subsequently imputed to the 1000 genome reference panel. Participants underwent an extensive physical examination, including anthropometry, electrocardiography, spirometry, and measurement of the carotid artery intima-media thickness by ultrasonography. In random subsamples of participants, magnetic resonance imaging of abdominal fat, pulse wave velocity of the aorta, heart, and brain, magnetic resonance spectroscopy of the liver, indirect calorimetry, dual energy X-ray absorptiometry, or accelerometry measurements were performed. The collection of data started in September 2008 and completed at the end of September 2012. Participants are currently being followed for the incidence of obesity-related diseases and mortality.

The Netherlands Study of Depression and Anxiety (NESDA) is a multi-centre study designed to examine the long-term course and consequences of depressive and anxiety disorders (http://www.nesda.nl). [24] NESDA included both individuals with depressive and/or anxiety disorders and controls without psychiatric conditions. Inclusion criteria were age 18-65 years and self-reported western European ancestry, exclusion criteria were not being fluent in Dutch and having a primary diagnosis of another psychiatric condition (psychotic disorder, obsessive compulsive disorder, bipolar disorder, or severe substance use disorder). For all participants DNA was isolated from the baseline blood sample. Through funding from the NIH GAIN program (www.fnih.gov/gain), whole genome scan analysis was conducted for 1859 NESDA (1702 depressed cases and 157 controls) participants. A hundred subjects were excluded because of various quality control issues. [25]

The Orkney Complex Disease Study (ORCADES) is an ongoing family-based, cross-sectional study in the isolated Scottish archipelago of Orkney. [26] Genetic diversity in this population is decreased compared to Mainland Scotland, consistent with the high levels of endogamy historically. Fasting blood samples were collected and over 200 health-related phenotypes and environmental exposures were measured in each individual. All participants gave informed consent and the study was approved by Research Ethics Committees in Orkney and Aberdeen. Genotyping was performed with the Illumina HumanHap300 and Illumina Omni Express beadchips.

Rotterdam Study I and Rotterdam Study II (RS I and RS II). The Rotterdam Study is a prospective population-based cohort study to investigate the determinants of chronic diseases among participants aged 55 years and older.[27] Briefly, residents of Ommoord, a district of Rotterdam, in the Netherlands, 55 years of age or older, were asked to participate, of

whom 7,983 participated (RS I). The baseline examination was conducted in 1990 - 1993 and consisted of a home interview and research center visit for blood samples. In 1999, inhabitants who turned 55 years of age or moved into the study district since the start of the study were invited of whom 3011 participated (RS II). The Medical Ethics Committee of Erasmus MC approved the study, and all participants gave informed consent.

The Study of Health in Pomerania (SHIP) and SHIP-TREND. The Study of Health in Pomerania (SHIP) is a population-based study in the North-East of Germany, which consists of two independent prospectively collected cohorts (SHIP and SHIP-TREND)[28]. Their aim is assessing the prevalence and incidence of common population-based diseases and their risk factors. The detailed study design has been published previously. In brief, a sample from the population aged 20 to 79 years was drawn from population registries. First, the three cities of the region (with 17,076 to 65,977 inhabitants) and the 12 towns (with 1,516 to 3,044 inhabitants) were selected, and then 17 out of 97 smaller towns (with less than 1,500 inhabitants), were drawn at random. Second, from each of the selected communities, subjects were drawn at random, proportional to the population size of each community and stratified by age and gender. Only individuals with German citizenship and main residency in the study area were included. For SHIP, baseline examinations were performed between 1997 and 2001. The sample finally comprised 4,308 participants. SHIP-TREND finally comprised 4420 participants. Baseline examinations were conducted between 2008 and 2012. Individuals of both cohorts were analyzed separately. The carotid arteries were assessed with ultrasonography in participants at age 45 or older. Data on IMT and carotid plaques are available in 2,438 participants, of which 2,321 consented to take part in genome-wide association studies. The SHIP samples were genotyped using the Affymetrix Human SNP Array 6.0.

The Cardiovascular Risk In Young Finns (YFS) study. YFS is a Finnish multi-centre study that was initiated in 1980. [29] A total of 3596 children and adolescents aged 3-18 years participated in the first cross-sectional study. Study variables since childhood include serum lipids, blood pressure, obesity indices, insulin, glucose, life-style (diet, smoking, physical activity, alcohol), family risk and socioeconomic status. In addition, national register data on all hospitalizations with specific diagnoses is available from 1969 onwards. In adulthood, follow-up visits have been performed in 2001, 2007, and 2011, with a total of 2,800 individuals from childhood having at least one follow-up in adulthood. The follow-up studies in 2001 and 2007 have included non-invasive ultrasound measurements of arterial function and structure, which are indicative of subclinical atherosclerosis. [29] DNA was extracted from blood samples drawn on all participants in 2001 and 2007. In 2009 genotyping was performed at the Sanger institute (UK) using the custom-built Illumina BeadChip Human670K from 2442 YFS participants (1123 males, 1319 females) including 546677 SNPs.

UCLEB (UNIVERSITY COLLEGE-LONDON SCHOOL-EDINBURGH-BRISTOL) CONSORTIUM

BRHS. From 1978 to 1980, 7735 men aged 40-59 were recruited from general practices across the UK.[30] A wide range of phenotypic measures is available for established risk markers such as lipids, blood pressure and inflammatory and hemostatic markers. Most of these measures were taken both at recruitment and re-examination, which occurred in 1998-2000 when men were aged 60-79. At this re-examination 4,252 participants attended and DNA was extracted for 3945. Data on important behavioral variables such as cigarette and alcohol consumption, as well as physical activity, have been regularly collected through follow up. Well validated outcome variables including major coronary heart disease and stroke, as well as cause-specific mortality, continue to be collected from medical records 30 years after recruitment.

The Edinburgh Artery Study (EAS) is an age-stratified random sample of men and women, aged 55-74 years, which was selected between August 1987 and September 1988 from the age-sex registers of ten general practices with a geographical and socio-economical catchment population spread throughout the city of Edinburgh, UK.[30] Subjects were excluded if they were unfit to participate (e.g. due to severe mental illness or terminal disease); excluded individuals were replaced by other randomly sampled subjects.

The Edinburgh Type 2 Diabetes Study (ET2DS) is based on an age-stratified random sample of men and women with type 2 diabetes, aged 60–74 years, which was selected between August 2006 and August 2007 from the Lothian Diabetes Register (LDR), a comprehensive database of subjects with known type 2 diabetes living in Lothian.[31] Subjects were excluded if they did not meet WHO criteria for type 2 diabetes, or if they were physically unable to complete the clinical and cognitive examination. The study population is almost exclusively European. DNA was extracted at baseline. Physical examinations were performed by specially trained research nurses using standardised operating procedures. The quality of measurements was checked using observation of research staff by study investigators and inter-observer variability assessments were made for key variables. Blood assays were performed in accredited laboratories using

international standards. Retrospective data on cardiovascular disease and selected physical and biochemical variables were retrieved using record linkage for hospitalisations and deaths since 1985 and using data from the LDR. Subjects returned for further clinical examination after one year and were examined again after they had participated for 4 years.

MRC1946. The Medical Research Council (MRC) National Survey of Health and Development (NSHD; also known as MRC 1946 birth cohort) is an on-going prospective birth cohort study consisting of a sample of all singleton births, born to married mothers, in England, Scotland and Wales in one week in March 1946. [32] The sample includes all births whose fathers were in non-manual or agricultural occupations and a randomly selected one in four of all others, whose fathers were in manual occupations. The original cohort comprised 2,547 women and 2,815 men who have been followed up over 20 times since their birth. The data collected to date include cognitive function, physical, lifestyle and anthropomorphic measures as well as blood analytes and other measures. Through MRC Unit funding, a particularly intensive clinical assessment, with biological sampling, blood and urine sampling and analysis, and cardiac and vascular imaging has recently been completed when the cohort were aged 60-64 years.

The Whitehall II (WHII) Study recruited 10,308 participants (70% men) between 1985 and 1989 from 20 London based civil service departments.[33] In this longitudinal study blood pressure was recorded at phase 1 (1985-1988), phase 3 (1991-1993), phase 5 (1997-1999) and phase 7 (2003-2004). DNA was stored from phase 7 from over 6,000 participants. The study participants are all highly phenotyped for cardiovascular and other ageing related health outcomes.

IMPROVE is a multicentre, longitudinal, observational study, which involves seven recruiting centres in five European countries: Finland, France, Italy, the Netherlands, and Sweden.[34] Each recruiting centre was incorporated separately into the analysis. Recruitment of a total of 3598 patients (514 per centre) was targeted. Men and women, aged from 55 to 79 years, with at least three vascular risk factors, asymptomatic for cardiovascular diseases and free of any conditions that might limit longevity or IMT visualization were considered as eligible for the study. The primary objective of the IMPROVE study was to evaluate the association between C-IMT progression at 15 months and future vascular events (myocardial infarction, cardiovascular death, stroke, or any intervention in the carotid, coronary, or peripheral arterial districts occurring from the 15th to the 36th month of follow-up).

LIFE-Adult is a population-based cohort of 10,000 adult inhabitants of the city of Leipzig, Germany. [35] Participants were characterized regarding life-style and environmental risk factors and clinical and subclinical signs of diseases such as cardiovascular diseases, type 2 diabetes or cognition. Detailed description of the cohort can be found elsewhere. [35]LIFE-Adult meets the ethical standards of the Declaration of Helsinki. The study is approved by the Ethics Committee of the Medical Faculty of the University Leipzig, Germany (Reg. No 263-2009-14122009). Written informed consent including agreement with genetic analyses was obtained from all participants. High-resolution B-mode ultrasound images of carotid vessels were acquired using the GE Vivid ultrasound platform with a 12.0-MHz linear-array transducer (GE-Healthcare). For the assessments, subjects were in supine position. Genotyping was performed using the Affymetrix Axiom CEU1 SNP-array technology.

LIFE-Heart is a cohort of patients with suspected or confirmed stable coronary artery disease or myocardial infarction collected at the Heart Center of the University of Leipzig, Germany. Study details can be found elsewhere. [36] A total of about 7,000 patients were recruited. LIFE-Heart meets the ethical standards of the Declaration of Helsinki. The study is approved by the Ethics Committee of the Medical Faculty of the University Leipzig, Germany (Reg. No 276-2005) and is registered at ClinicalTrials.gov (NCT00497887). Written informed consent including agreement with genetic analyses was obtained from all participants. Patients with myocardial infarction were excluded from the present analysis. High-resolution B-mode ultrasound images of carotid vessels were acquired using the GE Vivid ultrasound platform with a 12.0-MHz linear-array transducer (GE-Healthcare). For the assessments, subjects were in supine position. Genotyping was performed with either Affymetrix Axiom CEU1 or Affymetrix Axiom CADLIFE. The latter is an array containing Axiom CEU as genome-wide backbone and an additional custom content of about 62,500 SNPs from CAD loci.

The Prospective Investigation of the Uppsala Seniors (PIVUS) cohort was randomly sampled from all men and women at age 70 living in Uppsala County in 2001 (n=1016; www.medsci.uu.se/PIVUS). Follow-ups were made at years 75 (n=827) and 80 (n=606). The participants underwent a medical examination with cognitive testing (MMSE), vascular status assessments (endothelium-dependent vasodilation, flow-mediated dilation and pulse-wave velocity), subclinical atherosclerosis measurements (intima-media thickness, grey-scale median and plaque occurrence) and blood sampling

(low-density lipoprotein, high-density lipoprotein, triglycerides and total cholesterol) including a detailed questionnaire (medical history, exercise, smoking, alcohol, dietary habits and educational level). All participants were genotyped using the Ilumina MetaboChip genotyping array.

ALSPAC The Avon Longitudinal Study of Parents and Children (ALSPAC) (http://www.alspac.bristol.ac.uk/) recruited 14,541 pregnant women resident in Avon, UK with expected dates of delivery 1st April 1991 to 31st December 1992. Further details of the cohort and data collection are available in previous publications. [37,38] The study website (http://www.bristol.ac.uk/alspac/researchers/) contains details of all the data that is available through a fully searchable data dictionary. For this study data from a sub-sample of the women who were originally recruited when pregnant and who attended a follow-up clinic approximately 18-years after the birth of the study index child were included. All data collection and its use for research has been approved by the ALSPAC Ethics and Law Committee and/or UK National Health Service Research Ethics Committees. Participants provided informed written consent. cIMT measurements on these women were collected from both the left and right common carotid artery arteries, using high-resolution B ultrasound and scanning longitudinally 1 cm proximal to the carotid bifurcation following a standardized protocol. A ZONARE z.one Ultra convertible ultrasound system with L10-5 linear transducer was used. Images were focused on the posterior (far) wall of the artery and the zoom function was used to magnify the area. Ten-second cine loops were recorded in DICOM format and analyzed offline using Carotid Analyzer for Research (Vascular Research Tools 5, Medical Imaging Applications, LLC 2008). Three consecutive cardiac cycles were identified and three measures of cIMT were taken from end-diastolic frames and averaged. This was done for both right and left carotid arteries. Arterial distensibility was calculated as the difference between systolic and diastolic arterial diameter. The mean of the left- and right-sided readings was used in all analyses. The images were analyzed by a single trained reader.

The Nijmegen Biomedical Study (NBS) (http://www.nijmegenbiomedischestudie.nl) is a population-based survey conducted by the Department for Health Evidence and the Department of Laboratory Medicine of the Radboud University Medical Centre, Nijmegen, The Netherlands. A cohort profile description of the NBS is available. [39] Briefly, in 2002, 22,451 age and sex-stratified randomly selected adult inhabitants of Nijmegen, a city located in the eastern part of the Netherlands, received an invitation to fill out a postal questionnaire (QN) including questions about lifestyle, health status, and medical history, and to donate a blood sample for DNA isolation and biochemical studies. A total of 9350 (43%) persons filled out the QN, of which 6468 (69%) donated blood samples. A second, third and fourth questionnaire were sent out in 2005, 2008 and 2012, respectively. Approval to conduct the NBS was obtained from the Radboud university medical center Institutional Review Board. All participants gave written informed consent for participation in the NBS.

The Malmo Diet and Cancer (MDC) study is set in Malmö, Sweden's third largest city.[40] The background population consisted of all men born between 1923 and 1945 and all women born between 1923 and 1950 who were living in Malmö during the screening period 1991 to 1996 (n = 74,138). This population was identified through the Swedish national population registries. The final cohort consisted of 28,098 individuals (participation rate 40.8%). The subjects were recruited through advertisements in local media and through invitation by mail. The only exclusion criteria were inadequate Swedish language skills and mental incapacity. The Ethics Committee at Lund University approved the design of the MDC study (LU 51–90). Written informed consent was obtained from the participants.

Supplementary Note 2

Acknowledgements

AGES. This study has been funded by NIH contract N01-AG012100, the NIA Intramural Research Program, an Intramural Research Program Award (ZIAEY000401) from the National Eye Institute, an award from the National Institute on Deafness and Other Communication Disorders (NIDCD) Division of Scientific Programs (IAA Y2-DC_1004-02), Hjartavernd (the Icelandic Heart Association), and the Althingi (the Icelandic Parliament). The study is approved by the Icelandic National Bioethics Committee, VSN: 00-063. The researchers are indebted to the participants for their willingness to participate in the study.

ARIC. The ARIC study is carried out as a collaborative study supported by National Heart, Lung, and Blood Institute contracts (HHSN268201100005C, HHSN268201100006C, HHSN268201100007C, HHSN268201100008C, HHSN268201100009C, HHSN268201100010C, HHSN268201100011C, and HHSN268201100012C), R01HL087641, R01HL59367 and R01HL086694; National Human Genome Research Institute contract U01HG004402; and National Institutes of Health contract HHSN268200625226C. The authors thank the staff and participants of the ARIC study for their important contributions. Infrastructure was partly supported by Grant Number UL1RR025005, a component of the National Institutes of Health and NIH Roadmap for Medical Research.

ASPS/ASPS-Fam. The authors thank the staff and the participants for their valuable contributions. We thank Birgit Reinhart for her long-term administrative commitment, Elfi Hofer for the technical assistance at creating the DNA bank, Ing. Johann Semmler and Anita Harb for DNA sequencing and DNA analyses by TaqMan assays and Irmgard Poelzl for supervising the quality management processes after ISO9001 at the biobankingand DNA analyses. The research reported in this article was funded by the Austrian Science Fund (FWF) grant number P20545-P05, P13180, PI904 the Austrian National Bank Anniversary Fund, P15435, the Austrain Federal Ministry of Science, Research and Economy under the aegis of the EU Joint Programme-Neurodegenerative Disease Research (JPND)-www.jpnd.eu and by the Austrian Science Fund P20545-B05. The Medical University of Graz supports the databank of the ASPS.

Cardiovascular Health Study (CHS). Cardiovascular Health Study: This CHS research was supported by NHLBI contracts HHSN268201200036C, HHSN268200800007C, N01HC55222, N01HC85079, N01HC85080, N01HC85081, N01HC85082, N01HC85083, N01HC85086, N01HC85085, N01HC45133, HHSN268200960009C, N01HC85085, and N01HC45133; and NHLBI grants U01HL080295, R01HL087652, R01HL105756, R01HL103612, R01HL120393, and R01HL130114 with additional contribution from the National Institute of Neurological Disorders and Stroke (NINDS). Additional support was provided through R01AG023629 from the National Institute on Aging (NIA). A full list of principal CHS investigators and institutions can be found at CHS-NHLBI.org. The provision of genotyping data was supported in part by the National Center for Advancing Translational Sciences, CTSI grant UL1TR000124, and the National Institute of Diabetes and Digestive and Kidney Disease Diabetes Research Center (DRC) grant DK063491 to the Southern California Diabetes Endocrinology Research Center. The content is solely the responsibility of the authors and does not necessarily represent the official views of the National Institutes of Health.Diabetes Heart Study (DHS). This study was supported by the National Institutes of Health through R01 HL6734, R01 HL092301, R01 AR48797, and F31 AG044879. DHS. The authors thank the Wake Forest School of Medicine investigators and staff and the participants of the Diabetes Heart Study for their valuable contributions.

Three City Study (3C) Dijon. The 3-City Study is conducted under a partnership agreement among the Institut National de la Santé et de la Recherche Médicale (INSERM), the University of Bordeaux, and Sanofi-Aventis. The Fondation pour la Recherche Médicale funded the preparation and initiation of the study. The 3C Study is also supported by the Caisse Nationale Maladie des Travailleurs Salariés, Direction Générale de la Santé, Mutuelle Générale de l'Education Nationale (MGEN), Institut de la Longévité, Conseils Régionaux of Aquitaine and Bourgogne, Fondation de France, and Ministry of Research–INSERM Programme "Cohortes et collections de données biologiques." This work was supported by the National Foundation for Alzheimer's Disease and Related Disorders, the Institut Pasteur de Lille, the Centre National de Génotypage and the LABEX (Laboratory of Excellence program investment for the future) DISTALZ - Development of Innovative Strategies for a Transdisciplinary approach to ALZheimer's disease. Ganesh Chauhan, Christophe Tzourio and

Stéphanie Debette are supported by a grant from the Fondation Leducq and grants from the Agence Nationale de la Recherche (ANR).

ERF (Erasmus Rucphen Family study). The ERF study was supported by CardioVasculair Onderzoek Nederland (CVON2012-03) of the Netherlands Heart Foundation and the Rotterdam Study by the European Union's Horizon 2020 research and innovation programme as part of the Common mechanisms and pathways in Stroke and Alzheimer's disease (CoSTREAM) project (www.costream.eu, grant agreement No 667375); European Union’s Horizon 2020 research.

FHS (Framingham Heart Study). This research was conducted in part using data and resources from the Framingham Heart Study of the National Heart Lung and Blood Institute of the National Institutes of Health and Boston University School of Medicine. The analyses reflect intellectual input and resource development from the Framingham Heart Study investigators participating in the SNP Health Association Resource (SHARe) project. This work was partially supported by the National Heart, Lung and Blood Institute's Framingham Heart Study (Contract Nos. N01-HC-25195 and HHSN268201500001I) and its contract with Affymetrix, Inc for genotyping services (Contract No. N02-HL-6-4278). A portion of this research utilized the Linux Cluster for Genetic Analysis (LinGA-II) funded by the Robert Dawson Evans Endowment of the Department of Medicine at Boston University School of Medicine and Boston Medical Center.

GeneSTAR was supported by grants from the National Institutes of Health/National Heart, Lung, and Blood Institute (U01 HL72518, HL097698, HL49762, HL58625, HL071025, and HL092165), by a grant from the National Institutes of Health/National Institute of Nursing Research (NR0224103), and by a grant from the National Center for Research Resources and the National Center for Advancing Translational Sciences, National Institutes of Health to the Johns Hopkins Institute for Clinical & Translational Research (UL1 RR 025005).

JHS (Jackson Heart Study). We thank the Jackson Heart Study participants and staff for their contributions to this work. The JHS is supported by contracts HHSN268201300046C, HHSN268201300047C, HHSN268201300048C, HHSN268201300049C, HHSN268201300050C from the National Heart, Lung, and Blood Institute and the National Institute on Minority Health and Health Disparities. Dr. Wilson is supported by U54GM115428 from the National Institute of General Medical Sciences.

LBC1936. REM, IJD, and JMW are members of The University of Edinburgh Centre for Cognitive Ageing and Cognitive Epidemiology, part of the cross-council Lifelong Health and Wellbeing Initiative (MR/K026992/1). Funding from the Biotechnology and Biological Sciences Research Council (BBSRC) and Medical Research Council (MRC) is gratefully acknowledged.

MESA (Multi-Ethnic Study of Atherosclerosis). MESA and the MESA SHARe project are conducted and supported by the National Heart, Lung, and Blood Institute (NHLBI) in collaboration with MESA investigators. Support for MESA is provided by contracts HHSN268201500003I, N01-HC-95159, N01-HC-95160, N01-HC-95161, N01-HC-95162, N01-HC-95163, N01-HC-95164, N01-HC-95165, N01-HC-95166, N01-HC-95167, N01-HC-95168, N01-HC-95169, UL1-TR-000040, UL1-TR-001079, and UL1-TR-001420. This publication was developed under a STAR research assistance agreement, No. RD831697 (MESA Air), awarded by the U.S Environmental protection Agency. It has not been formally reviewed by the EPA. The views expressed in this document are solely those of the authors and the EPA does not endorse any products or commercial services mentioned in this publication. The provision of genotyping data was supported in part by the National Center for Advancing Translational Sciences, CTSI grant UL1TR001881, and the National Institute of Diabetes and Digestive and Kidney Disease Diabetes Research Center (DRC) grant DK063491 to the Southern California Diabetes Endocrinology Research Center. The authors thank the participants of the MESA study, the Coordinating Center, MESA investigators, and study staff for their valuable contributions. A full list of participating MESA investigators and institutions can be found at http://www.mesa-nhlbi.org.

NEO. The authors of the NEO study thank all individuals who participated in the Netherlands Epidemiology in Obesity study, all participating general practitioners for inviting eligible participants and all research nurses for collection of the data. We thank the NEO study group, Pat van Beelen, Petra Noordijk and Ingeborg de Jonge for the coordination, lab and data management of the NEO study. The genotyping in the NEO study was supported by the Centre National de

Génotypage (Paris, France), headed by Jean-Francois Deleuze. The NEO study is supported by the participating Departments, the Division and the Board of Directors of the Leiden University Medical Center, and by the Leiden University, Research Profile Area Vascular and Regenerative Medicine. Dennis Mook-Kanamori is supported by Dutch Science Organization (ZonMW-VENI Grant 916.14.023).

ORCADES was supported by the Chief Scientist Office of the Scottish Government (CZB/4/276, CZB/4/710), the Royal Society, the MRC Human Genetics Unit, Arthritis Research UK and the European Union framework program 6 EUROSPAN project (contract no. LSHG-CT-2006-018947). DNA extractions were performed at the Wellcome Trust Clinical Research Facility in Edinburgh. We would like to acknowledge the invaluable contributions of the research nurses in Orkney, the administrative team in Edinburgh and the people of Orkney.

YFS. Young Finns Study was financially supported by the Academy of Finland (134309 (Eye), 126925, 121584, 124282, 129378 (Salve), 117787 (Gendi), and 41071 (Skidi)); the Social Insurance Institution of Finland, Kuopio, Tampere; Turku University Hospital Medical Funds (grant 9M048 and 9N035 to T.L.); Juho Vainio Foundation; Paavo Nurmi Foundation; Finnish Foundation of Cardiovascular Research and Finnish Cultural Foundation; Tampere Tuberculosis Foundation; and Emil Aaltonen Foundation (to T.L.).

NESDA. Funding was obtained from the Netherlands Organization for Scientific Research (Geestkracht program grant 10-000-1002); the Center for Medical Systems Biology (CSMB, NWO Genomics), Biobanking and Biomolecular Resources Research Infrastructure (BBMRI-NL), University's Institutes for Health and Care Research (EMGO+) and Neuroscience Campus Amsterdam, University Medical Center Groningen, Leiden University Medical Center, National Institutes of Health (NIH, R01D0042157-01A, MH081802, Grand Opportunity grants 1RC2 MH089951 and 1RC2 MH089995). Part of the genotyping and analyses were funded by the Genetic Association Information Network (GAIN) of the Foundation for the National Institutes of Health. Computing was supported by BiG Grid, the Dutch e-Science Grid, which is financially supported by NWO. Statistical analyses were carried out on the Genetic Cluster Computer (http://www.geneticcluster.org) hosted by SURFsara and financially supported by the Netherlands Scientific Organization (NWO 480-05-003 PI: Posthuma) along with a supplement from the Dutch Brain Foundation and the VU University Amsterdam.

EAS. The Edinburgh Artery Study is funded by the British Heart Foundation (Programme Grant RG/98002), with Metabochip genotyping funded by a project grant from the Chief Scientist Office of Scotland (Project Grant CZB/4/672). ET2DS: The Edinburgh Type 2 Diabetes Study is funded by the Medical Research Council (Project Grant G0500877); the Chief Scientist Office of Scotland (Programme Support Grant CZQ/1/38); Pfizer plc (Unrestricted Investigator Led Grant); and Diabetes UK (Clinical Research Fellowship 10/0003985). Research clinics were held at the Welcome Trust Clinical Research Facility and Princess Alexandra Eye Pavilion in Edinburgh

Pivus. Swedish Research Council (grant no. 2015-02907), Göran Gustafsson Foundation, Swedish Heart-Lung Foundation (grant no. 20140422), Knut och Alice Wallenberg Foundation (grant no. 2013.0126)

ALSPAC. The UK Medical Research Council and Wellcome Trust (102215/2/13/2) and the University of Bristol provide core support for ALSPAC. A Wellcome Trust (WT088806) grant provided funds for completion of genome wide on the ALSPAC mothers. Phenotypic (cIMT) data collection was funded by the British Heart Foundation (SP/07 1008/24066), UK Medical Research Council (G1001357) and WellcomeTrust (WT092830M). DAL and SR work in a Unit that receives funds from the UK Medical Research Council (MC_UU_12013/5) and DAL is a National Institute of Health Research Senior Investigator (NF-SI-0611-10196). We are extremely grateful to all of the families who took part in this study, the midwives for their help in recruiting them, and the whole ALSPAC team, which includes interviewers, computer and laboratory technicians, clerical workers, research scientists, volunteers, receptionists, managers and nurses.

NBS. The Nijmegen Biomedical Study is a population-based survey conducted at the Department for Health Evidence, and the Department of Laboratory Medicine of the Radboud university medical center. Principal investigators of the Nijmegen Biomedical Study are L.A.L.M. Kiemeney, A.L.M. Verbeek, D.W. Swinkels and B. Franke

LIFE-Adult is funded by the Leipzig Research Center for Civilization Diseases (LIFE). LIFE is an organizational unit affiliated to the Medical Faculty of the University of Leipzig. LIFE is funded by means of the European Union, by the

European Regional Development Fund (ERDF) and by funds of the Free State of Saxony within the framework of the excellence initiative (project numbers 713-241202, 14505/2470, 14575/2470).

MALMO. This study was supported by grants from the European Research Council (StG-282255) Swedish Medical Research Council, the Swedish Heart and Lung Foundation, the Medical Faculty of Lund University, Malmö. University Hospital, the Albert Påhlsson Research Foundation, the Crafoord Foundation, the Ernhold Lundström Research Foundation, the Region Skane, Hulda and Conrad Mossfelt Foundation, King Gustaf V and Queen Victoria Foundation and the Lennart Hansson Memorial Fund. The authors acknowledge the Knut and Alice Wallenberg Foundation for its economic support of the SWEGENE DNA extraction facility.

BRHS. The British Regional Heart Study has been supported by Programme Grants from the British Heart Foundation (RG/08/013/25942 and RG/13/16/30528).

IMROVE. The authors wish to thank all the members of the IMPROVE group for their time and extraordinary commitment. The IMPROVE study was supported by the European Commission [Contract number: QLG1- CT- 2002-00896 to E.T., D.B., A.H., S.E.H., R.R., U.dF., A.J.S., P.G., S.K., E.M.], Ministero della Salute Ricerca Corrente, Italy [to E.T., D.B.], the Swedish Heart-Lung Foundation, the Swedish Research Council [projects 8691 to A.H. and 0593 to U.dF.], the Foundation for Strategic Research, the Stockholm County Council [project 562183 to A.H.], the Foundation for Strategic Research, the Academy of Finland [Grant #110413 to S.K.] and the British Heart Foundation [RG2008/008 to S.E.H.]. None of the aforementioned funding organizations or sponsors has had a specific role in design or conduct of the study, collection, management, analysis, or interpretation of the data, or preparation, review, or approval of the manuscript.

ORCADES was supported by the Chief Scientist Office of the Scottish Government (CZB/4/276, CZB/4/710), the Royal Society, the MRC Human Genetics Unit, Arthritis Research UK and the European Union framework program 6 EUROSPAN project (contract no. LSHG-CT-2006-018947). DNA extractions were performed at the Wellcome Trust Clinical Research Facility in Edinburgh. We would like to acknowledge the invaluable contributions of the research nurses in Orkney, the administrative team in Edinburgh and the people of Orkney.

The Rotterdam study is supported by the Erasmus MC and Erasmus University Rotterdam; the Netherlands Organisation for Scientific Research; the Netherlands Organisation for Health Research and Development (ZonMw: Zorg onderzoek Nederland Medische Wetenschappen); the Research Institute for Diseases in the Elderly; the Netherlands Genomics Initiative; the Ministry of Education, Culture and Science; the Ministry of Health, Welfare and Sports; the European Commission (Directorate-General XII); and the Municipality of Rotterdam. Maryam Kavousi is supported by the ZonMw Veni grant (Veni, 91616079). O.H. Franco works in ErasmusAGE, a center for aging research across the life course funded by Nestlé Nutrition (Nestec Ltd.); Metagenics Inc.; and AXA. None of the funders had any role in design and conduct of the study; collection, management, analysis, and interpretation of the data; and preparation, review, or approval of this article. The generation and management of GWAS genotype data for the Rotterdam study was executed by the Human Genotyping Facility of the Genetic Laboratory of the Department of Internal Medicine, Erasmus MC, Rotterdam, the Netherlands. The GWAS datasets are supported by the Netherlands Organisation of Scientific Research Investments (number: 175.010.2005.011, 911-03-012), the Genetic Laboratory of the Department of Internal Medicine, Erasmus MC, the Research Institute for Diseases in the Elderly (014-93-015), the Netherlands Genomics Initiative/Netherlands Organisation for Scientific Research Netherlands Consortium for Healthy Aging, project number: 050-060-810. We thank the Genetic Laboratory of the Department of Internal Medicine of the Erasmus MC and specifically Pascal Arp, Mila Jhamai, Marijn Verkerk, and Carolina Medina-Gomez for their help in creating the GWAS database and the creation and analysis of imputed data. The dedication, commitment, and contribution of inhabitants, general practitioners, and pharmacists of the Ommoord district to the Rotterdam Study are gratefully acknowledged

SHIP is part of the Community Medicine Research net of the University of Greifswald, Germany, which is funded by the Federal Ministry of Education and Research (grants no. 01ZZ9603, 01ZZ0103, and 01ZZ0403), the Ministry of Cultural Affairs as well as the Social Ministry of the Federal State of Mecklenburg-West Pomerania, and the network 'Greifswald Approach to Individualized Medicine (GANI_MED)' funded by the Federal Ministry of Education and Research (grant 03IS2061A). Genome-wide data have been supported by the Federal Ministry of Education and Research (grant no.

03ZIK012) and a joint grant from Siemens Healthcare, Erlangen, Germany and the Federal State of Mecklenburg- West Pomerania. The University of Greifswald is a member of the Caché Campus program of the InterSystems GmbH.

Supplementary Figures

Supplementary Figure 1. QQ plots for meta-analyses of cIMT (A) and plaque (B) and Manhattan plots for cIMT (C) and plaque (D). Novel loci highlighted in red.

A

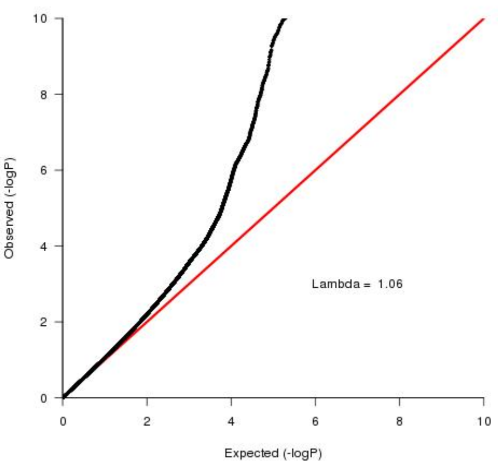

B

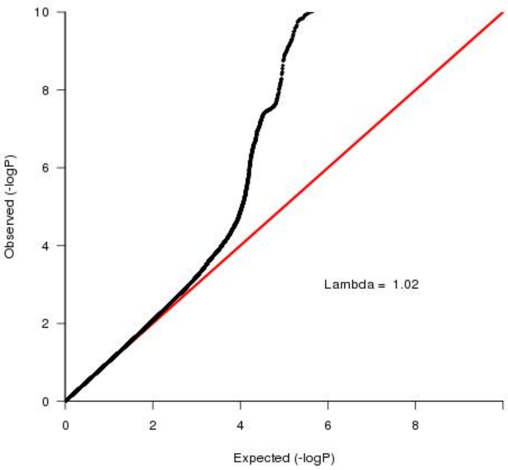

C

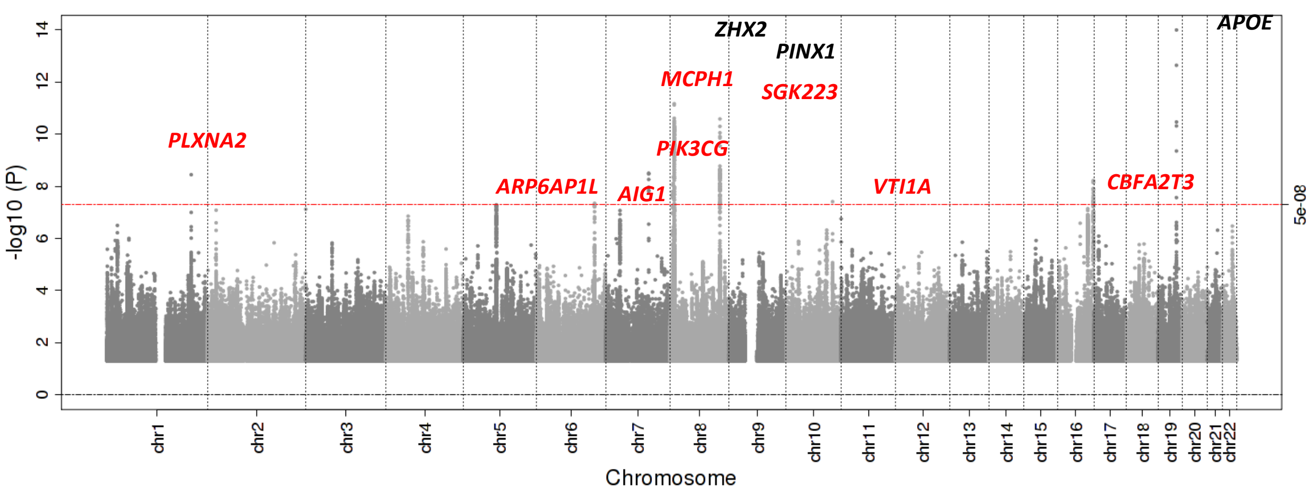

D

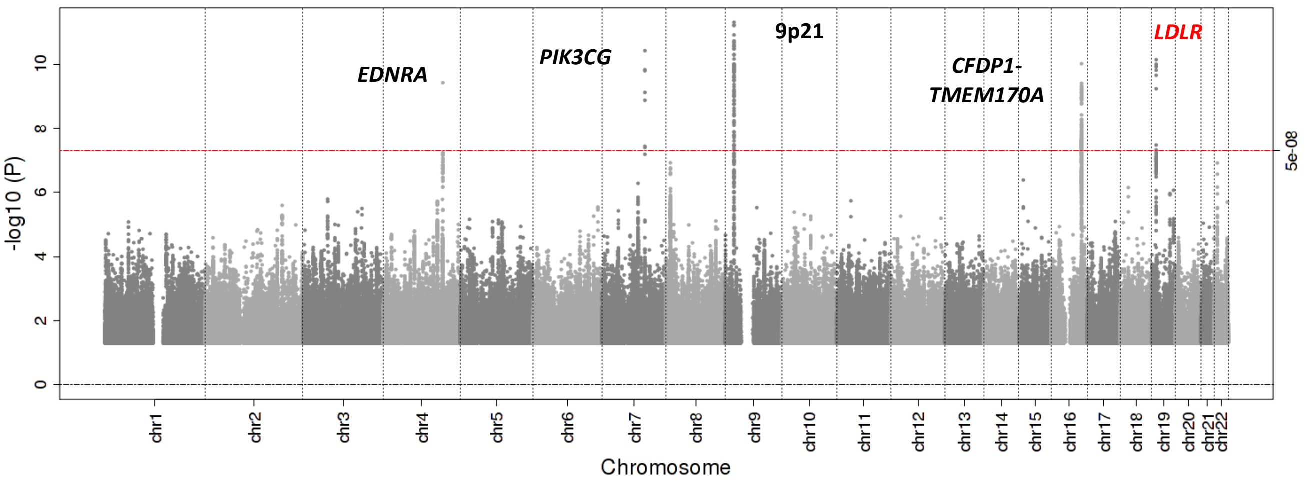

Supplementary Figure 2. Regional plots for significant loci for cIMT (A-K) and carotid plaque (L-P). Note the most significant SNP may not have LD with other SNPs.

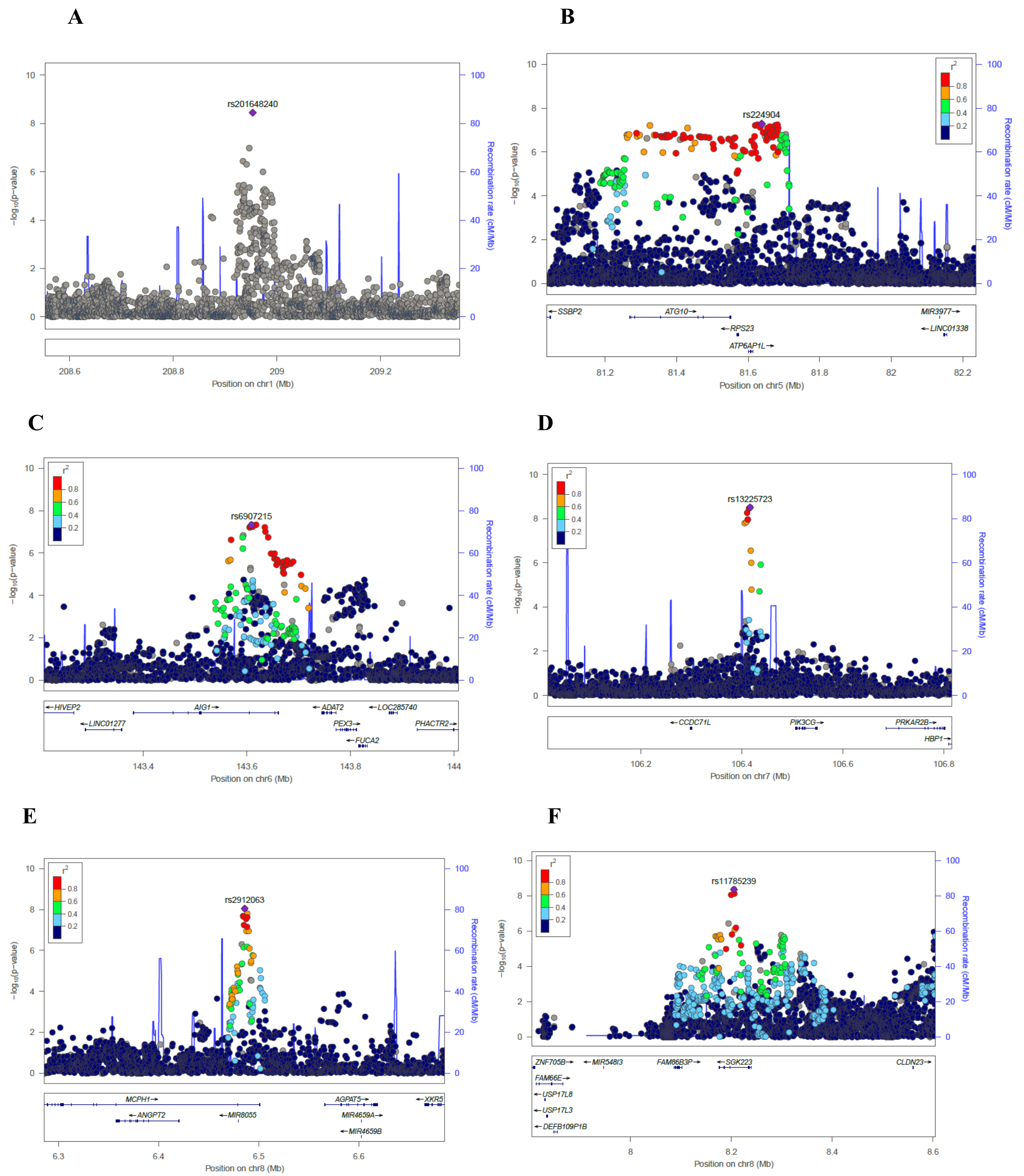

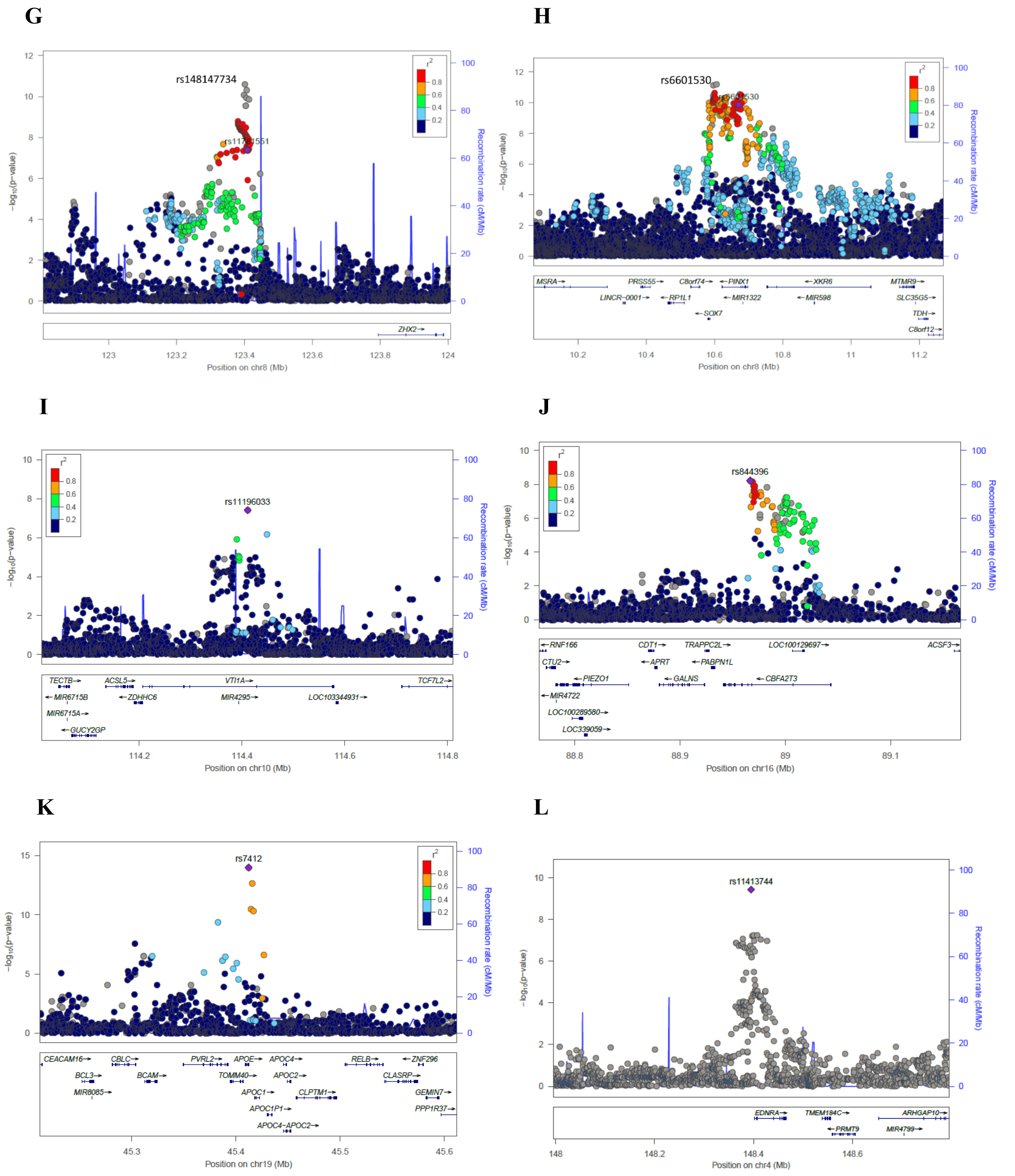
G
rs148147734
rs11781551
ZHX2→
Position on chr8 (Mb)
−log10(p−value)
Recombination rate (cM/Mb)
H
rs6601530
MSRA→
PRSS55→
C8orf74→
←PINX1
←XKR6
MTMR9→
LINCR−0001→
←RP1L1
←MIR1322
←MIR598
SLC35G5→
←SOX7
TDH→
C8orf12→
Position on chr8 (Mb)
I
rs11196033
TECTB→
ACSL5→
VTI1A→
TCF7L2→
←MIR6715B
←ZDHHC6
MIR4295→
LOC103344931→
MIR6715A→
←GUCY2GP
Position on chr10 (Mb)
J
rs844396
←RNF166
CDT1→
TRAPPC2L→
LOC100129697→
ACSF3→
CTU2→
←APRT
←PABPN1L
←PIEZO1
←GALNS
←CBFA2T3
←MIR4722
LOC100289580→
LOC339059→
Position on chr16 (Mb)
K
rs7412
CEACAM16→
CBLC→
PVRL2→
APOE→
APOC4→
RELB→
←ZNF296
BCL3→
BCAM→
TOMM40→
APOC2→
CLASRP→
MIR8085→
APOC1→
CLPTM1→
GEMIN7→
APOC1P1→
PPP1R37→
APOC4−APOC2→
Position on chr19 (Mb)
L
rs11413744
EDNRA→
TMEM184C→
ARHGAP10→
←PRMT9
MIR4799→
Position on chr4 (Mb)

M

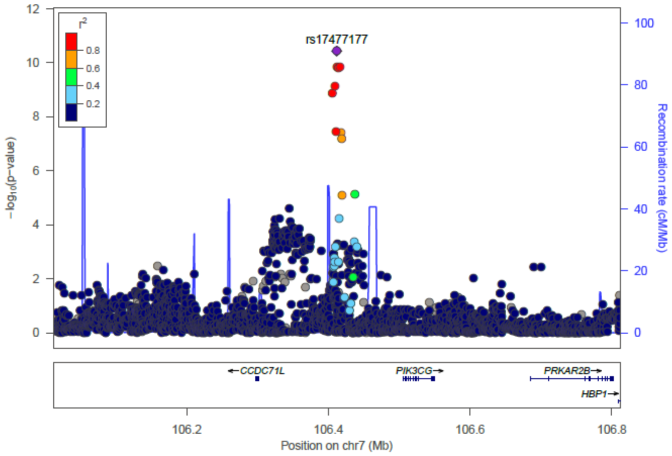
rs17477177
r2
−log10(p−value)
Recombination rate (cM/Mb)
CCDC71L
PIK3CG
PRKAR2B
HBP1
Position on chr7 (Mb)

N

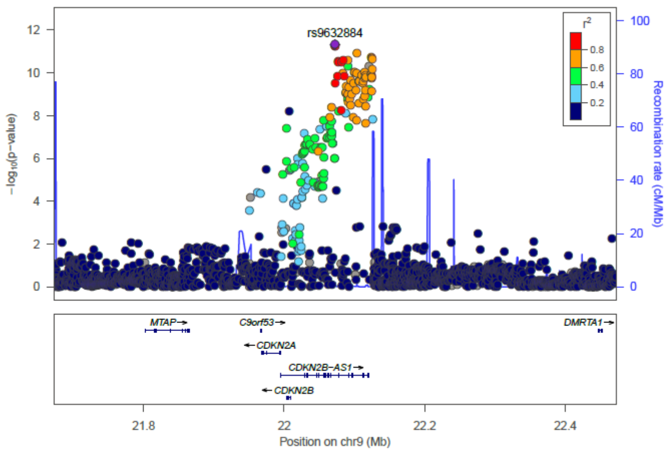
rs9632884
r2
−log10(p−value)
Recombination rate (cM/Mb)
MTAP
C9orf53
CDKN2A
CDKN2B−AS1
CDKN2B
DMRTA1
Position on chr9 (Mb)

O

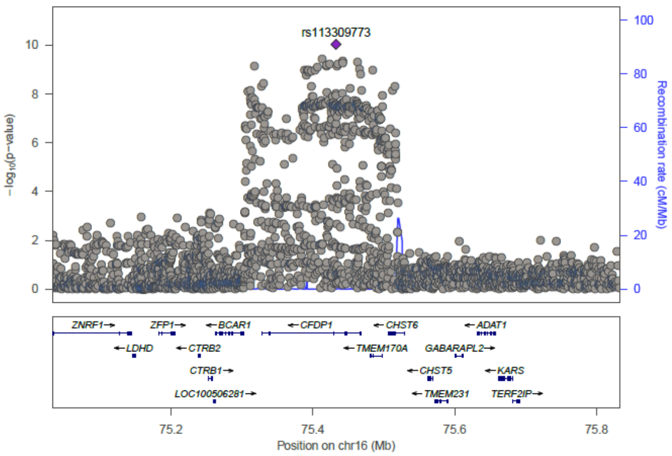
rs113309773
−log10(p−value)
Recombination rate (cM/Mb)
ZNRF1
ZFP1
BCAR1
CFDP1
CHST6
ADAT1
LDHD
CTRB2
TMEM170A
GABARAPL2
CTRB1
CHST5
KARS
LOC100506281
TMEM231
TERF2IP
Position on chr16 (Mb)

P

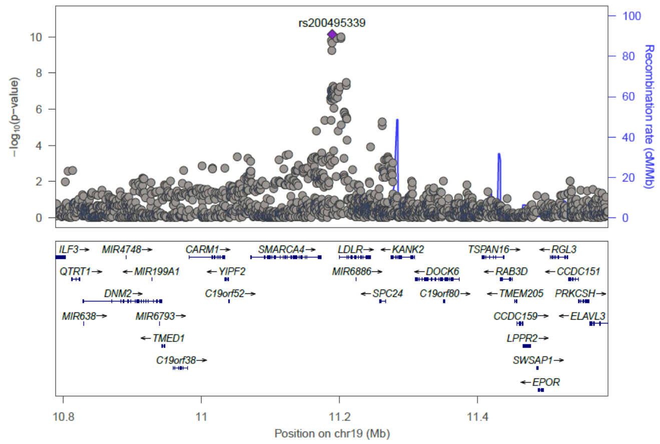
rs200495339
−log10(p−value)
Recombination rate (cM/Mb)
ILF3
MIR4748
CARM1
SMARCA4
LDLR
KANK2
TSPAN16
RGL3
QTRT1
MIR199A1
YIPF2
MIR6886
DOCK6
RAB3D
CCDC151
DNM2
C19orf52
SPC24
C19orf80
TMEM205
PRKCSH
MIR638
MIR6793
CCDC159
ELAVL3
TMED1
LPPR2
C19orf38
SWSAP1
EPOR
Position on chr19 (Mb)

Supplementary Figure 3. Forest plots of SNPs significantly associated with cIMT or plaque

cIMT

Chr1:208953176 (INDEL)

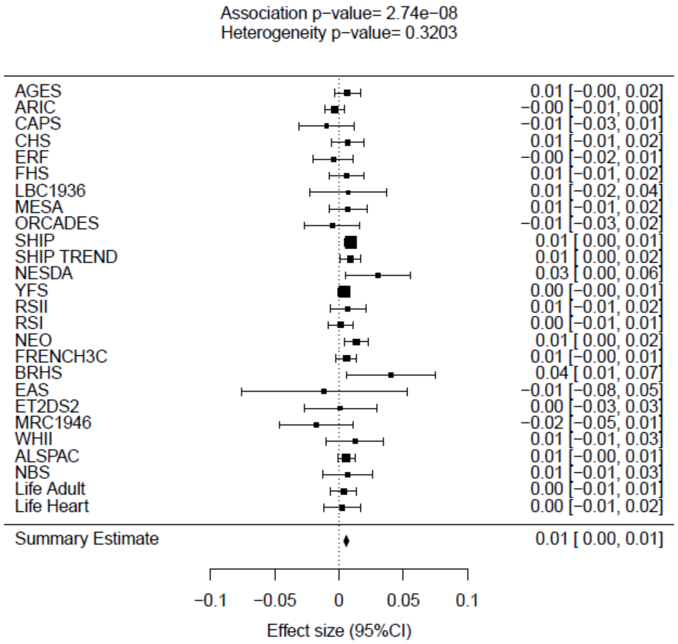

Chr5:81637916 (SNP)

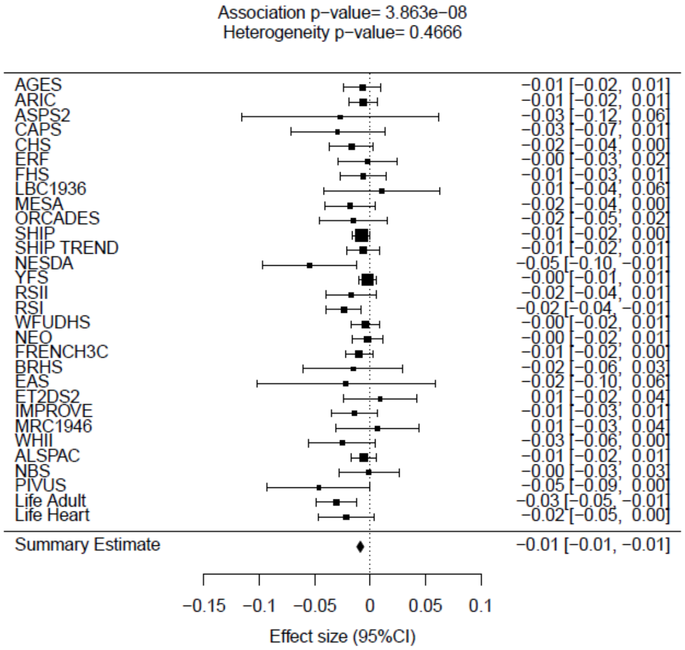

Chr6:143608968 (SNP)

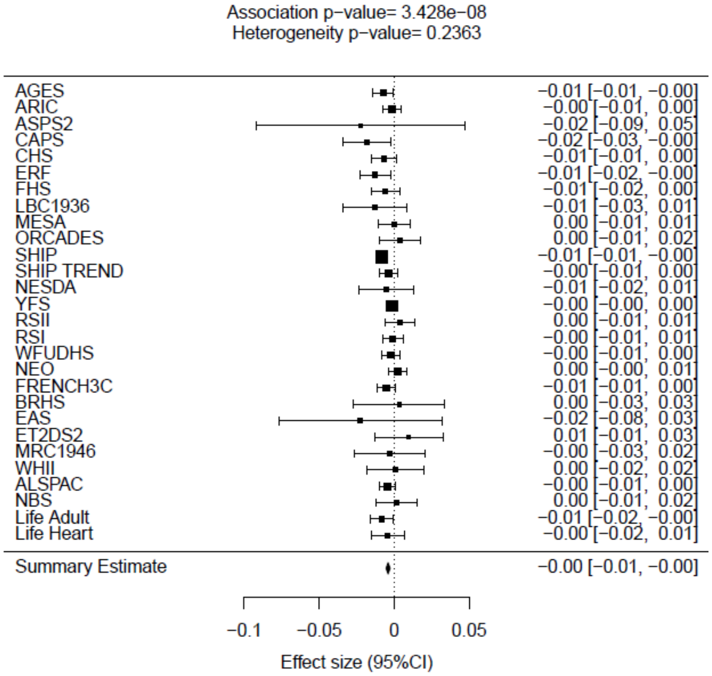

Chr7:106416467 (SNP)

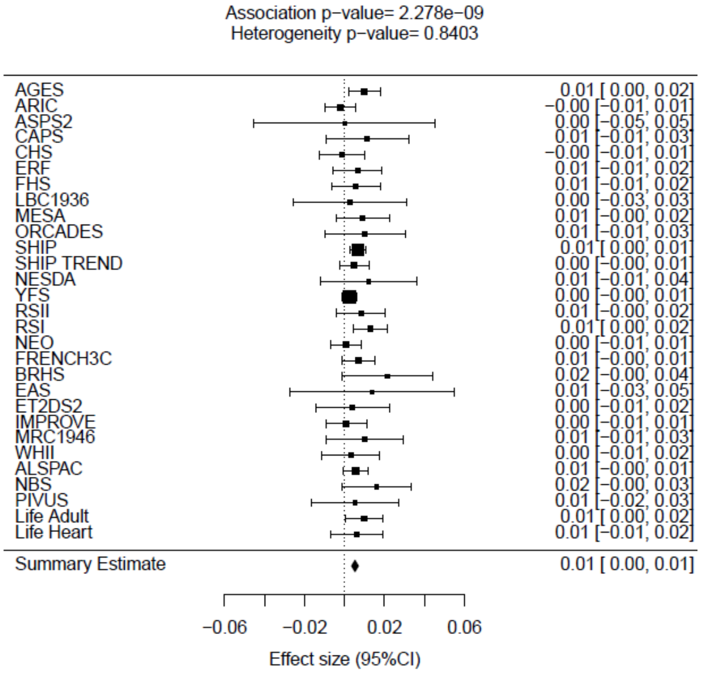

Chr8:6486033 (SNP)

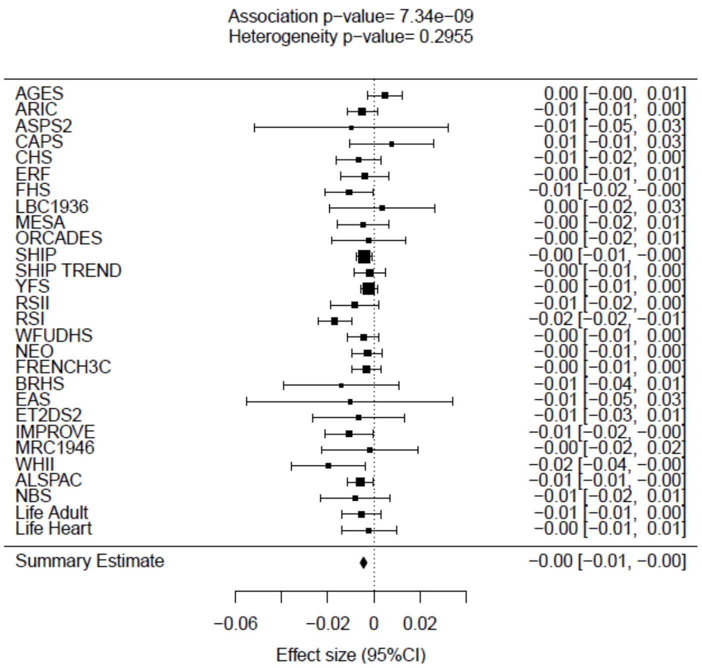

Chr8:8205010 (SNP)

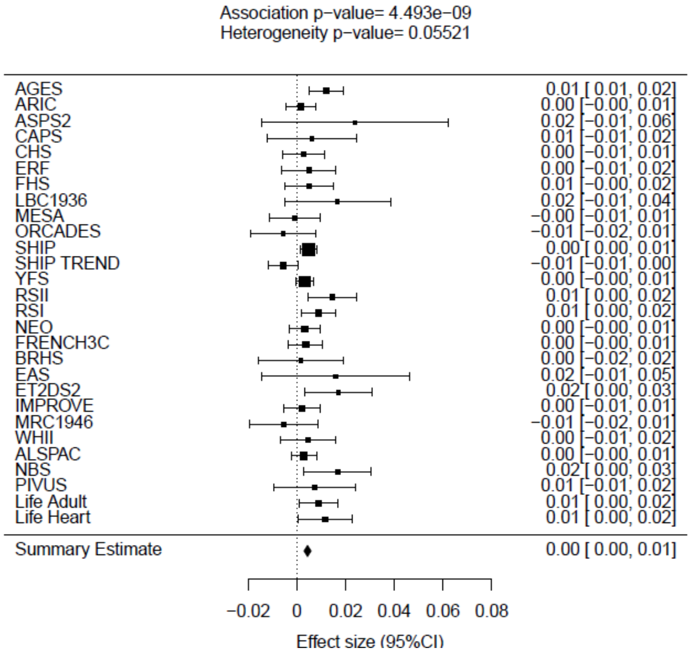

Chr8:10606223 (INDEL)

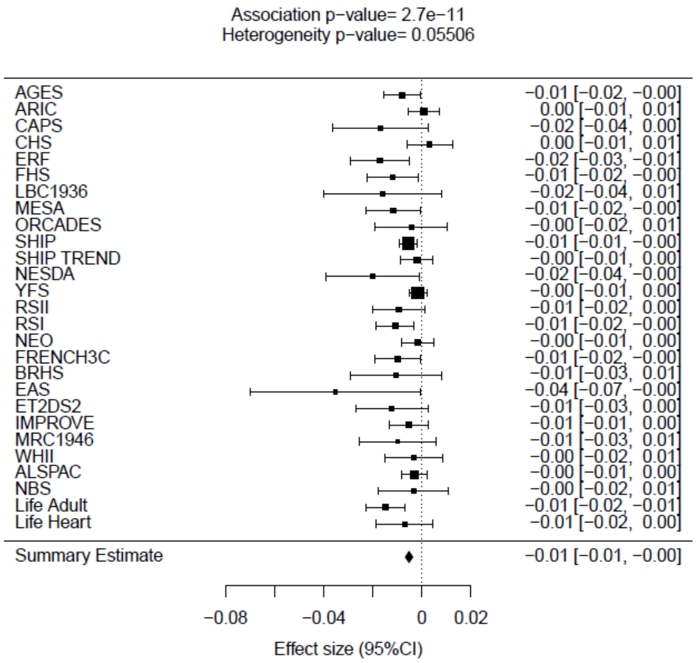

Chr8:123401537 (INDEL)

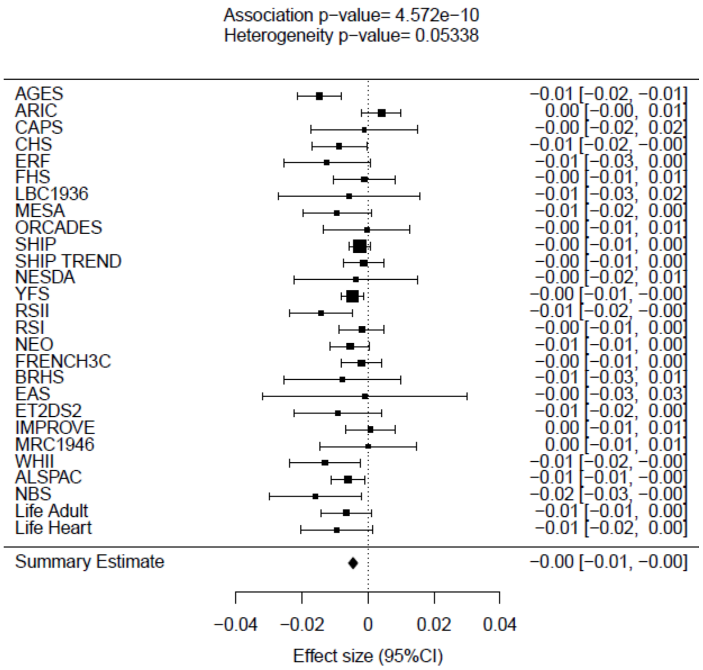

Chr10:114410998 (SNP)

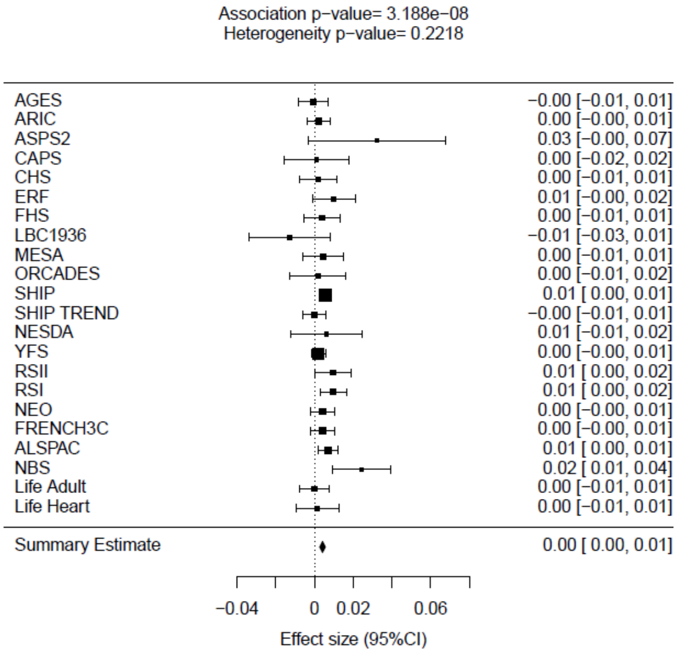

Chr16:88966667 (SNP)

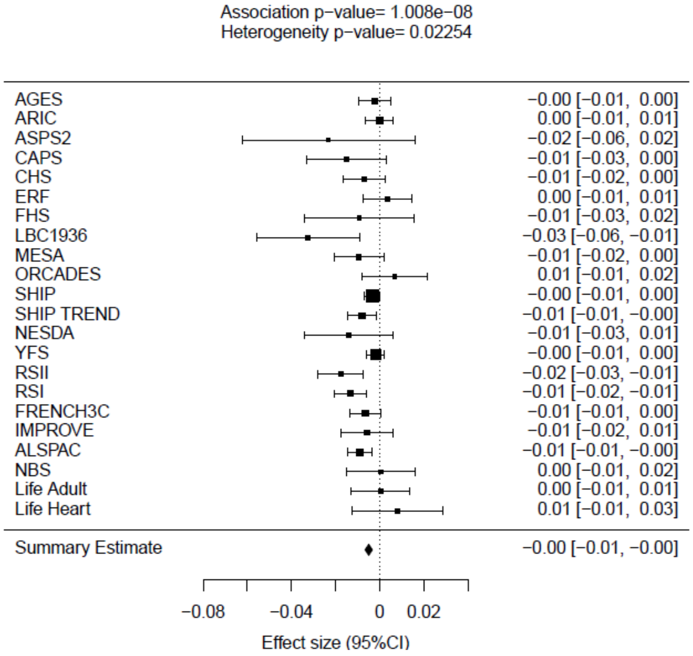

Chr19:45412079 (SNP)

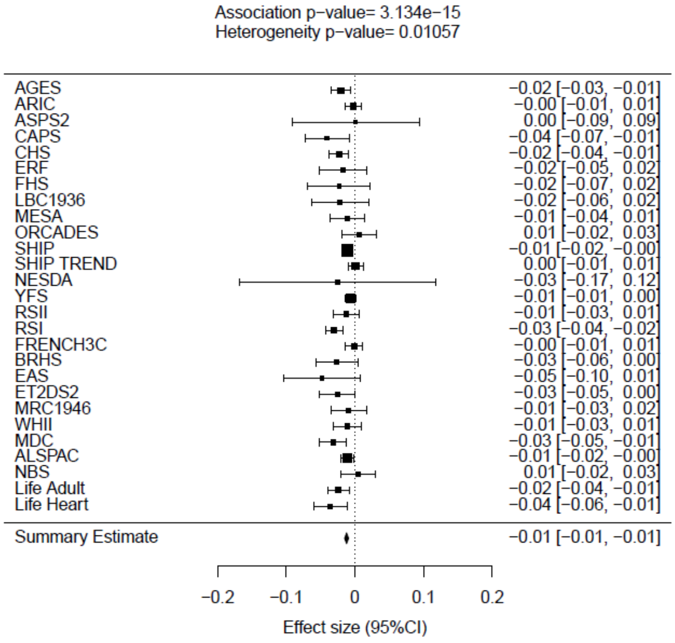

Plaque

Chr4:148395284 (INDEL)

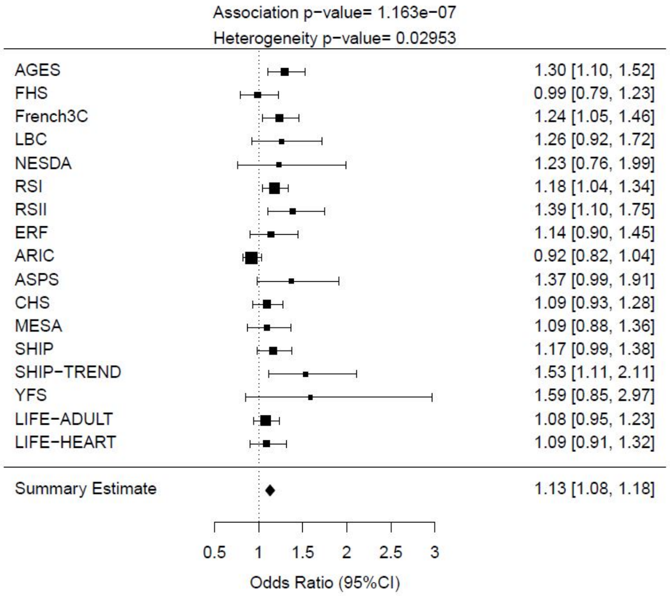

Chr7:106411858 (INDEL)

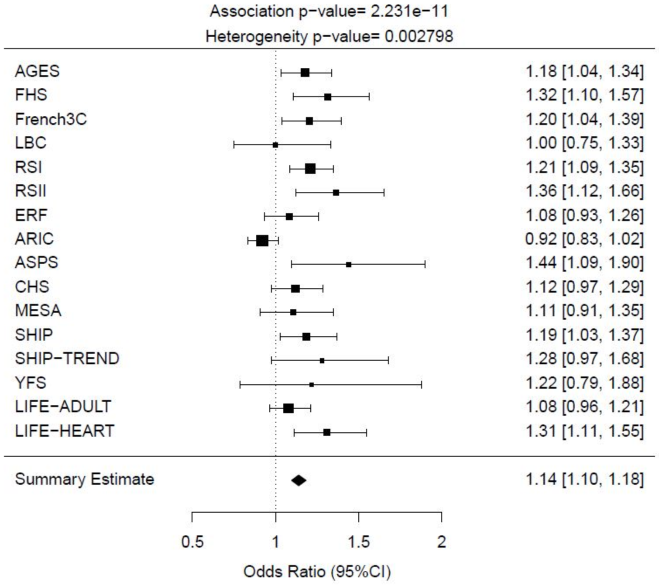

Chr9:22072301 (SNP)

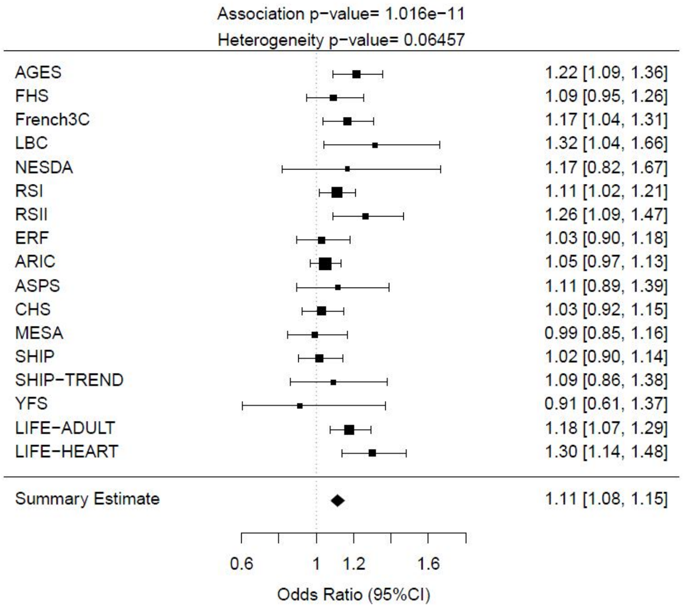

Chr16:75432688 (INDEL)

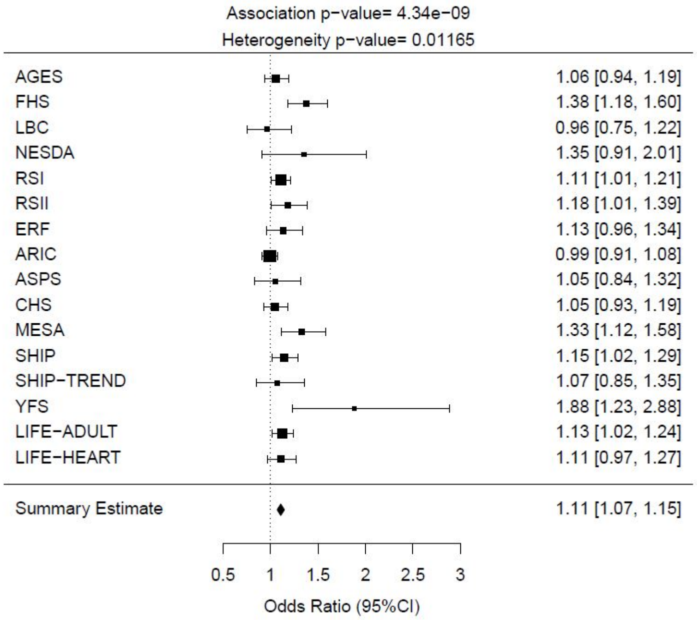

Chr19: 11189298 (INDEL)

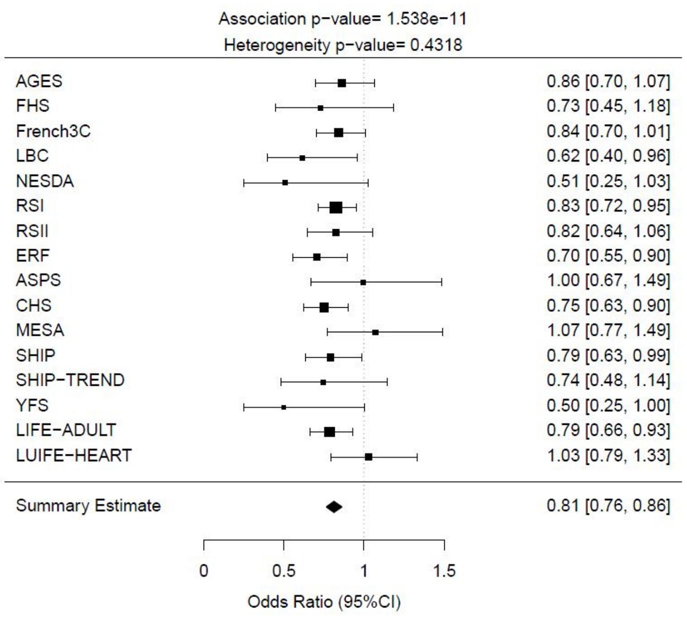

Supplementary Figure 4. Regional plot surrounding the *PINX1* locus for cIMT. The top panel shows the P values for SNPs association with cIMT. The middle panel shows the overlaps of SNPs with annotations included in the combined model in fGWAS. The bottom panel shows the fitted empirical prior probability based on the fGWAS combined model. The SNP association shown in purple (chr8:10659406; P = 6.4 x 10^{-11}) falls within active transcription (REMC.coreHMM.FAT_ADIP_DR_MSC.5_TxWk) in Adipose Derived Mesenchymal Stem Cells and a DNaseI-hypersensitive site (ENCODE.DHS_Maurano.CMK.DS12393) leading the model to assign a higher probability compared to the index SNP (index SNP chr8:10606223:INDEL; P = 1.3 x 10^{-12}).

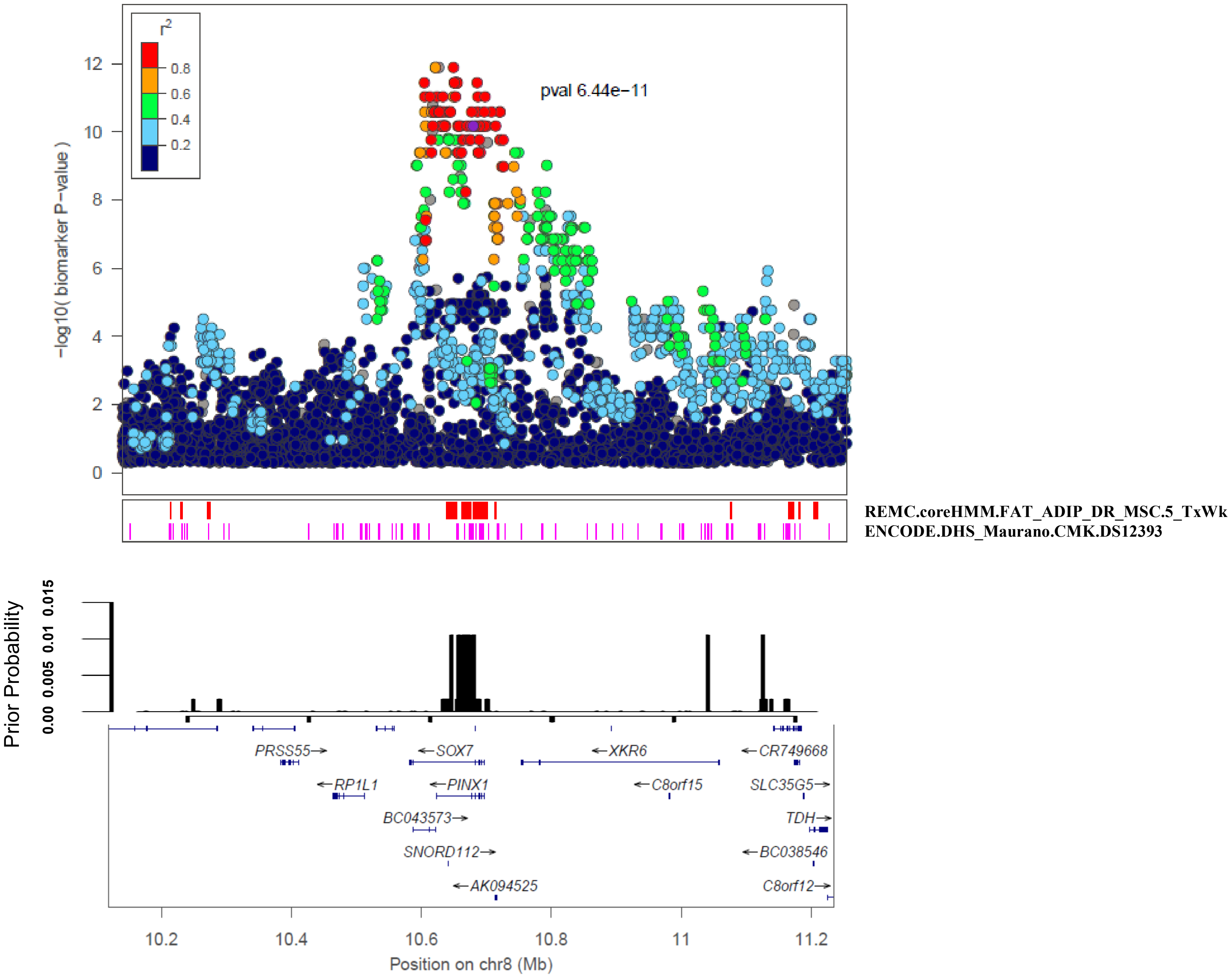

Supplementary Figure 5. Pairwise colocalization of GWAS SNPs and tissue eQTLs.

Colocalization results for cIMT (A) and AOR (B) and MAM (C) eQTLs

CCDC71L

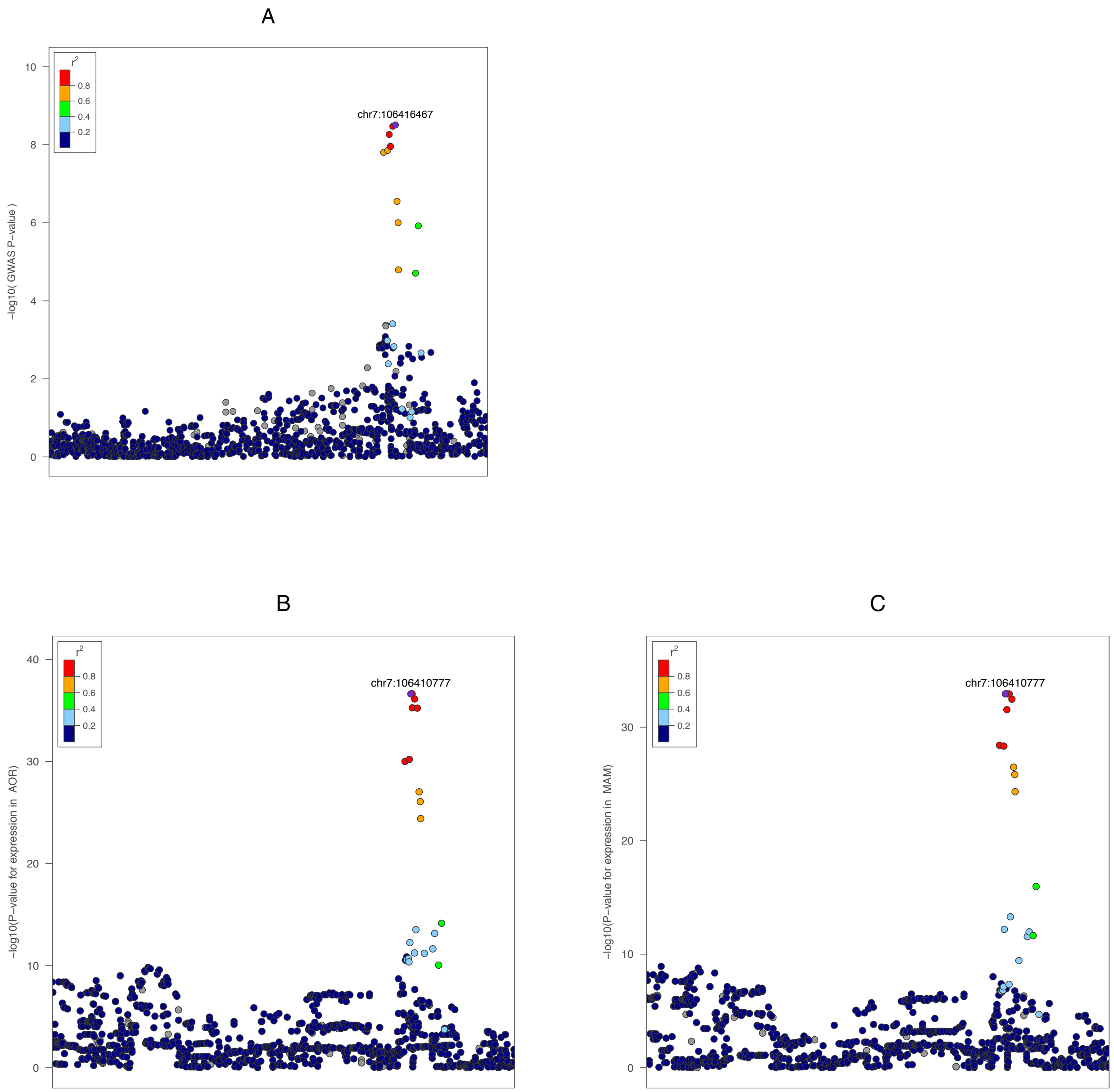

PRKAR2B

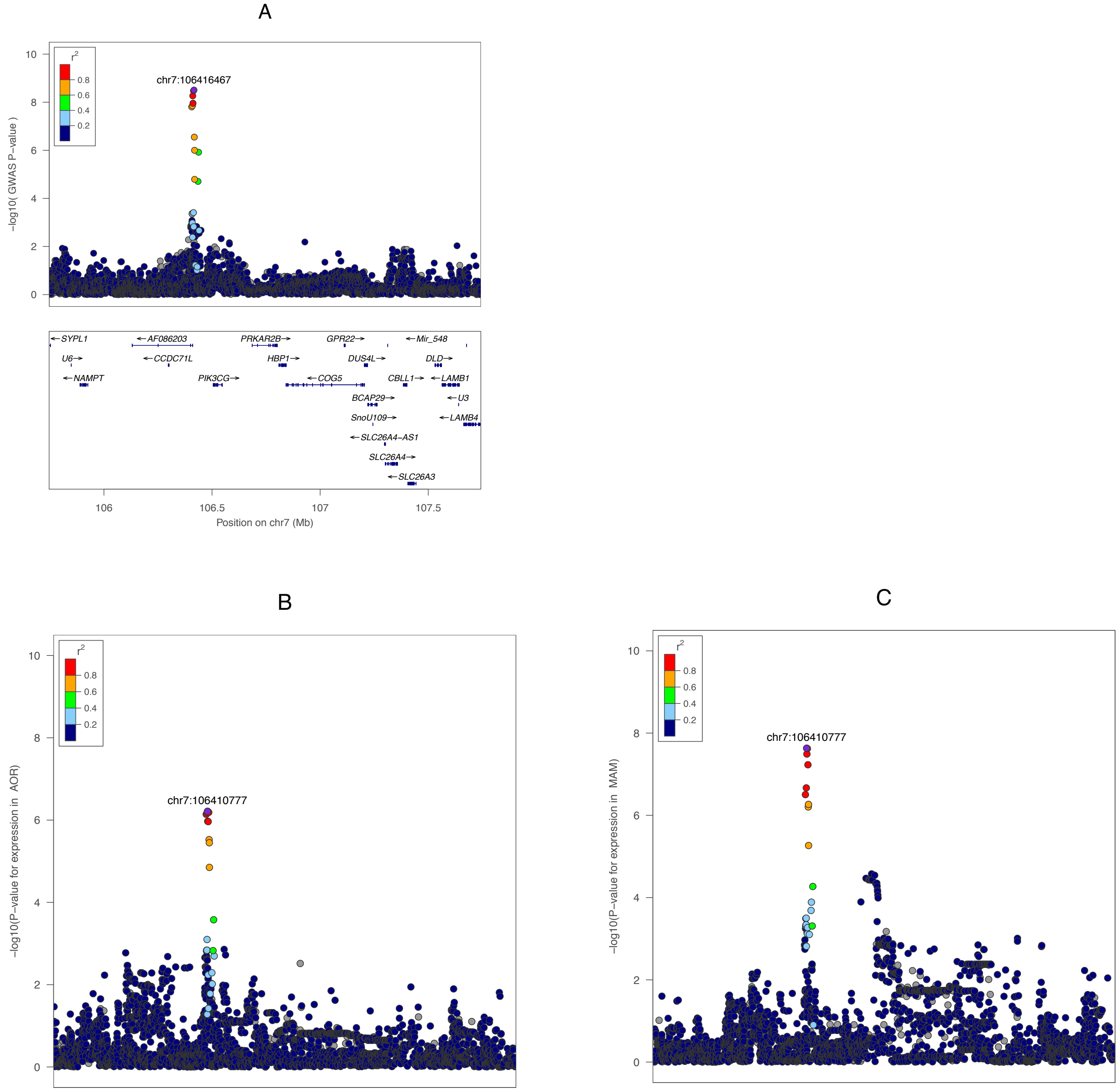

A
chr7:106416467
r2
0.8
0.6
0.4
0.2
−log10(GWAS P−value)
SYPL1
AF086203
PRKAR2B
GPR22
Mir_548
U6
CCDC71L
HBP1
DUS4L
DLD
NAMPT
PIK3CG
COG5
CBLL1
LAMB1
BCAP29
U3
SnoU109
LAMB4
SLC26A4−AS1
SLC26A4
SLC26A3
106
106.5
107
107.5
Position on chr7 (Mb)
B
chr7:106410777
−log10(P−value for expression in AOR)
C
chr7:106410777
−log10(P−value for expression in MAM)

ADAMTS9

A

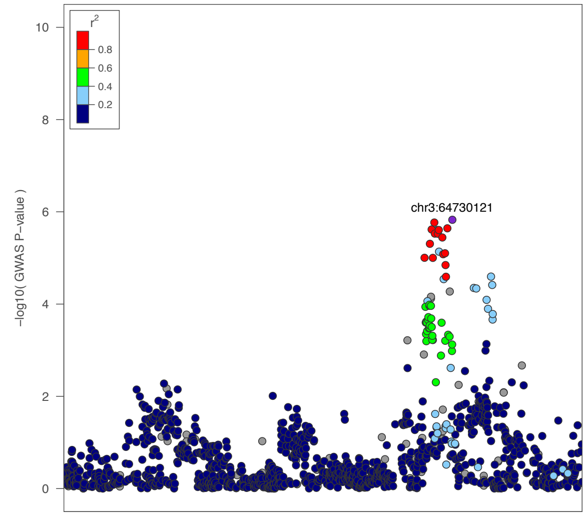
r2
0.8
0.6
0.4
0.2
chr3:64730121
−log10(GWAS P−value)
10
8
6
4
2
0

B

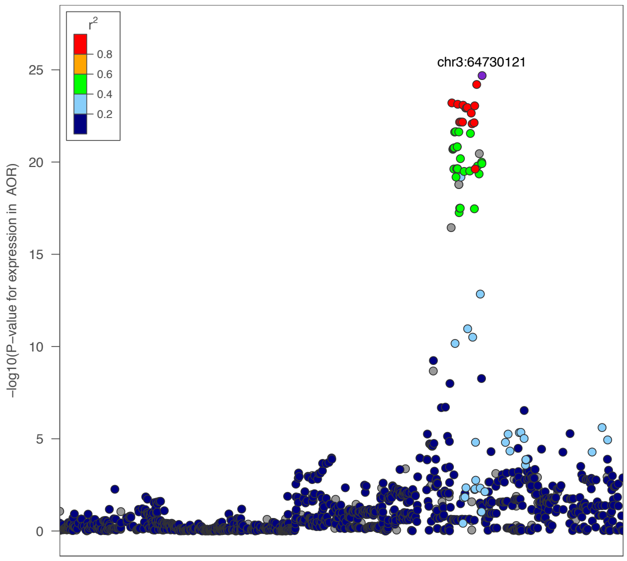
r2
0.8
0.6
0.4
0.2
chr3:64730121
−log10(P−value for expression in AOR)
25
20
15
10
5
0

C

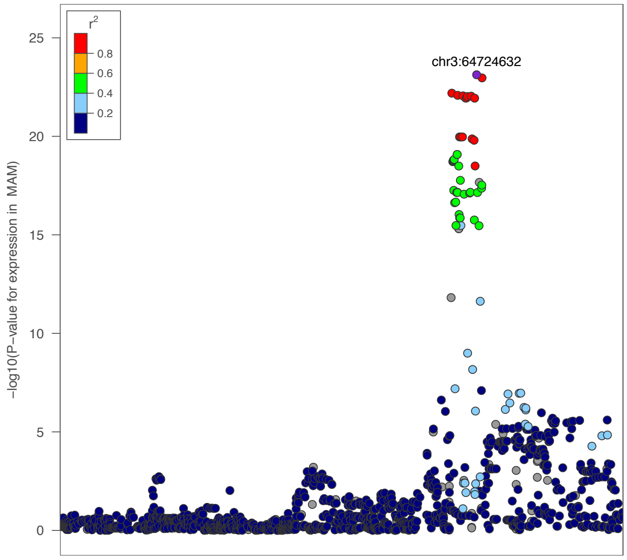
r2
0.8
0.6
0.4
0.2
chr3:64724632
−log10(P−value for expression in MAM)
25
20
15
10
5
0

Colocalization of cIMT (A), Aorta eQTL (B)

LOXL4

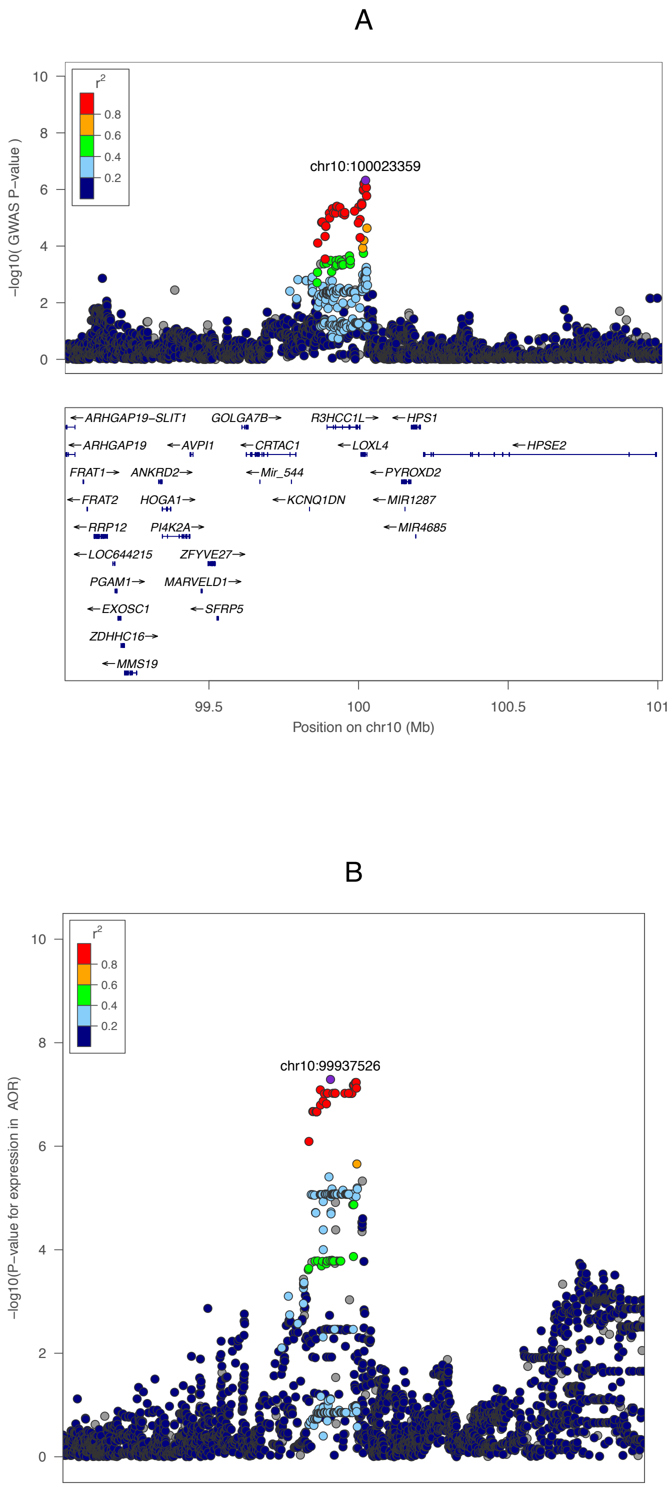

Colocalization of plaque (A), Aorta eQTL (B), and MAM eQTL (C)

KIAA1462

A

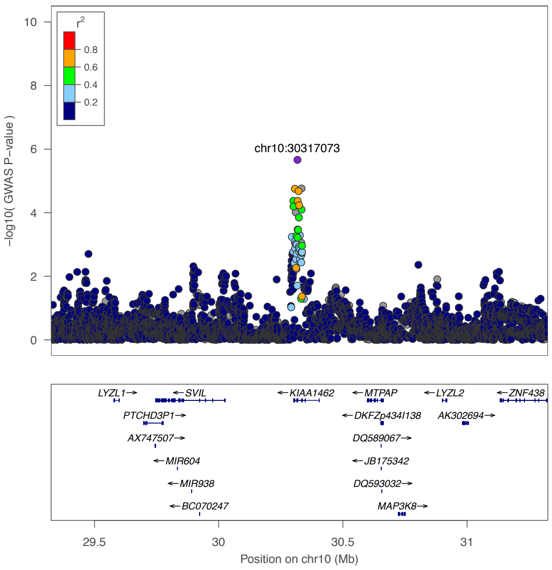

B

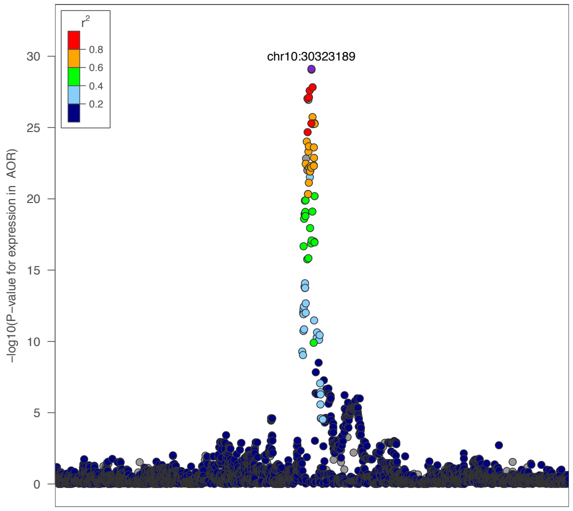

C

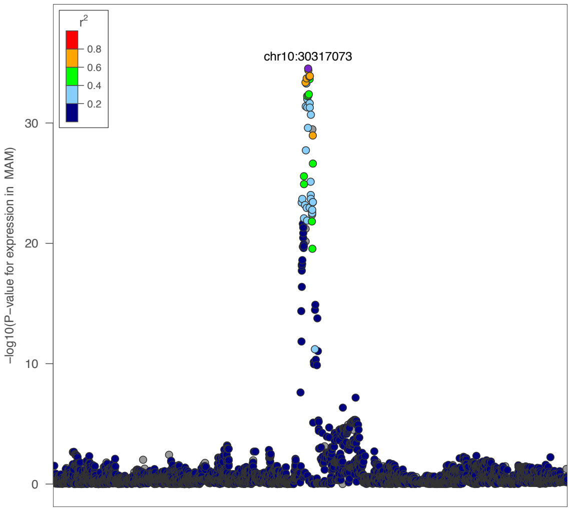

CCDC71L

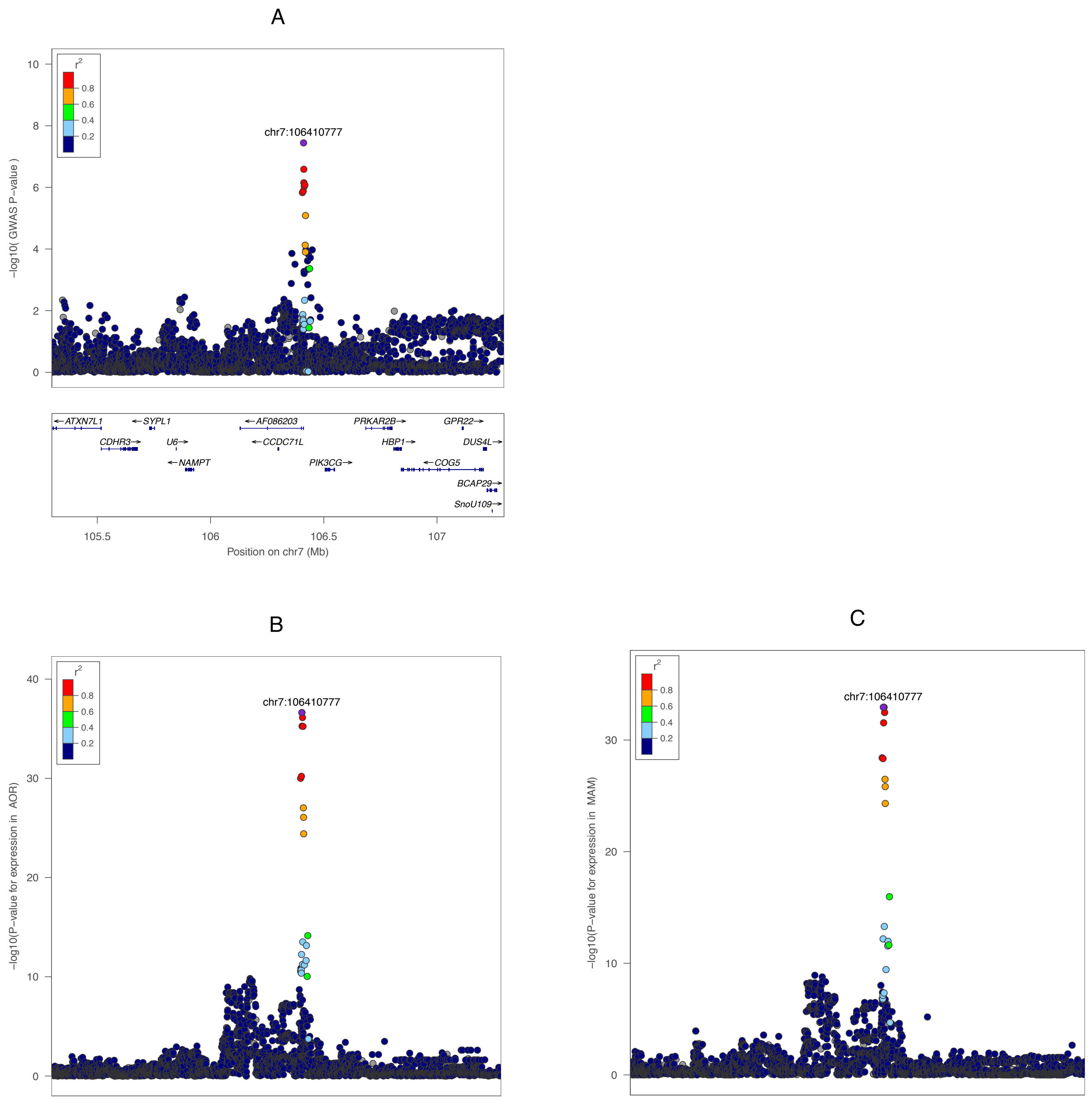
A
chr7:106410777
r2
0.8
0.6
0.4
0.2
−log10(GWAS P−value)
ATXN7L1
SYPL1
AF086203
PRKAR2B
GPR22
CDHR3
U6
CCDC71L
HBP1
DUS4L
NAMPT
PIK3CG
COG5
BCAP29
SnoU109
105.5
106
106.5
107
Position on chr7 (Mb)
B
chr7:106410777
−log10(P−value for expression in AOR)
C
chr7:106410777
−log10(P−value for expression in MAM)

PRKAR2B

A

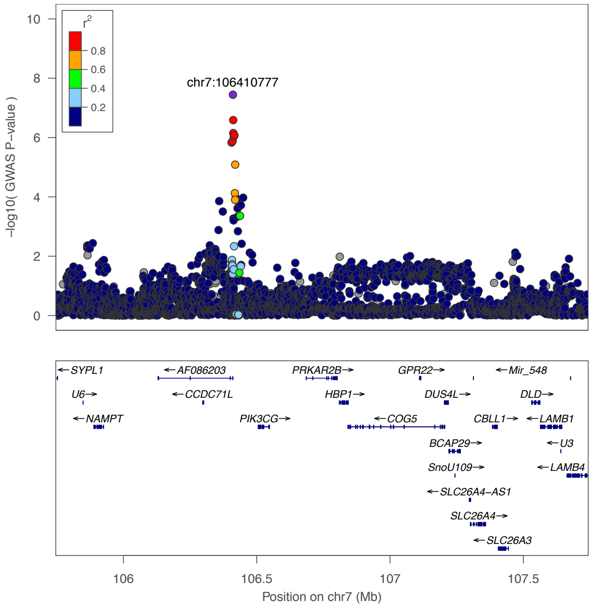
chr7:106410777
r2
0.8
0.6
0.4
0.2
−log10(GWAS P−value)
SYPL1
AF086203
PRKAR2B
GPR22
Mir_548
U6
CCDC71L
HBP1
DUS4L
DLD
NAMPT
PIK3CG
COG5
CBLL1
LAMB1
BCAP29
U3
SnoU109
LAMB4
SLC26A4−AS1
SLC26A4
SLC26A3
106
106.5
107
107.5
Position on chr7 (Mb)

B

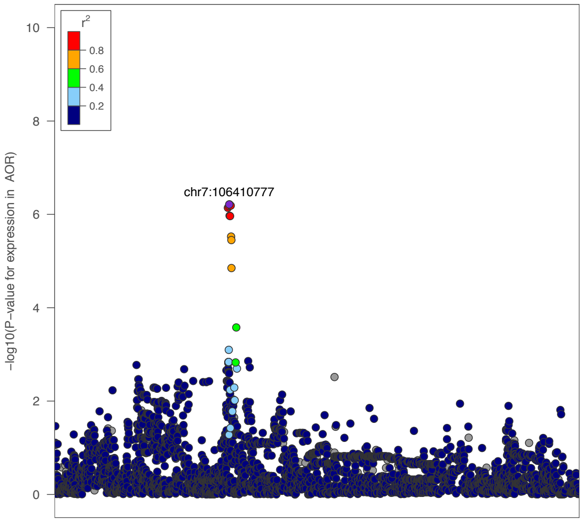
chr7:106410777
r2
0.8
0.6
0.4
0.2
−log10(P−value for expression in AOR)

C

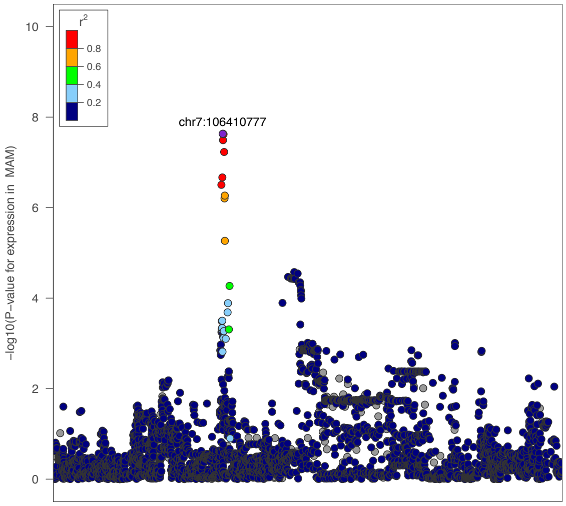
chr7:106410777
r2
0.8
0.6
0.4
0.2
−log10(P−value for expression in MAM)

Supplementary Figure 6. Pairwise colocalization results for genes identified for cIMT and plaque GWAS meta-analysis with GWAS of coronary heart disease from CARDIOGRAMplusC4D and stroke subtypes from METASTROKE consortium. Posterior probability of colocalization is shown with red being a probability of colocalization of the same SNP and blue the high probability of no colocalization of the same SNP with clinical outcomes of coronary heart disease and stroke, or subtypes. Tissue expression in AOR (aortic root), MAM (mammary artery); stroke subtypes are IS (ischaemic stroke), CE (cardio-embolic stroke), LVD (large vessel disease), SVD (small vessel disease) as defined in Methods.

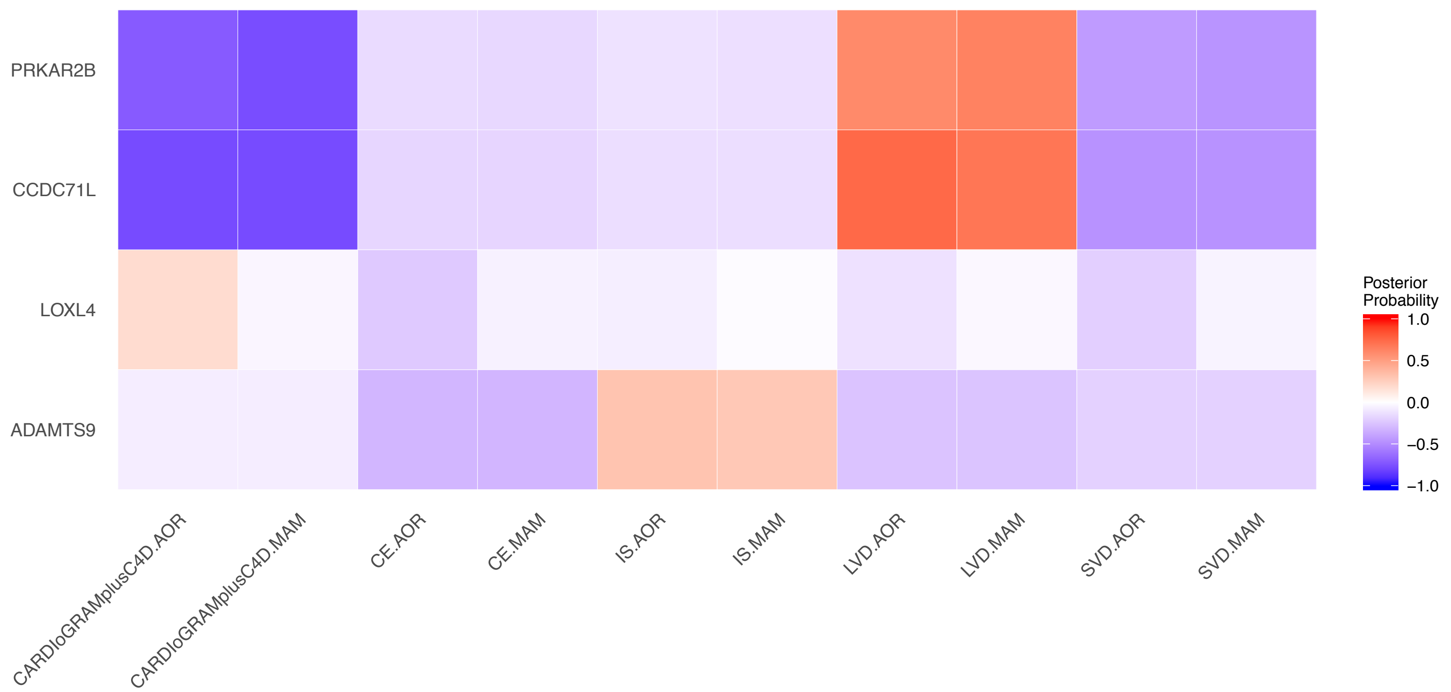

Supplementary Figure 7. Expression of *KIAA1462* gene in MAM, and plaque and CHD GWAS. Each dot represents effect size estimates from associations of gene expression of *KIAA1462* in MAM (x-axis) against associations of plaque (blue) or CHD (red) for SNPs at the *KIAA1462* locus. The diamond around the dots represent the SNP with the strongest association across all datasets (10:30317073, rs9337951).

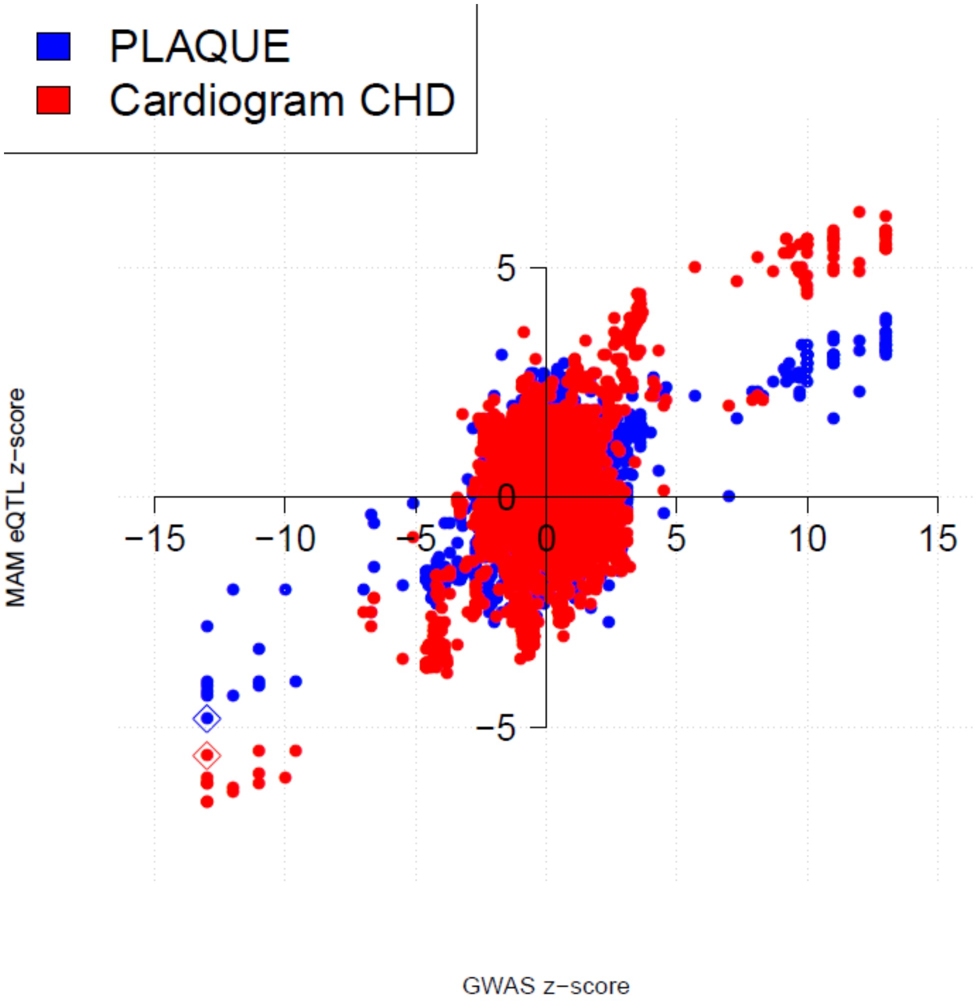

Supplementary Figure 8. GO term enrichment analysis of the protein-coding genes identified as nearest to or in LD with the variants for cIMT and plaque (Table 1 and Supplementary Table 5), and genes identified in colocalization analyses (Table 2). GO term enrichment was performed using the VLAD tool to find GO terms that are significantly enriched in the list of genes identified compared to the human proteome. The larger the size of box, the more significantly enriched the term is; the significance is represented by the p-value after the term name. k = number of proteins in the input list that are annotated to the term, K = number of human proteins in total that are annotated to the term. The lines (edges) connecting the nodes in the graph represent the relationships between the terms. A purple line with a solid, diamond-shaped tip represents a "part-of" relationship between terms; a blue line with a hollow arrow tip represents an "is-a" relationship between the terms. A black line with a solid arrow tip indicates that several nodes in a multi-step path are not being displayed in order to simplify the graphic. See VLAD tool (https://www.ncbi.nlm.nih.gov/pmc/articles/PMC4602057/).

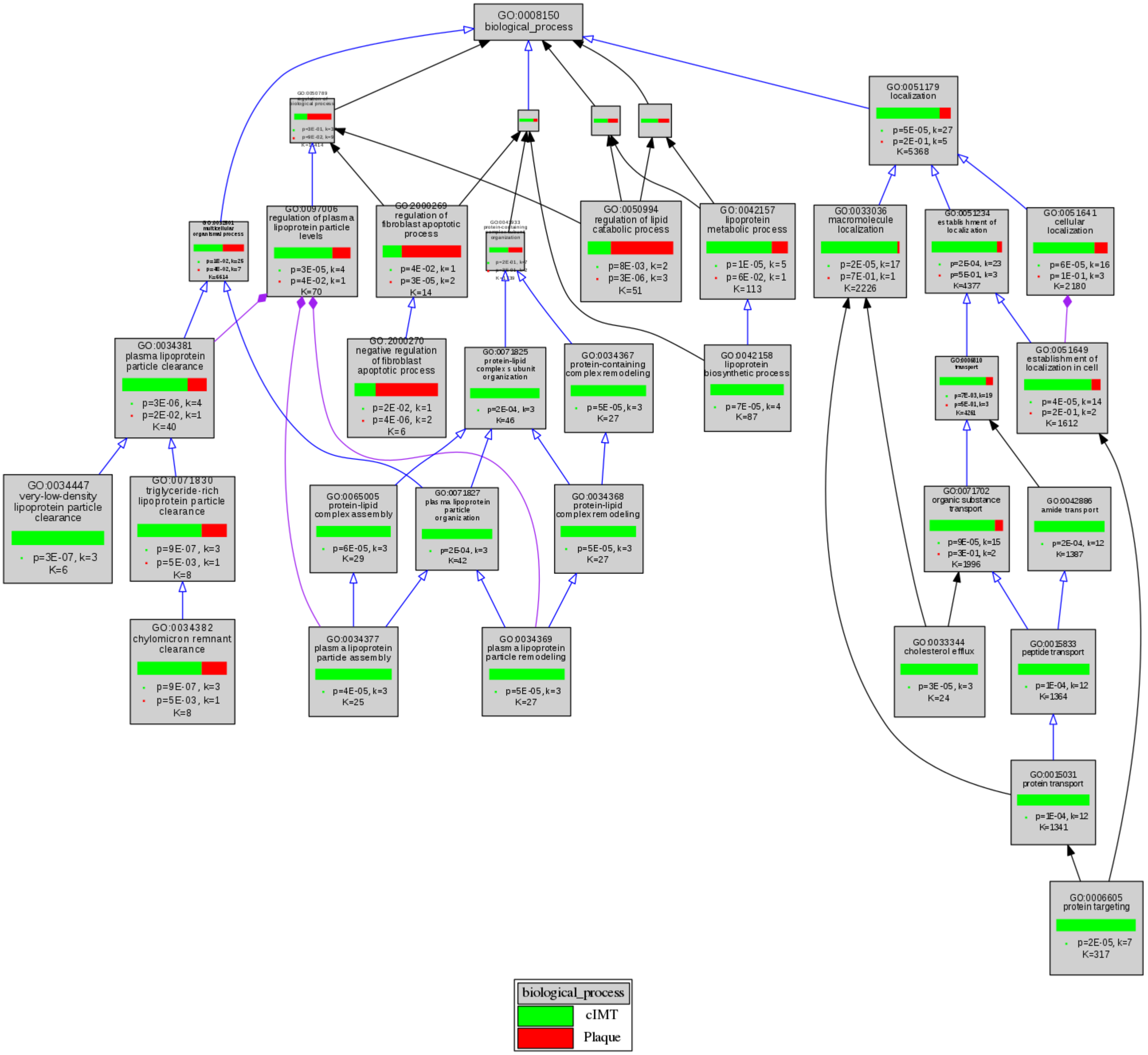

Supplementary Tables

Supplementary Table 1. Characteristics of the study samples

Study	Sex (F/M)	Sample size cIMT	Age (years) mean (SD)	cIMT (mean, SD)	Sample size plaque	N plaques	Plaque frequency
AGES	1771/1297	3068	76.4 (5.4)	1.13 (0.16)	3053	2043	0.67
ARIC	4596/4067	8663	54.3 (5.7)	0.76 (0.18)	8857	1626	0.18
ASPS-FAM	176/127	303	65.5 (11.0)	0.84 (0.32)			
ASPS-FAM	439/334		65.9 (8.0)		773	490	0.63
CAPS	443/443	886	48.9 (13.3)	0.73 (0.19)			
CHS	1265/1975	3239	72.3 (5.4)	1.03 (0.20)	3125	2069	0.66
DHS	1 12/25	915	61.4 (9.5)	0.66 (0.12)			
ERF	1507/1214	2270	48.7 (14.4)	0.82 (0.20)	2443	1218	0.50
FHS	1601/1403	3004	58.5 (9.7)	1.02 (0.18)	3008	530	0.18
3C-Dijon	1581/937	2518	72.6 (4.0)	0.69 (0.11)	2473	1218	0.49
LBC1936	363/396	759	72.8 (0.8)	0.85 (0.19)	759	220	0.29
MESA	1309/1198	2500	62.6 (10.3)	0.87 (0.20	2492	393	0.16
NEO	2949/2726	5675	56.0 (5.9)	1.00 (0.16)			
NESDA	368/204	572	44.7 (12.2)	0.66 (0.16)	572	86	0.15
ORCADES	763/1128	1914	53.7 (14.9)	0.50 (0.10)			
RS I	2968/1978	4946	69.0 (8.8)	1.02 (0.21)	4910	2920	0.59
RS II	1079/901	1980	64.7 (7.9)	0.99 (0.17)	2016	1509	0.75
SHIP	1838/1781	3619	53.3 (13.7)	0.85 (0.20)	3666	1989	0.54
SHIP-TREND	551/432	983	50.1 (13.7)	0.73 (0.17)	985	338	0.34
ALSPAC	3200/0	3200	47.9 (4.5)	0.55 (0.11)			
YFS	1106 /909	2015	37.7 (5.0)	0.66 (0.10)	2013	48	0.02
BRHS	0/889	889	78.7 (4.8)	0.79 (0.18)			
EAS	378/353	731	69.8 (5.6)	0.75 (0.18)			
ET2DS	423/445	868	68.9 (4.2)	0.94 (0.11)			
IMPROVE	1753/1636	3389	64.5 (1.9)	0.85 (0.07)			
LIFE-Adult	1677/1531	3208	59.1 (11.9)	0.76 (0.15)	4534	2726	0.60
LIFE-Heart	684/1240	1924	62.5 (11.0)	0.78 (0.15)	2755	2117	0.77
MDC	1093/1050	2142	57.4 (6.0)	0.73 (0.144)			
MRC1946	655/603	1258	63.3 (1.1)	0.68 (0.18)			
NBS	281/268	549	57.8 (5.2)	0.86 (0.11)			
PIVUS	482/482	964	70.2 (0.2)	0.88 (0.16)			
WHII	508/1669	2177	60.8 (5.9)	0.77 (0.19)			

Supplementary Table 2. Study definition of carotid artery plaque

Study	Plaque definition	Reference (PMID)
AGES	Of the left and right carotid bifurcation and internal carotid artery the presence of atherosclerotic lesions was be quantified during the ultrasound examination. The most severe lesion per segment was assessed in a semi-quantitative manner as none, minimal, moderate and severe lesion.	17351290
ARIC	Presence of a lesion defined by abnormal arterial wall thickness, shape, or texture. Acoustic shadowing defined as a reduction in amplitude of echoes caused by intervening structures with high attenuation.	9180252
ASPS	Plaque was graded according to the most severe visible changes in the CCA and ICA as 0, normal; 1,vessel wall thickening >1 mm; 2, minimal plaque (<2 mm); 3, moderate plaque (2 to 3 mm); 4, severe plaque (>3 mm), and 5, lumen completely obstructed	7800110;10408549
CHS	Largest focal lesion classified by surface characteristics, echogenicity, and texture. A discernible focal widening of the wall relative to adjacent segments with or without protrusion into the lumen was described according to the following criteria: surface—smooth, mildly irregular, markedly irregular, or ulcerated; morphology—homogeneous or heterogeneous; and densi- ty—hypodense, isodense, hyperdense, or calcified.	1669507
ERF	The cIMT and the carotid bifurcation were evaluated for the presence (yes/no) of atherosclerotic lesions on both the near and far walls of the carotid arteries. Plaques were defined as a focal widening relative to adjacent segments, with protrusion into the lumen composed either of only calcified deposits or a combination of calcification and noncalcified material. The size or extent of the lesions was not quantified.	15845033
FHS	Defined by carotid stenosis of 25% or greater.	
LBC1936	We measured carotid flow velocities, maximum stenosis affecting the internal carotid artery/bulb/CCA Plaques were defined by carotid stenosis of 25% or greater.	22253310
Life Adult & Life Heart	Carotid artery plaque was defined as echogenic thickening of intimal reflection that extends into the arterial lumen at least 0.5 mm or 50 % of the surrounding CCA-IMT value or an intimal + medial thickness of >1.5 mm. Plaque presence was documented as 'present' or 'absent' for the common part and bulb of the right and left carotid artery, respectively.	26362881
MESA	Defined by carotid stenosis of 25% or greater.	12397006
NESDA	Widening of the intimal and medial layers relative to adjacent segments, with the area of focal increased thickness ≥ 1.10 mm	18763692;19065144 21745125
RS-I	Plaques were defined as a focal widening relative to adjacent segments, with protrusion into the lumen composed either of only calcified deposits or a combination of calcification and noncalcified material.	19728115
RS-II	Plaques were defined as a focal widening relative to adjacent segments, with protrusion into the lumen composed either of only calcified deposits or a combination of calcification and noncalcified material.	19728115
SHIP/SHIP-TREND	Atherosclerotic plaques were defined as a focal thickening of the vessel wall with protrusion into the vessel lumen relative to adjacent segments or as a localized roughness with increased echogenicity.	11565448; 20167617
3C-Dijon	The presence of plaques was defined as localized echo structures encroaching into the vessel lumen for which the distance between the media–adventitia interface and the internal side of the lesion was >1 mm on the common carotid arteries, the carotid bifurcations, and the internal carotid arteries.	14598854; 18063810

YFS	The far and near walls of the left common carotid artery and carotid bulb area were scanned for the presence of atherosclerotic plaque, defined as a distinct area of the vessel wall protruding into the lumen >50% of the adjacent intima–media layer.	18263651

Supplementary Table 3. Study-specific genotyping, quality control, imputation and analysis

		Sample quality control		Imputation		Association analysis			
Study	Genotyping array	Call rate	Other exclusions	software	Reference panel	software	covariates	λGC cIMT	λGC plaque
AGES	Illumina 370CNV BeadChip	< 0.97	HWE p-value < 10^-6	MaCH	1,000 Genomes Phase I integrated release March 2012 (v3)	ProbAbel	age,sex	0.984	1.173
ARIC	Affymetrix 6.0	<0.95	HWE p-value <10^-5, MAF<0.01	IMPUTE2	1,000 Genomes Phase I integrated release March 2012 (v3)	FAST	age, sex, region, 10 PCs	1.017	1.028
ASPS	Illumina Human610-Quad BeadChip	< 98%	HWE p-value 1<10^-6, MAF<0.01, sex mismatch, cryptic relatedness	IMPUTE2	1,000 Genomes Phase I integrated release March 2012 (v3)	plink	age, sex	1.000	1.013
ASPS-Fam	Affymetrix Genome-Wide Human SNP Array 6.0	< 98%	HWE p-value 5<10^-6, MAF<0.05, sex mismatch, cryptic relatedness	IMPUTE2	1,000 Genomes Phase I integrated release March 2012 (v3)	GWAF	age, sex	1.005	1.018
CAPS	Affymetrix 6.0	<0.90	HWE p-value 1<10^-6, MAF<0.01, sex mismatch, cryptic relatedness	SHAPEIT v2.778 (phasing) and IMPUTE2 2.3.0 (imputation)	1,000 Genomes Phase I integrated release March 2012 (v3)	plink2	age,sex,pc1,pc2,pc3,pc4	1.008	1.019
CHS	Illumina 370CNV BeadChip + Illumina IBC iSELECT	<0.95	HWE p-value <10^-5	MACH/miniMACH (whites) & IMPUTE v 2.2.2 (African Americans)	1,000 Genomes Phase I integrated release March 2012 (v3)	R	age, sex, clinic	1.026	NA
ERF	Illumina 318/370 K, Affymetrix 250 K, and Illumina 6 K	95%	HWE < 10^-6, MAF < 0.01, snp call rate < 98%, Mendelian errors	miniMACH	1,000 Genomes Phase I integrated release March 2012 (v3)	R, GenABEL, ProbABEL	age, sex (family structure)	0.997	1.057
FHS	Affymetrix 500K	<0.95	HWE p-value <10^-6	MaCH/mimimac	1,000 Genomes Phase I integrated release March 2012 (v3)	R, GEE for dichotomous, LME for continuous trait	Age at the examination cycle 6, sex, and 10 PCs	1.007	NA
3C-Dijon	Illumina Human610 Quad	≤0.95		minimac	1,000 Genomes Phase I integrated release March 2012 (v3)	ProbAbel/R	Age_baseline, Sex, PC1, PC2, PC3, PC4	1.018	1.006

LBC	Illumina 610 Quad V1	<95%	HWE P <10-3, relatedness, MAF<1%, gender mismatch, SNP call rate <98%	MiniMAC	1,000 Genomes Phase I integrated release March 2012 (v3)	Mach2qtl	age, sex, 4 PCs	1.025	1.001
MESA	Affymetrix 6.0	< 0.95	HWE p-value < 10^-8; MAF < 0.005	IMPUTE2	1,000 Genomes Phase I integrated release March 2012 (v3)	SNPTEST V2.4	age, gender, site, and 4 PCs	1.017	1.014
NEO	Illumina HumanCoreExome-24v1_A Beadchip	<0.98	HWE P < 1e-5	IMPUTE2	1,000 Genomes Phase I integrated release March 2012 (v3)	ProBABEL	age, sex, 4 PCs	1.009	1.03
NESDA	Affymetrix 6.0 907K	0.9	heterozygosity abs(PLINK F)>0.1; sex mismatch; unexpected relatedness	minimac	1,000 Genomes Phase I integrated release March 2012 (v3)	SNPTEST	age, sex	1.023	NA
ORCADES	Illumina HumanHap300	0.98	HWE p-value <10^-6	MaCH	1,000 Genomes Phase I integrated release March 2012 (v3)	ProbAbel	age, sex, 3 PCs	1.001	0.992
RS I	Illumina 550K	0.975	HWE p-value <10^-6, MAF<0.001	MaCH/minimac	1,000 Genomes Phase I integrated release March 2012 (v3)	ProbAbel		1.013	NA
RS II	Illumina 550K	0.975	HWE p-value <10^-6, MAF<0.001	MaCH/minimac	1,000 Genomes Phase I integrated release March 2012 (v3)	ProbAbel		0.996	NA
SHIP	Affymetrix 6.0	0.92	duplicates, reported/genotyped gender mismatch	IMPUTE2	1,000 Genomes Phase I integrated release March 2012 (v3)	Quicktest	age, sex	1.012	NA
SHIP-TREND	Illumina Human Omni 2.5	0.94	duplicates, reported/genotyped gender mismatch	IMPUTE2	1,000 Genomes Phase I integrated release March 2012 (v3)	Quicktest	age, sex	0.99	NA
DHS	Affymetrix Genome-Wide Human SNP Array 5.0	0.95	Excluded sample failures, sex discordance, unclear/unexpected sibling relationships (based on IBD)	IMPUTE2	Phase I 1000G Integrated Variant Set version 2, cosmopolitan (integrated) reference panel	SOLAR 6.3.6	age, sex, first two admixture PCs	1.054	NA
YFS	Illumina Human670-QuadCustom	0.95	HWE p-value <10^-6, MAF<0.01	SHAPEIT v1 and IMPUTE2	1,000 Genomes Phase 1 CEU haplotype set	SNPTEST		1.016	1.007
ALSPAC	Illumina human660W-quad array	0.95	HWE p-value <10^-6, MAF<0.01, gender mismatch (X	IMPUTE2	1,000 Genomes Phase I integrated release March 2012 (v3)	SNPTEST V2.5	age, 10 PCs	1.013	NA

			chromosome heterozygosity or extreme autosomal heterozygosity); unexpected relatedness (Pi hat of more than 0.125 which is expected to correspond to roughly 12.5% alleles shared IBD); population stratification (determined by IBS).						
BRHS	MetaboChip	0.95	HWE p-value <10^-6, MAF<0.01, gender mismatch and relatedness	MaCH/minimac	1,000 Genomes Phase I integrated release March 2012 (v3)	snpStats	age, sex	0.996	NA
EAS	MetaboChip	0.95	HWE p-value <10^-6, MAF<0.01, gender mismatch and relatedness	MaCH/minimac	1,000 Genomes Phase I integrated release March 2012 (v3)	snpStats	age, sex	1.012	NA
ET2DS	MetaboChip	0.95	HWE p-value <10^-6, MAF<0.01, gender mismatch and relatedness	MaCH/minimac	1,000 Genomes Phase I integrated release March 2012 (v3)	snpStats	age, sex	0.99	NA
IMPROVE	Combined Metabochip and Immunochip	0.95	HWE p-value <5^-7, MAF<0.01, gender mismatch and relatedness	MACH1/Minimac	1,000 Genomes Phase I integrated release March 2012 (v3)	Plink	age, sex, 3 PC	1.054	NA
LIFE-Adult	Affymetrix Axiom	< 0.97	HWE p-value <10^-6, MAF=0, gender mismatch and relatedness	IMPUTE2	1,000 Genomes Phase I integrated release March 2012 (v3)	SNPTEST v2.5	age, sex	1.016	1.007
LIFE-Heart	Affymetrix Axiom	< 0.97	HWE p-value <10^-6, MAF=0, gender mismatch and relatedness	IMPUTE2	1,000 Genomes Phase I integrated release March 2012 (v3)	SNPTEST v2.5	age, sex	1.010	1.004
MRC1946	MetaboChip	0.95	HWE p-value <10^-6, MAF<0.01, gender mismatch and relatedness	MaCH/minimac	1,000 Genomes Phase 1 CEU haplotype set	snpStats	age, sex	0.995	NA

MDC	MetaboChip	0.95	sex-mismatches, relatedness; SNP QC: callrate<95%; HWE p-value <10^-6	N/A	NA	Plink	age, sex	1.017	NA
NBS	Illumina HumanHapCNV 370-Duo BeadChip	0.95	HWE p-value <10^-4, MAF<0.01, gender mismatch and relatedness	IMPUTE2	1000 Genomes phase1 v3 together with Genome of The Netherlands (GoNL) release 5	snpStats	age, sex	0.999	NA
PIVUS	MetaboChip	0.95	HWE p-value <10^-6, MAF<0.01, gender mismatch and relatedness	IMPUTE2	HapMap2	Plink	age, sex	1.013	NA
WHII	MetaboChip	0.95	HWE p-value <10^-6, MAF<0.01, gender mismatch and relatedness	MaCH/minimac	1,000 Genomes Phase I integrated release March 2012 (v3)	snpStats	age, sex	0.995	NA

NA, not available

Supplementary Table 4. Conditional analysis using GCTA for cIMT and plaque

Trait	SNP	chr:position	RefAllele	Freq	Beta	SE	*p*	n	freq_geno	bJ	bJ_se	pJ	LD_r
cIMT	rs2912064	8:6488710	T	0.36	-0.0041	0.0007	1.58E-08	76752	0.64775	-0.0041	0.0007	4.30E-09	0.00333
cIMT	rs11785239	8:8205010	T	0.65	0.0044	0.0008	4.48E-09	59522.7	0.35388	0.00441	0.0008	3.47E-08	0

Columns are: freq_geno: frequency of the effect allele in the reference sample;
bJ, bJ_se, pJ: effect size, standard error and p-value from a joint analysis of all the selected SNPs;
LD_r: LD correlation between the SNP i and SNP i + 1 for the SNPs on the list.

Supplementary Table 5.Loci associated with cIMT and plaque GWAS at p< 10^{-7} among individuals of European ancestry

SNP	chr:position	Nearest Coding Gene	Alleles Effect/ Other	Effect allele frequency	Beta (SE)	*p*	N
**cIMT**							
rs515135	chr2:21286057	*APOB*	T/C	0.18	-0.0487 (0.0009)	8.2 x 10-8	65,428
rs139302128	chr2:242594226	*ATG4B*	T/C	0.03	0.0487 (0.0091)	7.6 x 10-8	17,713
**Plaque**							
rs4779614	chr15:33540117	*TMCO5B*	T/C	0.35	0.0869 (0.0171)	4.0 x 10-7	48,434
rs259140	chr7:89624347	*STEAP2-AS1*	T/G	0.30	0.0889 (0.0177)	5.1 x 10-7	47,862

Supplementary Table 6. Nearest gene from top GWAS SNP of cIMT and plaque, and best colocalizing gene intersecting a region of 200kb from the listed SNP using STARNET tissue eQTL. Main association are SNPs with p-value < $5x10^{-8}$ (Table 1); Suggestive association are $p<10^{-7}$ (Table S5). Best colocalizing gene is the gene with the largest posterior probability of colocalization in the joint GWAS and eQTL analysis. ngenes is the max number of genes considered across the tissues in a region of +/-200kb around the GWAS SNP.

SNP	chr:position	Nearest Coding Genes	Max number of genes across tissues considerd in region (+/- 200Kb from GWAS SNP)	Max number of genes across tissues suggestive of association (PP3 + PP4 ³ 50%) with both GWAS and STARNET eQTLs in region (+/- 200Kb from GWAS SNP)	Best co-localizing gene (eQTL STARNET data, PP4)
**cIMT**					
**Main**					
rs13225723	chr7:106416467	*PIK3CG, CCDC71L, PRKAR2B*	20	6	*CCDC71L* (AOR, PP4=97.48)
rs148147734	chr8: 123401537	*ZHX2*	10	4	*HAS2* (AOR, PP4=53.58)
rs6907215	chr6:143608968	*AIG1*	18	7	*ENSG00000217648* (MAM, PP4=47.77)
rs7412	chr19: 45412079	*APOE*	61	28	*APOE* (SF, PP4=24.84)
rs2912063	chr8: 6486033	*MCPH1, ANGPT2*	5	4	*ENSG00000249898* (AOR, PP4=27.36)
rs844396	chr16:88966667	*CBFA2T3*	41	23	*RPL13* (AOR, PP4=10.5)
rs200482500	chr8: 10606223	*PINX1, SOX7*	13	10	*ENSG00000258724** (LIV, PP4=4.29)
rs11785239	chr8:8205010	*SGK223****	8	7	*PPP1R3B* (VAF, PP4=3.29)
rs201648240	chr1:208953176:INDEL		11	4	*TRAF3IP3* (MAM, PP4=3.74)
rs11196033	chr10: 114410998	*VTI1A*	10	4	*VTI1A* (LIV, PP4=2.44)
rs224904	chr5:81637916	*ATP6AP1L, ATG10*	15	10	*ENSG00000248870* (MAM, PP4=0.81)
**Suggestive**					
rs515135	chr2:21286057	*APOB*	18	7	*HS1BP3* (MAM, PP4=5.27)
rs139302128	chr2:242594226	*ATG4B*	29	0	*ENSG00000237940* (Blood, PP4=2.26)
**Plaque**					
**Main**					
rs113309773	16:75432686	*CFDP1- TMEM170A*	19	10	*BCAR1* (AOR, PP4=26)
rs11413744	4:148395284:INDEL	*EDNRA*	9	3	*EDNRA* (AOR, PP4=49)
rs17477177	7:106411858	*PIK3CG*	21	6	*CCDC71L* (AOR, PP4=97)
rs200495339	19:11189298:INDEL	*LDLR*	69	21	*ELOF1* (MAM, PP4=5.5)
rs9632884	9:22072301	*9p21*	6	6	*CDKN2B* (AOR, PP4=39)
**Suggestive**					
rs259140	7:89624347	*STEAP2-AS1*	7	6	*CLDN12* (LIV, PP4=13)

rs4779614	15:33540117	*TMCO5B*	15	6	*GREM1* (SKLM, PP4=21)

**ENSG00000258724* is a long transcript that has exons derived from both *PINX1* and *SOX7*, the encoded protein is 440 aa long, with approx. 310 aa derived from *SOX7* exons (*SOX7* is 388aa) and 130 aa derived from *PINX1* exons. UniProt has included ENSG00000258724 within the SOX7, describing it as an alternative spliced product Q9BT81-2. Although it has aa sequence from both genes.

** *LDLR* has an eQTL only in LIV, with p-value 1.73e-05. However, there is no evidence of colocalization with GWAS (PP4=0.5%)

****SGK223*, *SCEL* not covered in STARNET

Supplementary Table 7. Multiple trait colocalization of cIMT and plaque with AOR/MAM eQTLs (STARNET) and CHD (CARDIoGRAMPlusC4D), or stroke subtypes (MEGASTROKE) with probability of colocalization across three traits ≥ 75%.

Gene.name	Chr	Start-Stop	Data a	Data b	Data c	N snps	PPA abc	Best snp abc	Min p_value data a	Min p_value data b	Min p_value data c	Min p-value SNP data a	Min p-value SNP data b	Min p-value SNP data c
ADAMTS9	3	63588304-65587494	AOR	cIMT	Stroke AS	4415	0.80	rs17676309	2.06E-25	1.49E-06	1.11E-05	rs17676309	rs17676309	rs28546794
ADAMTS9	3	63588304-65587494	MAM	cIMT	Stroke AS	4415	0.77	rs17676309	7.48E-24	1.49E-06	1.11E-05	rs6775974	rs17676309	rs28546794
ADAMTS9-AS1	3	63561280-65561009	AOR	cIMT	Stroke AS	4411	0.80	rs17676309	4.03E-15	1.49E-06	1.11E-05	rs17676309	rs17676309	rs28546794
ADAMTS9-AS2	3	63841395-65833136	MAM	cIMT	Stroke AS	4533	0.76	rs17676309	6.41E-13	1.49E-06	1.11E-05	rs17676309	rs17676309	rs28546794
CCDC71L	7	105299372-107298840	AOR	Plaque	Stroke LAS	3929	0.81	rs17477177	2.37E-37	3.71E-11	0.000362	rs12705390	rs17477177	rs17398575
CCDC71L	7	105299372-107298840	AOR	cIMT	Stroke LAS	3910	0.81	rs12705390	2.37E-37	3.12E-09	0.000362	rs12705390	rs13225723	rs17398575
CCDC71L	7	105299372-107298840	MAM	cIMT	Stroke LAS	3910	0.80	rs12705390	1.17E-33	3.12E-09	0.000362	rs12705390	rs13225723	rs17398575
CCDC71L	7	105299372-107298840	MAM	Plaque	Stroke LAS	3929	0.79	rs17477177	1.17E-33	3.71E-11	0.000362	rs12705390	rs17477177	rs17398575
CDH13	16	82245780-84245226	AOR	cIMT	CHD	7999	0.94	16:83045790	6.81E-71	1.71E-05	2.11E-06	16:83045790	16:83045790	16:83045790
CDH13	16	82245780-84245226	MAM	cIMT	CHD	7999	0.94	16:83045790	2.47E-46	1.71E-05	2.11E-06	16:83045790	16:83045790	16:83045790
EDNRA	4	147434590-149433978	AOR	Plaque	Stroke LAS	2955	0.8	rs17612742	3.16E-05	5.68E-08	1.05E-06	rs6841581	rs10305839	rs17612742
ENSG00000232821.1	7	18153930-20152801	MAM	Plaque	CHD	4244	0.84	7:19049388	3.00E-14	0.00024	8.00E-11	7:19049388	7:18843808	7:19049388
ENSG00000232821.1	7	18153930-20152801	MAM	Plaque	Stroke AS	3874	0.84	rs2107595	2.97E-14	0.00024	3.59E-11	rs2107595	rs2520343	rs2107595
ENSG00000232821.1	7	18153930-20152801	MAM	Plaque	Stroke IS	3878	0.84	rs2107595	2.97E-14	0.00024	2.33E-11	rs2107595	rs2520343	rs2107595
ENSG00000232821.1	7	18153930-20152801	MAM	Plaque	Stroke LAS	3894	0.84	rs2107595	2.97E-14	0.00024	1.44E-13	rs2107595	rs2520343	rs2107595
ENSG00000260228.1	16	82832549-84832129	AOR	cIMT	CHD	8667	0.94	16:83045790	8.36E-18	1.71E-05	2.11E-06	16:83045790	16:83045790	16:83045790
ENSG00000260523.1	16	82832949-84832129	AOR	cIMT	CHD	8664	0.94	16:83045790	7.83E-30	1.71E-05	2.11E-06	16:83045790	16:83045790	16:83045790
ENSG00000260523.1	16	82832949-84832129	MAM	cIMT	CHD	8664	0.94	16:83045790	4.54E-17	1.71E-05	2.11E-06	16:83045790	16:83045790	16:83045790
ENSG00000260788.1	16	82780756-84780613	AOR	cIMT	CHD	8774	0.94	16:83045790	1.15E-33	1.71E-05	2.11E-06	16:83045790	16:83045790	16:83045790
ENSG00000260788.1	16	82780756-84780613	MAM	cIMT	CHD	8774	0.94	16:83045790	5.39E-20	1.71E-05	2.11E-06	16:83045790	16:83045790	16:83045790

ENSG00000260832.1	16	82006107-84004822	AOR	cIMT	CHD	7626	0.94	16:83045790	3.95E-36	1.71E-05	2.11E-06	16:83045790	16:83045790	16:83045790
ENSG00000260832.1	16	82006107-84004822	MAM	cIMT	CHD	7626	0.94	16:83045790	5.54E-17	1.71E-05	2.11E-06	16:83045790	16:83045790	16:83045790
ENSG00000261103.1	16	82748233-84747503	AOR	cIMT	CHD	8809	0.94	16:83045790	9.70E-19	1.71E-05	2.11E-06	16:83045790	16:83045790	16:83045790
ENSG00000261410.1	16	82425634-84423396	AOR	cIMT	CHD	8403	0.94	16:83045790	1.62E-18	1.71E-05	2.11E-06	16:83045790	16:83045790	16:83045790
ENSG00000261410.1	16	82425634-84423396	MAM	cIMT	CHD	8403	0.93	16:83045790	2.39E-12	1.71E-05	2.11E-06	16:83017777	16:83045790	16:83045790
KIAA1462	10	29325616-31325032	MAM	Plaque	CHD	6180	0.83	10:30321598	3.00E-35	4.10E-06	4.40E-11	10:30317073	10:30317073	10:30323892
KIAA1462	10	29325616-31325032	MAM	cIMT	CHD	6222	0.84	10:30323892	3.00E-35	1.29E-06	4.41E-11	10:30317073	10:30333622	10:30323892
PRKAR2B	7	105745093-107743409	MAM	cIMT	Stroke LAS	3679	0.77	rs12705390	2.33E-08	3.12E-09	0.000362	rs12705390	rs13225723	rs17398575
PRKAR2B	7	105745093-107743409	AOR	Plaque	Stroke LAS	3697	0.76	rs17477177	6.12E-07	3.71E-11	0.000362	rs12705390	rs17477177	rs17398575
PRKAR2B	7	105745093-107743409	MAM	Plaque	Stroke LAS	3697	0.76	rs17477177	2.33E-08	3.71E-11	0.000362	rs12705390	rs17477177	rs17398575
PRKAR2B	7	105745093-107743409	AOR	cIMT	Stroke LAS	3679	0.76	rs12705390	6.12E-07	3.12E-09	0.000362	rs12705390	rs13225723	rs17398575
TWIST1	7	18157302-20154816	AOR	Plaque	CHD	4240	0.84	7:19049388	1.50E-10	0.00024	8.00E-11	7:19049388	7:18843808	7:19049388
TWIST1	7	18157302-20154816	MAM	Plaque	CHD	4240	0.84	7:19049388	1.60E-37	0.00024	8.00E-11	7:19049388	7:18843808	7:19049388
TWIST1	7	18157302-20154816	AOR	Plaque	Stroke AS	3870	0.84	rs2107595	1.46E-10	0.00024	3.59E-11	rs2107595	rs2520343	rs2107595
TWIST1	7	18157302-20154816	MAM	Plaque	Stroke AS	3870	0.84	rs2107595	1.58E-37	0.00024	3.59E-11	rs2107595	rs2520343	rs2107595
TWIST1	7	18157302-20154816	AOR	Plaque	Stroke IS	3874	0.84	rs2107595	1.46E-10	0.00024	2.33E-11	rs2107595	rs2520343	rs2107595
TWIST1	7	18157302-20154816	MAM	Plaque	Stroke IS	3874	0.84	rs2107595	1.58E-37	0.00024	2.33E-11	rs2107595	rs2520343	rs2107595
TWIST1	7	18157302-20154816	AOR	Plaque	Stroke LAS	3890	0.84	rs2107595	1.46E-10	0.00024	1.44E-13	rs2107595	rs2520343	rs2107595
TWIST1	7	18157302-20154816	MAM	Plaque	Stroke LAS	3890	0.84	rs2107595	1.58E-37	0.00024	1.44E-13	rs2107595	rs2520343	rs2107595

Supplementary Table 8. Druggability of genes in loci genome-wide significantly associated with cIMT or plaque. Tier 1, 2 and 3 druggability are highlighed: Tier 1=approved drugs and drugs in clinical development; Tier 2= proteins closely related to drug targets or associated with drug-compounds; Tier 3: extracellular proteins and members of key drug-target families

Gene.name	Drug tier	drug_type	Distance to gene	variant	trait	strength	chr.position	Nearest coding gene	Max number of genes across tissues considered in region	Max number of genes across tissues suggestive assoc
ATG4B	2	SMALL_MOL	-1	rs139302128	cIMT	Suggestive	chr2:242594226	*ATG4B*	29	0
ALPL	2	SMALL_MOL,BIO_MOL	-1	rs147771110	Plaque	Suggestive	chr1:21868723	*ALPL*	23	0
LDLR	2	SMALL_MOL,BIO_MOL	10739	rs200495339	Plaque	Main	chr19:11189298	*LDLR*	70	22
APOB	1	SMALL_MOL,BIO_MOL	19112	rs515135	cIMT	Suggestive	chr2:21286057	*APOB*	18	7
EDNRA	1	SMALL_MOL,BIO_MOL	-1	rs6841473	Plaque	Suggestive	chr4:148407652	*EDNRA*	9	3
APOE	3	BIO_MOL	-1	rs7412	cIMT	Main	chr19: 45412079	*APOE*	61	28

Best coloc	variant	alleles	chr	consequence_types	Rsq EUR
ENSG00000237940 (Blood, PP4=2.26)	rs139302128	C/T	2	non_coding_transcript_variant,non_coding_transcript_exon_variant,NMD_transcript_variant,intron_variant,downstream_gene_variant	0.5
DDOST (SF, PP4=4)	rs147771110	-/C	1	non_coding_transcript_variant,intron_variant,regulatory_region_variant	0.5
ELOF1 (MAM, PP4=5.1)	rs200495339	G/-	19	intergenic_variant	0.5
HS1BP3 (MAM, PP4=5.27)	rs515135	T/C	2	intergenic_variant	0.5
EDNRA (AOR, PP4=37.62)	rs6841473	C/T	4	non_coding_transcript_variant,NMD_transcript_variant,intron_variant	0.5
APOE (SF, PP4=24.84)	rs7412	C/T	19	missense_variant,downstream_gene_variant	0.5

description	biotype	gene	Gene start pos	Gene end pos	Gene window overlap
autophagy related 4B, cysteine peptidase [Source:HGNC Symbol;Acc:20790]	protein_coding	KNOWN	242576628	242613272	36645
alkaline phosphatase, liver/bone/kidney [Source:HGNC Symbol;Acc:438]	protein_coding	KNOWN	21835858	21904905	61570
low density lipoprotein receptor [Source:HGNC Symbol;Acc:6547]	protein_coding	KNOWN	11200038	11244492	10875
apolipoprotein B [Source:HGNC Symbol;Acc:603]	protein_coding	KNOWN	21224301	21266945	3307
endothelin receptor type A [Source:HGNC Symbol;Acc:3179]	protein_coding	KNOWN	148402069	148466106	40938
apolipoprotein E [Source:HGNC Symbol;Acc:613]	protein_coding	KNOWN	45409011	45412650	572

Supplementary Table 9. Druggability of genes identified in colocalization analyses

Gene	Chr	Position	nsnps	data1	PPA.abc	data3	data2	Gene description
CDH13	16	82245780-84245226	7999	AOR	0.94	CARDIoGRAMplusC4D	cIMT	cadherin 13 [Source:HGNC Symbol;Acc:1753]
CDH13	16	82245780-84245226	7999	MAM	0.94	CARDIoGRAMplusC4D	cIMT	cadherin 13 [Source:HGNC Symbol;Acc:1753]
ADAMTS9	3	63588304-65587494	4415	AOR	0.80	AS	cIMT	ADAM metallopeptidase with thrombospondin type 1 motif, 9 [Source:HGNC Symbol;Acc:13202]
ADAMTS9	3	63588304-65587494	4415	MAM	0.77	AS	cIMT	ADAM metallopeptidase with thrombospondin type 1 motif, 9 [Source:HGNC Symbol;Acc:13202]
EDNRA	4	147434590-149433978	2955	AOR	0.80	LAS	PLAQUE	endothelin receptor type A [Source:HGNC Symbol;Acc:3179]

Gene	Drug tier	Drug type	Compound activities	Compound activities
CDH13	tier 3	BIO_MOL	0	0
CDH13	tier 3	BIO_MOL	0	0
ADAMTS9	tier 3	BIO_MOL	0	0
ADAMTS9	tier 3	BIO_MOL	0	0
EDNRA	tier 1	BIO_MOL | SMALL_MOL	46	1*

***Drugs and indications:** AMBRISENTAN (Andes disease,SCD,Asma,HT,HYPERTENSION PULM,Vasc,HPAH,UIP,PULMONARY HYPERTENSION, PRIMARY, DEXFENFLURAMINE-ASSOCIATED,Pulmonary Hypertension, Primary, Fenfluramine-Associated,PAH,Pph1 With Hht,Mountain Sickness,Altitude Hypoxia)|BOSENTAN (Asma,MOD,HYPERTENSION PULM,COLD,melanoma,PSS,Optic Nerve Ischemia,CDH,Morgagni hernia,Bochdalek hernia,HPAH,Anterior Ischemic Optic Neuropathy,Posterior Ischemic Optic Neuropathy,Chronic Airflow Obstruction,UIP,PULMONARY HYPERTENSION, PRIMARY, DEXFENFLURAMINE-ASSOCIATED,Pulmonary Hypertension, Primary, Fenfluramine-Associated,PAH,Pph1 With Hht)|CLAZOSENTAN (Subarachnoid bleeding,Perinatal Subarachnoid Hemorrhage,Spontaneous subarachnoid hemorrhage,Aneurysmal Subarachnoid Hemorrhage,INTRACRANIAL SUBARACHNOID HEMORRHAGE)|DARUSENTAN (HT)|MACITENTAN (HYPERTENSION PULM,PSS,HPAH,UIP,PULMONARY HYPERTENSION, PRIMARY, DEXFENFLURAMINE-ASSOCIATED,Pulmonary Hypertension, Primary, Fenfluramine-Associated,PAH,Pph1 With Hht)|SPARSENTAN (FSGS,HT,Segmental hyalinosis)|TEZOSENTAN (Weak heart,CHF,HYPERTENSION PULM,LVF,rvf,Myocardial Failure,Heart Decompensation)|ZIBOTENTAN (Ca breast,CA,Liver,Tumor,prostate tumor,Liver Dysfunction,BENIGN TUMOR,Ca prostate,Human Mammary Neoplasm,Breast tumor)

Supplementary References

1. Harris, T.B. *et al.* Age, Gene/Environment Susceptibility-Reykjavik Study: multidisciplinary applied phenomics. *Am J Epidemiol* **165**, 1076-87 (2007).
2. The Atherosclerosis Risk in Communities (ARIC) Study: design and objectives. The ARIC investigators. *Am J Epidemiol* **129**, 687-702 (1989).
3. Schmidt, R., Fazekas, F., Kapeller, P., Schmidt, H. & Hartung, H.P. MRI white matter hyperintensities: three-year follow-up of the Austrian Stroke Prevention Study. *Neurology* **53**, 132-9 (1999).
4. Schmidt, R. *et al.* Assessment of cerebrovascular risk profiles in healthy persons: definition of research goals and the Austrian Stroke Prevention Study (ASPS). *Neuroepidemiology* **13**, 308-13 (1994).
5. Ghadery, C. *et al.* R2* mapping for brain iron: associations with cognition in normal aging. *Neurobiol Aging* **36**, 925-32 (2015).
6. Seiler, S. *et al.* Magnetization transfer ratio relates to cognitive impairment in normal elderly. *Front Aging Neurosci* **6**, 263 (2014).
7. Sitzer, M. *et al.* C-reactive protein and carotid intimal medial thickness in a community population. *J Cardiovasc Risk* **9**, 97-103 (2002).
8. Fried, L.P. *et al.* The Cardiovascular Health Study: design and rationale. *Ann Epidemiol* **1**, 263-76 (1991).
9. Bowden, D.W. *et al.* Review of the Diabetes Heart Study (DHS) family of studies: a comprehensively examined sample for genetic and epidemiological studies of type 2 diabetes and its complications. *Rev Diabet Stud* **7**, 188-201 (2010).
10. Aulchenko, Y.S. *et al.* Linkage disequilibrium in young genetically isolated Dutch population. *Eur J Hum Genet* **12**, 527-34 (2004).
11. Dawber, T.R. & Kannel, W.B. The Framingham study. An epidemiological approach to coronary heart disease. *Circulation* **34**, 553-5 (1966).
12. Kannel, W.B., Feinleib, M., McNamara, P.M., Garrison, R.J. & Castelli, W.P. An investigation of coronary heart disease in families. The Framingham offspring study. *Am J Epidemiol* **110**, 281-90 (1979).
13. Splansky, G.L. *et al.* The Third Generation Cohort of the National Heart, Lung, and Blood Institute's Framingham Heart Study: design, recruitment, and initial examination. *Am J Epidemiol* **165**, 1328-35 (2007).
14. Group, C.S. Vascular factors and risk of dementia: design of the Three-City Study and baseline characteristics of the study population. *Neuroepidemiology* **22**, 316-25 (2003).
15. Debette, S. *et al.* Tea consumption is inversely associated with carotid plaques in women. *Arterioscler Thromb Vasc Biol* **28**, 353-9 (2008).
16. Lambert, J.C. *et al.* Meta-analysis of 74,046 individuals identifies 11 new susceptibility loci for Alzheimer's disease. *Nat Genet* **45**, 1452-8 (2013).
17. Deary, I.J., Gow, A.J., Pattie, A. & Starr, J.M. Cohort profile: the Lothian Birth Cohorts of 1921 and 1936. *Int J Epidemiol* **41**, 1576-84 (2012).
18. Deary, I.J. *et al.* The Lothian Birth Cohort 1936: a study to examine influences on cognitive ageing from age 11 to age 70 and beyond. *BMC Geriatr* **7**, 28 (2007).
19. Deary, I.J., Whiteman, M.C., Starr, J.M., Whalley, L.J. & Fox, H.C. The impact of childhood intelligence on later life: following up the Scottish mental surveys of 1932 and 1947. *J Pers Soc Psychol* **86**, 130-47 (2004).
20. Wardlaw, J.M. *et al.* Brain aging, cognition in youth and old age and vascular disease in the Lothian Birth Cohort 1936: rationale, design and methodology of the imaging protocol. *Int J Stroke* **6**, 547-59 (2011).
21. Wardlaw, J.M. *et al.* Vascular risk factors, large-artery atheroma, and brain white matter hyperintensities. *Neurology* **82**, 1331-8 (2014).
22. Bild, D.E. *et al.* Multi-Ethnic Study of Atherosclerosis: objectives and design. *Am J Epidemiol* **156**, 871-81 (2002).
23. de Mutsert, R. *et al.* The Netherlands Epidemiology of Obesity (NEO) study: study design and data collection. *Eur J Epidemiol* **28**, 513-23 (2013).
24. Penninx, B.W. *et al.* The Netherlands Study of Depression and Anxiety (NESDA): rationale, objectives and methods. *Int J Methods Psychiatr Res* **17**, 121-40 (2008).
25. Sullivan, P.F. *et al.* Genome-wide association for major depressive disorder: a possible role for the presynaptic protein piccolo. *Mol Psychiatry* **14**, 359-75 (2009).
26. McQuillan, R. *et al.* Runs of homozygosity in European populations. *Am J Hum Genet* **83**, 359-72 (2008).

27. Hofman, A. *et al.* The Rotterdam Study: 2010 objectives and design update. *Eur J Epidemiol* **24**, 553-72 (2009).
28. Volzke, H. *et al.* Cohort profile: the study of health in Pomerania. *Int J Epidemiol* **40**, 294-307 (2011).
29. Raitakari, O.T. *et al.* Cohort profile: the cardiovascular risk in Young Finns Study. *Int J Epidemiol* **37**, 1220-6 (2008).
30. Lawlor, D.A., Bedford, C., Taylor, M. & Ebrahim, S. Geographical variation in cardiovascular disease, risk factors, and their control in older women: British Women's Heart and Health Study. *J Epidemiol Community Health* **57**, 134-40 (2003).
31. Price, J.F. *et al.* The Edinburgh Type 2 Diabetes Study: study protocol. *BMC Endocr Disord* **8**, 18 (2008).
32. Wadsworth, M., Kuh, D., Richards, M. & Hardy, R. Cohort Profile: The 1946 National Birth Cohort (MRC National Survey of Health and Development). *Int J Epidemiol* **35**, 49-54 (2006).
33. Marmot, M.G. *et al.* Health inequalities among British civil servants: the Whitehall II study. *Lancet* **337**, 1387-93 (1991).
34. Baldassarre, D. *et al.* Cross-sectional analysis of baseline data to identify the major determinants of carotid intima-media thickness in a European population: the IMPROVE study. *Eur Heart J* **31**, 614-22 (2010).
35. Loeffler, M. *et al.* The LIFE-Adult-Study: objectives and design of a population-based cohort study with 10,000 deeply phenotyped adults in Germany. *BMC Public Health* **15**, 691 (2015).
36. Beutner, F. *et al.* Rationale and design of the Leipzig (LIFE) Heart Study: phenotyping and cardiovascular characteristics of patients with coronary artery disease. *PLoS One* **6**, e29070 (2011).
37. Boyd, A. *et al.* Cohort Profile: the 'children of the 90s'--the index offspring of the Avon Longitudinal Study of Parents and Children. *Int J Epidemiol* **42**, 111-27 (2013).
38. Fraser, A. *et al.* Cohort Profile: the Avon Longitudinal Study of Parents and Children: ALSPAC mothers cohort. *Int J Epidemiol* **42**, 97-110 (2013).
39. Galesloot, T.E. *et al.* Cohort Profile: The Nijmegen Biomedical Study (NBS). *Int J Epidemiol* (2017).
40. Berglund, G., Elmstahl, S., Janzon, L. & Larsson, S.A. The Malmo Diet and Cancer Study. Design and feasibility. *J Intern Med* **233**, 45-51 (1993).
