# Supplementary material for: GWAS and colocalization analyses implicate carotid intima-media thickness and carotid plaque loci in cardiovascular outcomes
Source: Nat Commun. 2018 Dec 3;9:5141. doi: 10.1038/s41467-018-07340-5 (PMC6277418; doi:10.1038/s41467-018-07340-5)
Supplement: Supplementary file 5 — Description of Additional Supplementary Files [file 41467_2018_7340_MOESM5_ESM.pdf]

Description of Additional Supplementary Files

Supplementary Data 1. Colocalization results between tissue-specific eQTLs of five genes (CCDC71L, PRKAR2B, ADAMTS9, LOXL4) with CHD and stroke (coloc)

Supplementary Data 2. GO Slim analysis for protein-coding genes identified in GWAS meta-analyses (Tables 1 and S5) of cIMT (A); plaque (B) and genes identified in co-localization of cIMT/plaque
