# Supplementary material for: GWAS and colocalization analyses implicate carotid intima-media thickness and carotid plaque loci in cardiovascular outcomes
Source: Nat Commun. 2018 Dec 3;9:5141. doi: 10.1038/s41467-018-07340-5 (PMC6277418; doi:10.1038/s41467-018-07340-5)
Supplement: Supplementary file 2 — Supplementary Data 1 [file 41467_2018_7340_MOESM2_ESM.pdf]

Reviewers' Comments:

Reviewer #1:
Remarks to the Author:
Franceschini and colleagues report a meta-analysis of GWAS of carotid artery intima media thickness and carotid plaque measures of subclinical atherosclerosis associated with ischemic stroke and CHD. A number of novel loci were identified and these were integrated with eQTL data to help prioritize candidate genes. The authors also explored the possible involvement of the loci in stroke and CAD. In general, the methods appear to be appropriate and the conclusions justified, but I have some minor comments.

1. The "moloc" algorithm was used to test shared influences of cIMT on CHD/stroke, but the reference (#14) appears to be missing. A URL is listed in the end of the Discussion.

2. Figure 1, containing the key association data, is very hard to read. Perhaps it could be broken up into multiple tables or a portion of the data included in a Supplemental Table. The columns for GTEx and STARNET need to be better defined.

3. The paper is terse and a number of statistical terms such as "posterior probabilities" will be confusing to non-statistician readers. In the abstract "LD scores analyses" should be defined.

4. Perhaps I misread it, but did the authors find support for the previously identified ZHX2, APOC1, and PINX1 loci?

Reviewer #2:
Remarks to the Author:
The authors report an extended GWAS of cIMT and plaque, two important cardiovascular surrogate outcomes. A number of novel loci are identified and eQTL/colocalisation analyses are used to identify potential causal candidate genes. The manuscript provides interesting new findings and sheds light on the overlap (or not) of plaque/cIMT loci with cardiovascular outcomes, and will therefore be of interest to the cardiovascular genetics community. I have a few concerns about the findings and interpretation though, which I list below:

General comments:

1) Inference from colocalisation analyses:
in some of the interpretation of the results deriving from the colocalisation analyses, the authors are appropriately cautious (eg, lines 498-500, where CCDC71L and PRKAR2B are described as the "most likely causal genes"). However, in other places, the interpretation implies that the effects of the PIK3CG locus on cardiovascular outcomes are definitively mediated through both of these genes (eg, suggesting that cIMT/plaque would be good surrogate endpoints in trials that intervene on either of these proteins). I don't believe the data presented are sufficient to assume that the proteins encoded by the genes with colocalising expression are necessarily aetiologically relevant to disease outcomes.

Take for example a causal variant that is associated with expression of 5 genes and is also the causal variant for a disease endpoint. It seems unlikely to me that the alterations in expression of all 5 genes are each involved in causing the disease. There may of course also be other phenotypes associated with the variant (eg, expression of genes that remain as yet undetected or pathways that do not necessarily lie through changes in gene expression) that could be driving the disease process. Without additional evidence to show which, if any, of the genes are causally involved in disease, the best we can realistically do with colocalisation is to identify promising candidate causal genes for further testing. The language in the manuscript should be revised to

make this clear.

2) Low frequency signals:
it seems surprising, to me at least, that 3 of the 4 novel plaque loci are low frequency variants. Given that most GWAS initially identified common variants and then were able to identify lower frequency variants only when sample sizes increased, are the authors surprised by this? To increase reader confidence in these signals, it would be important to include forest plots for these variants to establish that the signal is not being driven by one or two aberrant studies. It may also be helpful to provide cluster plots for the variant (or strongest genotyped proxy variant if the variant was imputed).

3) Definition of plaque:
there seem to be very different plaque definitions used across studies according to STable 2 – could the authors comment on the effect this had (or may have had) on their findings and ability to discover associations?

4) Methods description:
there are some oversimplifications in the Methods that give the impression this was a simple straightforward GWAS imputed to 1000 Genomes. However, the Supplementary Tables show that a mix of genotyping array types was used (eg, some genomewide, some actually Metabochip so far from genomewide) and a mix of imputation reference panels.

5) Data availability:
can the authors confirm that they will be making the GWAS summary statistics publicly available to benefit the community (in the same way as they have benefited from the public availability of GWAS summary statistics from the CARDIoGRAMplusC4C Consortium for example)?

Specific comments on text, tables and figures:

270: 1000 Genomes, “densely imputed”? In the days of HRC, TopMed etc, I’m not sure one would consider 1000 Genomes to be particularly dense imputation anymore.

271: it wasn’t immediately clear what the “cases” were of

333: not clear what sigma represents

340-346: much of this information is repeated – suggest just reporting the rsIDs and the nearest genes in one sentence (same for lines 358-361)

347: “loci” should be “locus”

348-349: describing “the chromosome 8 locus” is confusing. Why not say “the two signals on chromosome 8 near MCPH1 ((rs2912063) and SGK223 (rs11785239) were confirmed to be independent through conditional analysis”? On a related note, it would be helpful to define in the results what you consider a locus.

349-351: if you identified a novel independent locus near PINX1, shouldn’t this appear under “novel loci for cIMT” in Table 1, with the association results for the previously published signal appearing under “known loci for cIMT”?

353: Text here implies “suggestive significance” was defined as p<1x10^-6, but STable 5 uses p<1x10^-7, as do other lines in the text.

355-357: I didn’t find this evidence particularly compelling. How likely is it that 9/13 would be

concordant under the null? What is the power to detect these associations in the African dataset at a Bonferroni-adjusted p-value threshold? If this experiment has now power to say anything conclusive about the association of genetic variants with cIMT/plaque, I would suggest removing it until a more powerful African ancestry dataset can be brought together.

364-366: this sentence is confusing, suggest rewriting.
Results/Figure 1: how many variants were actually included in the meta-analysis after QC?

377-384: I found this whole section difficult to understand. If it is only variants in the PINX1 region that have a high probability of being causal for cIMT, what does that mean for variants in your other loci? That there is no causal variant? Or that you haven't imputed the causal variant? SFigure 3 needs a much clearer legend to help the reader – how does the LocusZoom plot at the top differ from the one for this region in SFigure 2? Why do there seem to be horizontal bands of variants at the top of this LocusZoom? Why does the text state that the PP was >0.9 but the plot suggests very low PPs and the legend suggests the most likely causal variant only has a PP of 1%?

390: what does this sentence mean? How did your analyses "control" gene expression?

393: the direction of the eQTL signal across tissues isn't clear from Table 1 – either I've missed it or it isn't there, so it's hard to assess the "consistency of direction"

407: Methods section suggests that PP>0.5 was considered strong evidence so why PP>75% here?

415-416: I didn't understand this sentence.

475-477: why doesn't ADAMTS9 appear in S Table 10? Because of the filtering done?

496-497: I don't buy the argument that because you found co-localising eQTLs in relevant tissues for 3 suggestively associated variants that these can then be considered "novel loci associated with cIMT or carotid plaque". I would describe them throughout the manuscript as "potential additional loci"

524-526: how does the stronger correlation of plaque with CAD/stroke compared to cIMT highlight "the clinical relevance of our findings"?

575: "generalization"

629: would be helpful to spell out acronyms

632: how was "suggestive" significance defined?

676-678: I didn't understand this – how can a gene be in LD with a variant?

753: ref #14 needs a proper reference

Table 1: the eQTL data is very difficult to read due to the poor layout – could this be tidied up somehow?

Table 1: Please report the actual insertion/deletion alleles rather than 'I/D'

Table 1: why use p<0.01? How does this relate to 5% FDR?

Table 1: for plaque, it would be more intuitive to report an odds ratio and confidence interval

Table 2: I don't think a colocalisation analysis can really confirm that the effect of a variant on a trait is mediated by its effect on gene expression. Although it confirms that the trait signal and eQTL signal are the same, there may be multiple gene expression signals that colocalise with a trait association, and that doesn't mean that changes in the expression of all the genes are aetiologically relevant. Similarly, on lines 417-418, I don't think you can definitively say from colocalisation that the loci are influencing cIMT/plaque through expression.

Table 3: are these p-values already adjusted for multiple testing?

Figure 3: not really clear why this was picked as a main Figure? Wouldn't it make more sense to include both CCDC71L and PRKAR2B given the point is that both these eQTLs colocalise with the cIMT & plaque signals? In fact, the most interesting thing about the colocalisation is probably the contrast of signals for the same gene that do and don't overlap (eg, stroke vs CHD; MAM/AOR vs SF) – wouldn't it be informative to show the contrasting regional associations for these traits, rather than just the ones that do co-localise, which inevitably ends up with very similar-looking polots?

STable 5: the betas and standard errors look strange for the cIMT variants – did they really have exactly the same beta (albeit opposite direction)? If so, how do they have similar p-values but one has a 10-fold smaller standard error?

SFigure 1: can you make it clearer on the Manhattan plots which locus name points to which signal. This would help clear up confusion. Can you also explain why there appear to be some genomewide signals which are not listed in Table 1, eg, there are clearly 2 distinct signals on chromosome 1, but there is only 1 novel or known cIMT locus in Table 1.

SFigure 2: please make sure all plots have LD colouring.

SFigure 2: for some plots, the labelled variant is not the lead – how was this variant chosen?

We are grateful to the Reviewers for their detailed review and helpful comments. Below, please find a point by point response to each of the Reviewers' comments:

Reviewer #1 (Remarks to the Author):

Franceschini and colleagues report a meta-analysis of GWAS of carotid artery intima media thickness and carotid plaque measures of subclinical atherosclerosis associated with ischemic stroke and CHD. A number of novel loci were identified and these were integrated with eQTL data to help prioritize candidate genes. The authors also explored the possible involvement of the loci in stroke and CAD. In general, the methods appear to be appropriate and the conclusions justified, but I have some minor comments.

1. The “moloc” algorithm was used to test shared influences of cIMT on CHD/stroke, but the reference (#14) appears to be missing. A URL is listed in the end of the Discussion.

Co-Author Response: Thank you for this. The paper describing *moloc* is now published in Bioinformatics (PMID=29579179). We have updated reference #14. We have included a genome-wide analyses using this method, which is a multi-trait extension of our previously developed two-trait model described in *coloc* (reference #10, Giambartolomei *et al.*, 2014). This analysis provides further support for involvement in clinically apparent CHD/stroke for two out of three regions found in 2-trait co-localization (*CCDC71L & PRKAR2B, ADAMTS9*), and additional potential genes that share the same causal variant with CHD or stroke.

2. Figure 1, containing the key association data, is very hard to read. Perhaps it could be broken up into multiple tables or a portion of the data included in a Supplemental Table. The columns for GTEx and STARNET need to be better defined.

Co-Author Response: We have revised Figure 1, which now includes two different panels illustrating the initial GWAS analysis (Panel A) and functional follow-up using co-localization and LD score regression (Panel B). We believe the new Figure 1 now defines better our study design and approaches. GWAS results (Panel A) are listed in Table 1 of the main text. Two-trait co-localization in Table 2, LD score regression in Table 3, and 3-trait co-localization using *moloc* in Supplementary Table 8.
Please note that some of the results have changed for plaque meta-analyses, LD-score and *moloc* analyses due to revision of plaque meta-analyses, updated analyses including stroke GWAS data from the MEGASTROKE consortia (updated from METASTROKE), and addition of new results based on a 3-way *moloc* new statistical method.

3. The paper is terse and a number of statistical terms such as “posterior probabilities” will be confusing to non-statistician readers. In the abstract “LD scores analyses” should be defined.

Co-Author Response: Thank you for the suggestion for greater readability. We have revised the manuscript to clarify the statistical methods using posterior probabilities, and we minimize references to “posterior probabilities”, when possible. We changed the abbreviation LD score to Linkage disequilibrium score and have added a brief clarification. Further details on LD score regression is defined in the Online method section; LD score regression was used to compute the genetic correlation between cIMT/plaque with CHD and stroke subtypes.

4. Perhaps I misread it, but did the authors find support for the previously identified ZHX2, APOC1, and PINX1 loci?

Co-Author Response: Yes, Table 1 shows the associations at these loci. Note that the *APOC1* locus is listed as *APOE* since it has been fine-mapped to this gene in more recent publications. This information is included in the introduction. Using functional data, we find additional support for *PINX1* being the most likely gene in the region (Supplementary Figure 4).

Reviewer #2 (Remarks to the Author):

The authors report an extended GWAS of cIMT and plaque, two important cardiovascular surrogate outcomes. A number of novel loci are identified and eQTL/co-localisation analyses are used to identify potential causal candidate genes. The manuscript provides interesting new findings and sheds light on the overlap (or not) of plaque/cIMT loci with cardiovascular outcomes, and will therefore be of interest to the cardiovascular genetics community.

Co-Author Response: Thank you.

I have a few concerns about the findings and interpretation though, which I list below:
General comments:

1) Inference from co-localisation analyses:
in some of the interpretation of the results deriving from the co-localisation analyses, the authors are appropriately cautious (eg, lines 498-500, where CCDC71L and PRKAR2B are described as the "most likely causal genes"). However, in other places, the interpretation implies that the effects of the PIK3CG locus on cardiovascular outcomes are definitively mediated through both of these genes (eg, suggesting that cIMT/plaque would be good surrogate endpoints in trials that intervene on either of these proteins). I don't believe the data presented are sufficient to assume that the proteins encoded by the genes with co-localising expression are necessarily aetiologically relevant to disease outcomes.

Co-Author Response: Thank you for the comment. We completely agree that the genes prioritized in co-localization analysis need further exploration and certainly cannot be defined as causal from our analyses alone, and thank the Reviewer for pointing this out. We have revised the paragraph related to surrogate end-points in trials, see also answer to question below.

Take for example a causal variant that is associated with expression of 5 genes and is also the causal variant for a disease endpoint. It seems unlikely to me that the alterations in expression of all 5 genes are each involved in causing the disease. There may of course also be other phenotypes associated with the variant (eg, expression of genes that remain as yet undetected or pathways that do not necessarily lie through changes in gene expression) that could be driving the disease process. Without additional evidence to show which, if any, of the genes are causally involved in disease, the best we can realistically do with co-localisation is to identify promising candidate causal genes for further testing. The language in the manuscript should be revised to make this clear.
Co-Author Response: We agree with the Reviewer that the evidence from our analyses that the same variant is associated with expression of both a specific gene and a disease does not necessarily imply causality. Our analytic method provides a statistical method that harnesses

prior knowledge to prioritize specific genes for further analysis. In response to these concerns, we have revised some sections as shown below.
Page 15, 2nd paragraph: "The co-localization analyses provides additional insights in the relationships between subclinical atherosclerosis, clinical outcomes and tissue-specific regulation at specific genomic regions. For example, our suggestive top gene association in multi-trait co-localization for *KIAA1462* included MAM eQTLs, carotid plaque and CHD, supporting the shared genetic effects at this locus in atherosclerosis of carotid and coronary arteries. *KIAA1462* has been previously reported in the same locus identified by GWAS for CHD.[21]".
Page 15, 3rd paragraph: "Additional studies in diverse and large samples across the multiple datasets are needed to explore these results further. As more summary statistics become available for other clinical end-points beyond stroke and CHD (both in terms of larger-sample size and richer genome coverage), and as further refinements in clinical phenotypes emerge (e.g. from CHD to acute coronary syndrome sub-components), strategies to integrate this knowledge using methods such as *moloc*[10] and *eCAVIAR*[24] will continue to be essential for harnessing genome-wide findings in the drug-discovery process."

2) Low frequency signals:
it seems surprising, to me at least, that 3 of the 4 novel plaque loci are low frequency variants. Given that most GWAS initially identified common variants and then were able to identify lower frequency variants only when sample sizes increased, are the authors surprised by this? To increase reader confidence in these signals, it would be important to include forest plots for these variants to establish that the signal is not being driven by one or two aberrant studies. It may also be helpful to provide cluster plots for the variant (or strongest genotyped proxy variant if the variant was imputed).

Co-Author Response: We thank the Reviewer for the very helpful suggestion. In response to the Reviewer's query, we have thoroughly reviewed the analyses and we identified one participating study with inconsistent results for plaque. Upon revision of the analysis for plaque in this study, the new meta-analysis results no longer support the associations with these low frequency variants. We have accordingly revised all results related to carotid plaque analyses (Tables 1 and 2, Supplementary Tables 5 to 11, Figure 1 and Supplementary Figures 1 to 8). We are reassured that the revised results for plaque includes one novel locus (*LDLR*) and replication of four known loci. We now also include Forest plots for all variants identified for cIMT and for plaque (both novel and replication) in a new Supplementary Figure 3. This revision also strengthens our downstream analysis, providing stronger, more coherent correlations locally (co-localization) and genome-wide (LD score regression).

3) Definition of plaque:
there seem to be very different plaque definitions used across studies according to STable 2 – could the authors comment on the effect this had (or may have had) on their findings and ability to discover associations?

Co-Author Response: We acknowledge that there are differences in the definition of plaque, as the measures varied across different studies. Most studies have recorded plaque during ultrasound analyses but for the few that did not have this measure, we used a definition based on carotid stenosis of 25% or greater (e.g., Framingham Heart Study). This heterogeneity in definitions likely reduced the power to identify novel variants, and our study is limited for the identification of plaque subtypes. However, we have used the same

definitions used in prior publications (e.g., , reference #4, Bis J *et al,* 2011) providing the best available efforts to harmonize this clinically relevant trait.

4) Methods description:
there are some oversimplifications in the Methods that give the impression this was a simple straightforward GWAS imputed to 1000 Genomes. However, the Supplementary Tables show that a mix of genotyping array types was used (eg, some genomewide, some actually Metabochip so far from genomewide) and a mix of imputation reference panels.

Co-Author Response: Given the limited space to describe this in the Online Methods, we have included details on GWAS imputation and quality control in Supplementary Table 3. We used standardized protocols in the CHARGE and UCLEB consortium for imputation using a Phase 1 integrated (March 2012 release) reference panel from the 1000G build h19, pre- and post-imputation quality control
(script developed by Winkler et al – ref 28), and filtered out SNPs that had a minor allele frequency of 1% or less. These approaches have been previously implemented in several other publications and are now routinely applied to GWAS meta-analyses. We also allowed studies with genome-wide association arrays and metabolochip in our study to increase power to identify SNP associations. In summary, all studies were imputed to 1000G reference panel for European ancestry or cosmopolitan panels, and we used the same hg19 reference annotation. Supplementary Table 3 description of the approaches has been revised for clarity.

5) Data availability:
can the authors confirm that they will be making the GWAS summary statistics publicly available to benefit the community (in the same way as they have benefited from the public availability of GWAS summary statistics from the CARDIoGRAMplusC4C Consortium for example)?

Co-Author Response: Yes, we plan to share summary data as per policy of the journal. We will upload the results into dbGaP as described in Rich SS *et al*, Nature Genetics 2016;48:702-3.

Specific comments on text, tables and figures:

270: 1000 Genomes, “densely imputed”? In the days of HRC, TopMed etc, I’m not sure one would consider 1000 Genomes to be particularly dense imputation anymore.

Co-Author Response: Thank you, we have revised the text to “1000 Genomes imputed data”.

271: it wasn’t immediately clear what the “cases” were of

Co-Author Response: Thank you, we revised the text for clarity. Of note, among 48,434 individuals, 21,540 had a carotid plaque based on our definition.

333: not clear what sigma represents

Co-Author Response: We have changed the term “sigma” to “heterogeneity”.

340-346: much of this information is repeated – suggest just reporting the rsIDs and the nearest genes in one sentence (same for lines 358-361)

Co-Author Response: We had extensive discussions on how to present the novel loci, either by reporting the leading SNP or the nearby gene at each locus. We decided to report the leading SNP and nearby gene to help address the results from our co-localization analyses, which points to the most likely functionally implicated gene based on eQTLs. Although this information is somewhat redundant, it helps to understand findings for some loci where the nearby gene was not the mostly likely functional gene identified in co-localization analyses. For example, SNP rs13225723 was previously associated with the *PIK3CG* gene, while co-localization analysis points to *CCDC71L* and *PRKAR2B* genes as the most likely candidate genes responsible for the GWAS signal.

347: "loci" should be "locus"

Co-Author Response: Thank you, we have made this revision.

348-349: describing "the chromosome 8 locus" is confusing. Why not say "the two signals on chromosome 8 near MCPH1 ((rs2912063) and SGK223 (rs11785239) were confirmed to be independent through conditional analysis"? On a related note, it would be helpful to define in the results what you consider a locus.

Co-Author Response: Thank you, we have clarified what we mean by "a locus". We defined a locus as the region within 1 MB of the most significant SNP at each region. This information is available on Online methods, page 17, under the section "Conditional analysis".

349-351: if you identified a novel independent locus near PINX1, shouldn't this appear under "novel loci for cIMT" in Table 1, with the association results for the previously published signal appearing under "known loci for cIMT"?

Co-Author Response: We identified a new independent SNP association at the known *PINX1* region, as defined above. Therefore, this SNP is not listed in Table 1. However, we agree with the Reviewer that this information is important and we now include it in the abstract:
"We identified eight novel susceptibility loci for cIMT, **a new independent association at the previously-identified *PINX1* locus** (chr8:10606223-indel)**,** and one novel locus for carotid plaque at P value < 5.0×10^{-8}. "

353: Text here implies "suggestive significance" was defined as p<1x10^-6, but STable 5 uses p<1x10^-7, as do other lines in the text.

Co-Author Response: Thank you for pointing this out. We corrected the text to indicate that the definition of suggestive association is p<1×10^{-7}.

355-357: I didn't find this evidence particularly compelling. How likely is it that 9/13 would be concordant under the null? What is the power to detect these associations in the African dataset at a Bonferroni-adjusted p-value threshold? If this experiment has now power to say anything conclusive about the association of genetic variants with cIMT/plaque, I would suggest removing it until a more powerful African ancestry dataset can be brought together.

Co-Author Response: We agree with the Reviewer that these results are not informative and we have removed them from the manuscript.

364-366: this sentence is confusing, suggest rewriting.

Co-Author Response: We have re-written this sentence as:
"At four known loci associated with carotid plaque (nearby genes *EDNRA*, *PIK3CG*, *CFDP1-TMEM170A*, and at the 9p21 region), the most significantly associated variants were in LD with the previously reported SNPs (**Table 1**), [4,6,7] indicating that these SNPs mark the same association at each locus."

Results/Figure 1: how many variants were actually included in the meta-analysis after QC?

Co-Author Response: 9,574,088 SNPs for the cIMT meta-analysis and 8,578,107 SNPs for the plaque meta-analysis. We now include this information to the Online methods, page 17, second paragraph.

377-384: I found this whole section difficult to understand. If it is only variants in the PINX1 region that have a high probability of being causal for cIMT, what does that mean for variants in your other loci? That there is no causal variant? Or that you haven't imputed the causal variant?

Co-Author Response: We apologize for the lack of clarity. The probability of association depends solely on the association statistics of the GWAS, and multiple regions have a high probability to harbor causal variants. The goal of the fGWAS analysis was to assess the regions that also have a high probability to have a causal SNP based on the presence of functional annotations.
We have modified the text to clarify this point:
"To better define potentially causal variants within the identified genetic risk loci, we jointly analyzed the GWAS data with functional genomic information such as annotations on active transcription sites or open chromatin regions (i.e., performed a fine-mapping functional genome wide association analysis using fGWAS[13]). Only variants in the *PINX1* region were found to have a high probability that its association with cIMT is driven by SNPs that fall within transcription sites in adipose derived mesenchymal stem cells at a DNaseI-hypersensitive site (Supplementary Figure **4**), a finding that for the first time provides a down-stream mechanistic explanation for the cIMT signal in the *PINX1* locus. "

SFigure 3 needs a much clearer legend to help the reader – how does the LocusZoom plot at the top differ from the one for this region in SFigure 2? Why do there seem to be horizontal bands of variants at the top of this LocusZoom? Why does the text state that the PP was >0.9 but the plot suggests very low PPs and the legend suggests the most likely causal variant only has a PP of 1%?

Co-Author Response: We revised the figure and change "Posterior Probability" since this is actually a "Prior Probability" as the Reviewer noted.
We have also revised the label for Supplementary Figure 4 (note it was changed from S3 to S4 in the revision) to*:*
"Regional plot surrounding the *PINX1* locus for cIMT. The top panel shows the P values for SNPs association with cIMT. The middle panel shows the overlaps of SNPs with annotations

included in the combined model in fGWAS. The bottom panel shows the fitted empirical prior probability based on the fGWAS combined model. The SNP association shown in purple (chr8:10659406; P = 6.4 x 10^{-11}) falls within active transcription (REMC.coreHMM.FAT_ADIP_DR_MSC.5_TxWk) in Adipose Derived Mesenchymal Stem Cells and a DNaseI-hypersensitive site (ENCODE.DHS_Maurano.CMK.DS12393) leading the model to assign a higher probability compared to the index SNP (index SNP chr8:10606223:INDEL; P = 1.3 x 10^{-12})."

390: what does this sentence mean? How did your analyses "control" gene expression?

Co-Author Response: We agree this is confusing and we have removed this sentence.

393: the direction of the eQTL signal across tissues isn't clear from Table 1 – either I've missed it or it isn't there, so it's hard to assess the "consistency of direction"

Co-Author Response: Thank you, we agree this table does not show the direction of effect, so we have removed this sentence.

407: Methods section suggests that PP>0.5 was considered strong evidence so why PP>75% here?

Co-Author Response: Thanks you for noticing. The correct PP is ≥75%. We have revised the methods section accordingly.

415-416: I didn't understand this sentence.

Co-Author Response: We have substantially revised the sentence:
The sentence now reads: "We found a low probability of co-localization of the SNP with the *PIK3CG* gene expression (<1%)."

475-477: why doesn't ADAMTS9 appear in S Table 10? Because of the filtering done?

Co-Author Response: Table S10 only included genes nearby our GWAS significant findings, and the most significant associated SNP at *ADAMTS9* has a p-value with cIMT/plaque of 1.5 x 10^{-6}. Table S11 includes genes from our co-localization signals which are not filtered by any threshold, so for some of the genes, the top SNP do not reach the GWAS p-value threshold for significance. We revised the title of Table S10 to: "Druggability of genes in loci genome-wide significantly associated with cIMT or plaque". Table S10 title now reads: "Druggability of genes identified in co-localization analyses". We added a column with the GWAS p-values and a footnote to Table S11: "Note some of the genes identified in co-localization analyses, the SNP does not reach the genome-wide association threshold."

496-497: I don't buy the argument that because you found co-localising eQTLs in relevant tissues for 3 suggestively associated variants that these can then be considered "novel loci associated with cIMT or carotid plaque". I would describe them throughout the manuscript as "potential additional loci"
Co-Author Response: We agree with the Reviewer and have adjusted our statements in several places throughout the text as outlined below. Additionally, we have re-focused the

Discussion to reflect mostly our GWAS results, with co-localization results providing supplementary information regarding potentially new signals.

Abstract: "at two potentially additional loci"
Paragraph title (page 11, 2nd paragraph): "Co-localization analysis of meta-analysis of GWAS data and STARNET eQTLs identifies potential additional cIMT/plaque genes."
"The eQTL associations at two additional loci (*ADAMTS9*, *LOXL4*) in MAM or AOR showed evidence of co-localization with cIMT or plaque, although GWAS association p-values at these loci did not meet the genome-wide significance threshold (Table 2, Supplementary Figure 5). Albeit with weaker magnitudes, the expression of these two genes were also associated with the top co-localizing SNPs as detected in RNAseq data in GTEx aorta (rs17676309, chr3:64730121, *ADAMTS9*, p= 0.0003 and rs55917128, chr10:100023359, *LOXL4*, p = 0.0005)."
Discussion (page 15, 2nd paragraph): "The integration of GWAS and tissue-specific *cis*-eQTLs for the joint analyses of tissue-specific eQTLs from CHD patients identified two potentially additional loci co-localizing with cIMT or carotid plaque: chr3:63561280-65833136 (*ADAMTS9*), chr10:99017729-101017321 (*LOXL4).*"

524-526: how does the stronger correlation of plaque with CAD/stroke compared to cIMT highlight "the clinical relevance of our findings"?

Co-Author Response: Thank you, we have removed this sentence.

575: "generalization"

Co-Author Response: We used this term to highlight our attempts to replicate an association in a different ancestral population than the European descent population in which the variant association was identified. If variants identified in our study also have low p-values in African Americans, the association in African Americans may be considered to generalize to associations identified in individuals of European ancestry. However, as noted above, given the small sample sizes of African Americans with carotid information, we acknowledge that statistical power is limited to confidently these associations. Please note that we have removed the results related to African ancestry so the clarification in no longer needed.

629: would be helpful to spell out acronyms

Co-Author Response: Thanks, we have revised the text.

632: how was "suggestive" significance defined?

Co-Author Response: We defined "suggestive" by a p-value < $1x\ 10^{-7}$, see response above to Reviewer 1.

676-678: I didn't understand this – how can a gene be in LD with a variant?

Co-Author Response: Thank you for this note. The LD is between variants close-by an associated GWAS SNP and variants overlapping genes. We clarified how the LD block was constructed around each GWAS cIMT/plaque variant in Methods (page 20, 2nd paragraph):

"The LD block around top SNPs associated with cIMT and carotid plaque was constructed using LD information from the 1000 Genomes panel, as previously outlined in Finan et al.[16] Briefly, the boundaries of the LD region were defined as the positions of the variants furthest upstream and downstream of a GWAS SNP with an r^2 value of ≥0.5 and within a 1-Mbp flank on either side of the GWAS variant. Associated variants that were not present in the 1000 Genomes panel that were not in LD with any other variants were given a nominal flank of 2.5 kbp on either side of the association. Gene annotations using Ensembl version 79 were then overlapped to the LD region."

753: ref #14 needs a proper reference

Co-Author Response: We have updated this reference.

Table 1: the eQTL data is very difficult to read due to the poor layout – could this be tidied up somehow?

Co-Author Response: Thank you, we have simplified the reporting of eQTLs to make it clearer.

Table 1: Please report the actual insertion/deletion alleles rather than 'I/D'

Co-Author Response: As per your suggestion, this is now reported.

Table 1: why use p<0.01? How does this relate to 5% FDR?

Co-Author Response: In Table 1, we intentionally used a nominal cut-off of p<0.01 to be most inclusive of possible eQTLs that have a shared signals with the GWAS. In our co-localization analyses, we formally test this. If the Reviewer thinks a different cut-off would be most appropriate here we would be happy to modify this accordingly.

Table 1: for plaque, it would be more intuitive to report an odds ratio and confidence interval

Co-Author Response: Thank you for the suggestion. Odds Ratio and 95% CI for plaque meta-analyses are now shown in the forest plots included in Supplementary Figure 3.

Table 2: I don't think a co-localisation analysis can really confirm that the effect of a variant on a trait is mediated by its effect on gene expression. Although it confirms that the trait signal and eQTL signal are the same, there may be multiple gene expression signals that co-localise with a trait association, and that doesn't mean that changes in the expression of all the genes are aetiologically relevant. Similarly, on lines 417-418, I don't think you can definitively say from co-localisation that the loci are influencing cIMT/plaque through expression.

Co-Author Response: We agree with the Reviewer, our intentions were not to claim proof of causality between altered gene expression and clinical trait, but rather to provide evidence of shared control from the same genetic variant. We have modified the text to reflect this clarification.

Table 3: are these p-values already adjusted for multiple testing?

Co-Author Response: The LD score unadjusted p-values are reported in Table 3. After adjusting for 10 tests (2 subclinical traits and 5 outcomes), the threshold for significance is 0.005. Based upon this threshold, the genetic correlations between cIMT/plaque and CHD, and cIMT and any stroke and ischemic stroke are significant. We revised the manuscript sections including abstract, results, discussion and Online Methods to include the information on significance based on this threshold.

Figure 3: not really clear why this was picked as a main Figure? Wouldn't it make more sense to include both CCDC71L and PRKAR2B given the point is that both these eQTLs co-localise with the cIMT & plaque signals? In fact, the most interesting thing about the co-localisation is probably the contrast of signals for the same gene that do and don't overlap (eg, stroke vs CHD; MAM/AOR vs SF) – wouldn't it be informative to show the contrasting regional associations for these traits, rather than just the ones that do co-localise, which inevitably ends up with very similar-looking polots?

Co-Author Response: We thank the Reviewer for this suggestion and have revised Figure 3 as suggested. In particular, we now show the results from our CIMT and plaque associations at the *CCDC71L* locus (Panel A and B, Figure 3), and eQTL at this locus in AOR/MAM, which, as the Reviewer pointed out, are the same for the AOR and MAM tissue(Panel C, Figure 3), and eQTLs in SF at this locus (Panel D, Figure 3). We agree that this new figure provides better visualization of our results for the cIMT/plaque association signal overlapping only with the AOR/MAM eQTLs and not with the SF.

STable 5: the betas and standard errors look strange for the cIMT variants – did they really have exactly the same beta (albeit opposite direction)? If so, how do they have similar p-values but one has a 10-fold smaller standard error?

Co-Author Response: The betas and SE are correct. There is some variation in the sample sizes for the SNPs reported in Table S5 ($P<10^{-7}$) that can explain the lower precision (larger standard errors) of some of the estimates. P-values are also correct and can be verified from betas/standard errors using the Wald test.

SFigure 1: can you make it clearer on the Manhattan plots which locus name points to which signal. This would help clear up confusion. Can you also explain why there appear to be some genomewide signals which are not listed in Table 1, eg, there are clearly 2 distinct signals on chromosome 1, but there is only 1 novel or known cIMT locus in Table 1.

Co-Author Response: As per the Reviewer recommendation, we have updated the Manhattan plots and highlighted the new loci.

SFigure 2: please make sure all plots have LD colouring.

Co-Author Response: Because the LD for some SNPs are not available in the LocusZoom data and we are unable to show those SNP in color.

SFigure 2: for some plots, the labelled variant is not the lead – how was this variant chosen?

Co-Author Response: For some of the plots, because the LD is missing for the lead SNP, we chose a variant that has LD.

Author responses to Reviewers comments:
REVIEWERS' COMMENTS:

Reviewer #1 (Remarks to the Author):

The authors have adequately addressed my comments.

Reviewer #2 (Remarks to the Author):

The authors have addressed all of my previous comments. The images in Supplementary Figure 5 didn't render correctly, but I'm sure this can be addressed.

Author Response: Thank you, we have revised Supplementary Figure 5.
